# Protective Effects of Micronutrient Supplements, Phytochemicals and Phytochemical-Rich Beverages and Foods Against DNA Damage in Humans: A Systematic Review of Randomized Controlled Trials and Prospective Studies

**DOI:** 10.1016/j.advnut.2023.08.004

**Published:** 2023-08-18

**Authors:** Michael F. Fenech, Caroline F. Bull, B. Jan-Willem Van Klinken

**Affiliations:** 1 Molecular Diagnostics Solutions, CSIRO Health & Biosecurity, Adelaide, South Australia, Australia; 2 Clinical and Health Sciences, Health and Biomedical Innovation, University of South Australia, Adelaide, South Australia, Australia; 3 Genome Health Foundation, North Brighton, South Australia, Australia; 4 School of Molecular and Biomedical Sciences, University of Adelaide, North Terrace, Adelaide, South Australia, Australia; 5 GSK Consumer Healthcare (now named Haleon), Warren, New Jersey, USA; 6 Brightseed, San Francisco, CA, United States

**Keywords:** DNA damage, DNA repair, DNA replication, chromosome aberrations, telomere, micronuclei, micronutrients, vitamins, minerals, phytochemicals, nutrition, beverages, foods

## Abstract

Accumulation of deoxyribonucleic acid (DNA) damage diminishes cellular health, increases risk of developmental and degenerative diseases, and accelerates aging. Optimizing nutrient intake can minimize accrual of DNA damage. The objectives of this review are to: *1*) assemble and systematically analyze high-level evidence for the effect of supplementation with micronutrients and phytochemicals on baseline levels of DNA damage in humans, and *2*) use this knowledge to identify which of these essential micronutrients or nonessential phytochemicals promote DNA integrity in vivo in humans. We conducted systematic literature searches of the PubMed database to identify interventional, prospective, cross-sectional, or in vitro studies that explored the association between nutrients and established biomarkers of DNA damage associated with developmental and degenerative disease risk. Biomarkers included lymphocyte chromosome aberrations, lymphocyte and buccal cell micronuclei, DNA methylation, lymphocyte/leukocyte DNA strand breaks, DNA oxidation, telomere length, telomerase activity, and mitochondrial DNA mutations. Only randomized, controlled interventions and uncontrolled longitudinal intervention studies conducted in humans were selected for evaluation and data extraction. These studies were ranked for the quality of their study design. In all, 96 of the 124 articles identified reported studies that achieved a quality assessment score ≥ 5 (from a maximum score of 7) and were included in the final review. Based on these studies, nutrients associated with protective effects included vitamin A and its precursor β-carotene, vitamins C, E, B1, B12, folate, minerals selenium and zinc, and phytochemicals such as curcumin (with piperine), lycopene, and proanthocyanidins. These findings highlight the importance of nutrients involved in (i) DNA metabolism and repair (folate, vitamin B_12_, and zinc) and (ii) prevention of oxidative stress and inflammation (vitamins A, C, E, lycopene, curcumin, proanthocyanidins, selenium, and zinc). Supplementation with certain micronutrients and their combinations may reduce DNA damage and promote cellular health by improving the maintenance of genome integrity.

**Statement of Significance**

To the best of our knowledge, this is the first time that the effects of essential nutrients, phytochemicals, and plant foods, in human interventions, on DNA damage have been comprehensively reviewed and succinctly summarized. The evidence provided supports the plausibility that appropriate nutritional intervention can substantially prevent DNA damage which is the most fundamental pathology underlying developmental and degenerative diseases. The main strength of this review is that it has brought together in a systematic manner the wealth of knowledge that has been accumulated over many decades on the impact of nutritional intervention on genome integrity in humans. Furthermore, this milestone review and its outcomes provide a solid foundation for the establishment of dietary reference values (DRVs) for DNA damage prevention. A key aspect of determining DRVs for DNA damage prevention is the achievement of high-level evidence that supplementation with specific nutrients required as cofactors for DNA metabolism or phytochemicals that may protect against genotoxic insults can substantially improve genome integrity. This systematic review provides the most complete existing high-level evidence that prevention of DNA damage in humans is achievable via appropriate intervention with essential nutrients, phytochemicals, and plant foods and that the efficacy and safety of such interventions can be verified using the best-validated biomarkers of DNA integrity that are currently available.

## Introduction

Damage to DNA can occur at any stage of life from conception onwards and can have devastating consequences caused by the resulting genetic mutations early in life (such as developmental defects) or later in life (such as cancers) [1, 2]. Cells with DNA damage, such as those containing multicentric chromosomes, micronuclei, or with excessive telomere shortening, become genomically unstable and/or senescent [3, 4]. Senescent cells increase with age, and their proinflammatory phenotype increases risk of other age-related diseases (e.g., infertility, auto-immune diseases, diabetes, chronic kidney disease, cardiovascular diseases, and neurodegenerative diseases) [[5], [6], [7]].

Over the past 50 or more years, several biomarkers have been developed that can measure DNA damage in humans *in vivo* at either the molecular or cytogenetic level. The earliest DNA damage biomarkers in humans were cytogenetic assays that measured structural or numerical chromosome aberrations in metaphases of primary lymphocyte cultures [8]. A simpler method to measure DNA damage at the chromosome level is the enumeration of micronuclei (MN) that originate from acentric chromosome fragments or whole chromosomes that fail to be segregated properly during mitosis and consequently are excluded from the 2 daughter nuclei (9). MN can be measured in erythrocytes, buccal cells, and lymphocytes [9, 10]. Entrapment of a chromosome in a micronucleus (MN), apart from loss or gain of genetic material, can also lead to either its fragmentation and rearrangement into a highly mutated chromosome or, if its membrane is disrupted, leakage of its DNA into the cytoplasm, with the consequential triggering of the proinflammatory cGAS-STING mechanism [10, 11].

At a more molecular level, DNA damage can be measured specifically in the telomeric region of the chromosomes [12]. Telomeres are (TTAGGG)_n_ repeat sequences at the ends of chromosomes that are required to prevent recombination of these ends and formation of multicentric chromosomes, a major cause of chromosomal instability. Telomeres can shorten as a result of DNA replication stress and natural attrition due to the incapacity of DNA polymerases to replicate the repeat at the very end of the telomere, or they can be completely lost due to unrepaired DNA strand breaks at the terminal ends of chromosomes [12, 13]. Telomere length can be measured at the single chromosome or nucleus level using molecular probes that hybridize to the TTAGGG sequence or by polymerase chain reaction (PCR) [[12], [13], [14]].

Other molecular biomarkers of DNA damage are DNA strand breaks. These can be measured at the single-cell level either by an electrophoresis technique called the “Comet” assay [15] or by immunohistochemistry to detect the formation of phosphorylated γH2AX, a protein that senses and binds to DNA strand break sites [16]. Other molecular techniques (e.g., HPLC, mass spectrometry) are used to measure adducts on DNA that are caused by reactive exogenous or endogenous molecules such as reactive oxygen species (ROS) [17]. PCR and advanced sequencing techniques are used to measure global or gene-specific methylation of DNA [18, 19]. PCR technology and gene sequencing have also been useful in enabling the measurement of point mutations and major deletions in mitochondrial DNA [20]. Most molecular DNA damage assays, including telomere assays, use DNA from white blood cells and, to a lesser extent, buccal cells or saliva.

Of these DNA damage biomarkers, only the lymphocyte chromosome aberration assay, lymphocyte cytokinesis-block micronucleus (CBMN) assay, telomere length, and gene-specific DNA methylation assays have been validated with regards to their prospective association with age-related degenerative diseases [[21], [22], [23], [24], [25]]. However, all of the biomarkers discussed in this review have been shown to be associated with various diseases in cross-sectional studies [[26], [27], [28], [29], [30], [31], [32], [33], [34]]. For example, the lymphocyte CBMN assay has been associated prospectively with pregnancy complications [35], cancer risk [23, 36] and cardiovascular disease [36, 37], and cross-sectionally in case-control studies with infertility, auto-immune diseases, obesity, diabetes, chronic kidney disease, coronary artery disease, and 7 different major cancers [[38], [39], [40], [41], [42], [43], [44], [45], [46]]. The validity of using DNA damage as a biomarker of nutritional status and disease risk was first reviewed in 2010 [33], and since then, evidence has continued to accumulate that supports the hypothesis that DNA integrity may be modified by nutritional intervention, which is the subject of this current review.

The consequences of increased DNA damage become manifest because insults to genome integrity can result in; (i) attenuation, deletion or silencing of housekeeping gene expression required for maintenance of normal metabolism, (ii) increased gene dosage that can result in metabolic disorders that aggravate susceptibility to disease, or (iii) senescence and inflammation caused by persistent DNA damage response signaling or leakage of DNA into the cytoplasm, triggering the cGAS-STING and senescence-associated secretory phenotype (SASP) proinflammatory mechanisms [3, 4, [47], [48], [49], [50], [51], [52], [53]]. Furthermore, susceptibility to endogenous or exogenous genotoxins increases risk of deleterious mutations. These may be more prevalent in those with defective DNA replication and/or impaired DNA repair capacity, both of which increase with aging [[54], [55], [56]]. There is, therefore, a strong interest in discovering efficacious strategies to optimize the maintenance of genome integrity.

**FIGURE 1:**
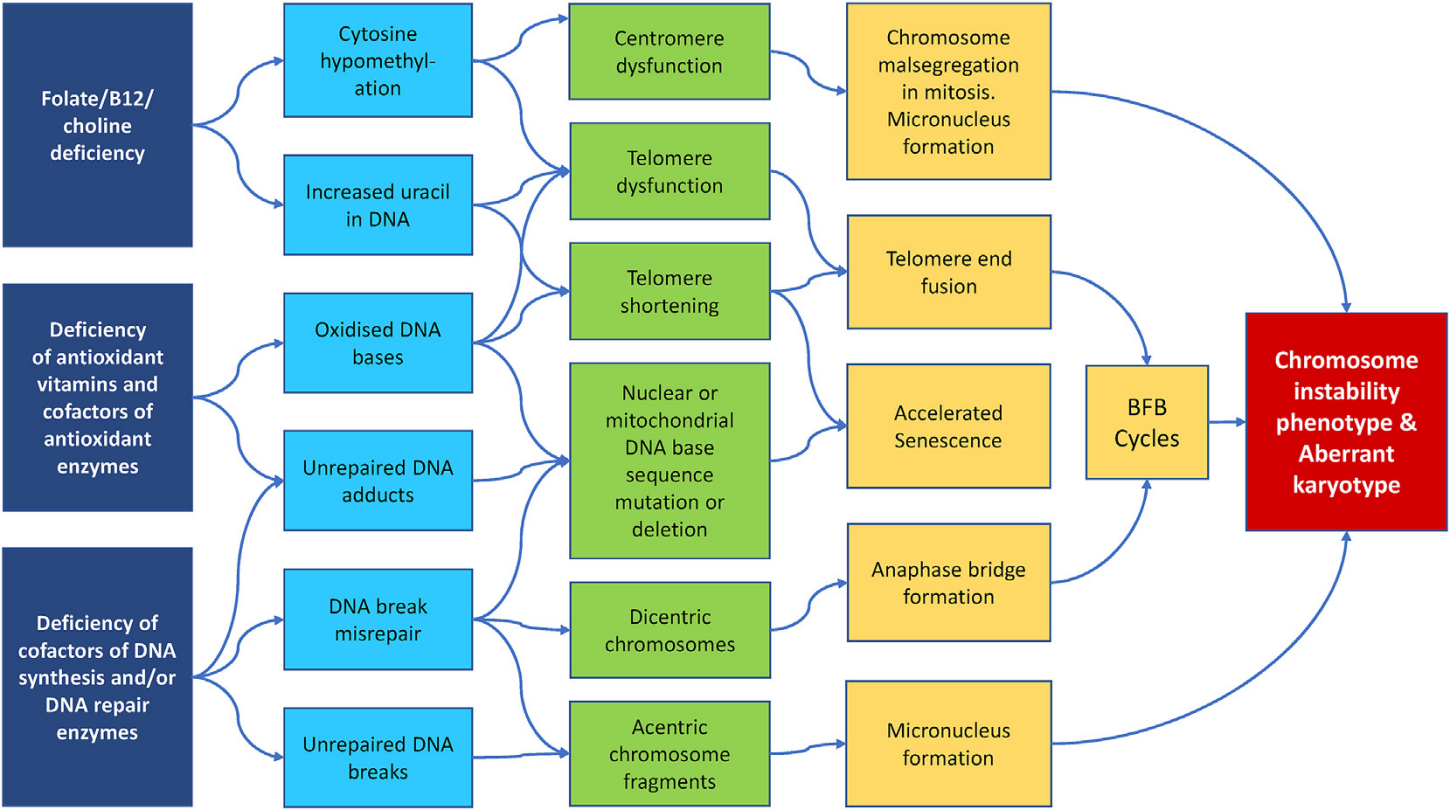
Mechanisms by which micronutrient deficiencies cause damage to the genome. BFB, breakage-fusion-bridge.

It is plausible that nutrient deficiency is one of the major causes of DNA damage in humans because several vitamins and minerals play important roles as substrates or cofactors in DNA replication and DNA repair [33] and also in the defense mechanisms involved in the neutralization of endogenous or exogenous genotoxins [57]. Some of the known mechanisms by which deficiencies in specific micronutrients can cause DNA damage and chromosomal instability are illustrated and summarized in Figure 1. This figure also illustrates the mechanistic relationship between the DNA damage biomarkers described above.

Several studies have shown that these DNA damage biomarkers are associated, in cross-sectional studies, with intake levels of a wide range of micronutrients, including vitamin C, vitamin E, various polyphenols, folate, vitamins B-2, B-3, B-6 and B-12, zinc, iron, magnesium, manganese, calcium and selenium, all of which have known mechanisms of action consistent with a role in protection against DNA damage [57, 58]. Therefore, there is a good basis for considering the possibility that supplementation with such nutrients may have a genome-protective effect in those who are deficient in these nutrients. However, this optimistic view also needs to be balanced by the need to know whether supplementation in those who already have adequate intake does not cause harm. In regard to this, a roadmap was proposed to determine DRV of individual micronutrients and nutrient combinations (nutriomes) for genome damage prevention [33].

The objective of this systematic review is to assemble and systematically analyze the currently available high-level evidence for the effect of supplementation with micronutrients and phytochemicals on baseline levels of DNA damage in humans. This review explores studies relating to supplements containing vitamins, minerals, and phytochemicals, as well as studies on fruits and vegetables containing high amounts of potentially genome-protective micronutrients and/or phytochemicals. The studies we evaluated used one or more of the DNA damage biomarkers listed above.

The review is structured so it first reports study results obtained for each DNA damage biomarker individually. Results are then presented for vitamins, minerals, phytochemicals, and beverages/foods using the combined results across all DNA damage biomarkers. This approach provides a better appreciation of which DNA damage biomarkers are most commonly used and the relative effects across biomarkers.

## Methods

### Search strategy

Between April and August 2017, a systematic literature search of the PubMed database was conducted to identify studies that explored the association between various nutrients and the established biomarkers of DNA damage. The search was guided by 2 plausible mechanisms by which nutrients support genome integrity and repair; (i) optimizing cellular capacity for high-fidelity DNA replication and repair in order to maintain genomic, epigenomic, and chromosomal stability and (ii) enhancing defense against the DNA-damaging effects of ROS. Only studies that used well-validated biomarkers, based on the strength of their association with developmental and/or degenerative diseases and/or mortality, were considered [22, 23, 31, 35, 37, [59], [60], [61], [62], [63]]. Only studies that used DNA damage biomarkers with an adequate level of evidence for their association with disease in any of the following 3 classes were accepted: Class I (Evidence from more than one prospective cohort study and several case-control studies): chromosome aberrations, micronuclei, telomere length, DNA methylation; Class II (Evidence from only one prospective cohort study and several case-control studies): DNA strand breaks assay and/or Comet assay; Class III (Evidence from case-control studies only): DNA oxidation, mitochondrial DNA mutations and/or copy number [[21], [22], [23], [24], [25], [26], [27], [28], 30, 31, [33], [34], [35], [36], [37], [38], [39], [40], [41], [42], [43], 45, 46, [59], [60], [61], [62], [63], [64], [65], [66]]. These biomarkers can be measured using tissues and cells that are relatively easy to collect, such as lymphocytes, leukocytes, and erythrocytes in blood samples, buccal cells collected using a toothbrush or spatula, or from saliva.

Search strings were developed using defined combinations of (i) nutrition terms (“nutrition/nutrients,” “phytochemicals,” “phytonutrients,” “minerals,” “radioprotection,” “antioxidant,” “vitamins,” “minerals,” “amino acids,” “fatty acids,” “malnutrition,” and “protein malnutrition”), (ii) DNA damage terms (“chromosome aberrations,” “micronuclei,” “DNA strand breaks,” “DNA oxidation,” “telomere length,” “telomerase,” “DNA methylation,” and “mitochondrial DNA”), and (iii) tissue terms (“lymphocyte,” “leukocyte,” and “buccal”). Details of the search strategy are provided in Supplemental Table 1. The review was reported following the Preferred Reporting Items for Systematic Reviews and Meta-Analyses (PRISMA) statement [67].

### Study selection

**FIGURE 2:**
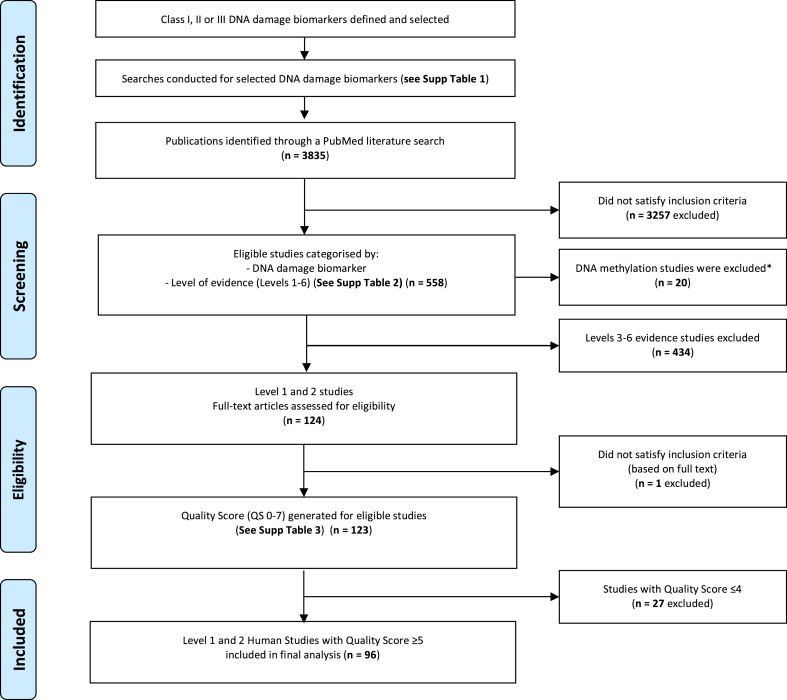
Publication selection process ∗DNA methylation studies were excluded at screening because of the great heterogeneity between studies regarding the techniques used to measure DNA methylation either at the gene-specific level or globally.

The process of study selection is detailed in Figure 2. Studies in the initial screening included interventional, prospective, cross-sectional, or in vitro human studies or in vivo studies in animal models. All studies were published in peer-reviewed journals between 1965 and August 1, 2017. Initial screening was based on full abstracts, which were extracted and reviewed by either MFF or CFB (not both).

The inclusion criteria for studies identified in the PubMed search results were the following:

• the study measured one or more of the selected biomarkers of DNA damage;
• the study tested the effect of a single nutrient, nutrient complex/combination, extract, metabolic precursor, or whole food/beverage. (Studies using synthetic analogs, whole diet, and/or lifestyle were excluded unless they were uniquely informative);
• study conducted in vitro, in vivo, human or animal model; and
• supplement or food consumed orally. (Studies with injected supplements were excluded).

Selected abstracts were then tabulated and categorized by level of evidence (Levels 1–6) according to predetermined criteria (Supplemental Table 2). Only randomized, controlled (Level 1), and longitudinal intervention (prospective) (Level 2) studies that met the following additional criteria were then selected for further detailed review:

• sufficient data provided in the paper to allow critical analysis;
• sufficient detail provided on vitamin/mineral supplement composition; and
• not a duplication of the same study/data published elsewhere.

Ten studies evaluating the effects of food and micronutrients on global DNA methylation and 10 studies evaluating their effects on gene-specific DNA methylation were identified in the initial screening. Due to the diversity of methods used to measure global or gene-specific methylation, the number of Level 1 and Level 2 studies identified was insufficient to allow meaningful interpretation of the results. Furthermore, a systematic review and meta-analysis published in 2018 [68] of the effect of randomized controlled trials (RCTs) on DNA methylation concluded that there is limited evidence from intervention studies of the effects of dietary factors, other than folic acid, on DNA methylation in humans. It was also concluded that the use of multiple different assays and investigations of different genomic loci makes it difficult to compare or combine data across studies [68]. Accordingly, DNA methylation studies were omitted from further review and analysis.

### Data extraction, quality assessment, and data analyses

All eligible abstracts following initial screening (*n =* 558) were tabulated according to whether they investigated a vitamin, mineral or phytochemical, single nutrient, or combination of multiple nutrients, beverages, or foods. Studies categorized as evidence Levels 3, 4, 5, or 6 were excluded from further analysis.

A quality score (QS) was then generated for all eligible Level 1 and 2 studies. The QS was based on the study design, the number of subjects per group, duration of the intervention, the use of a placebo control, study cohort age- and/or gender-matching, and whether the *P* value for change in the main outcome measure from baseline was statistically significant (maximum score 7; Supplemental Table 3).

For the final analysis, Level 1 and Level 2 studies with a QS ≥ 5 were considered sufficiently rigorous to be useful in identifying nutrients that could be efficacious for inclusion in a dietary supplement to improve genome integrity. A total of 96 studies were shortlisted and analyzed for the size and direction of the effect of the vitamins, minerals, and/or phytochemicals on the biomarkers evaluated (Figure 2).

## Results

**FIGURE 3:**
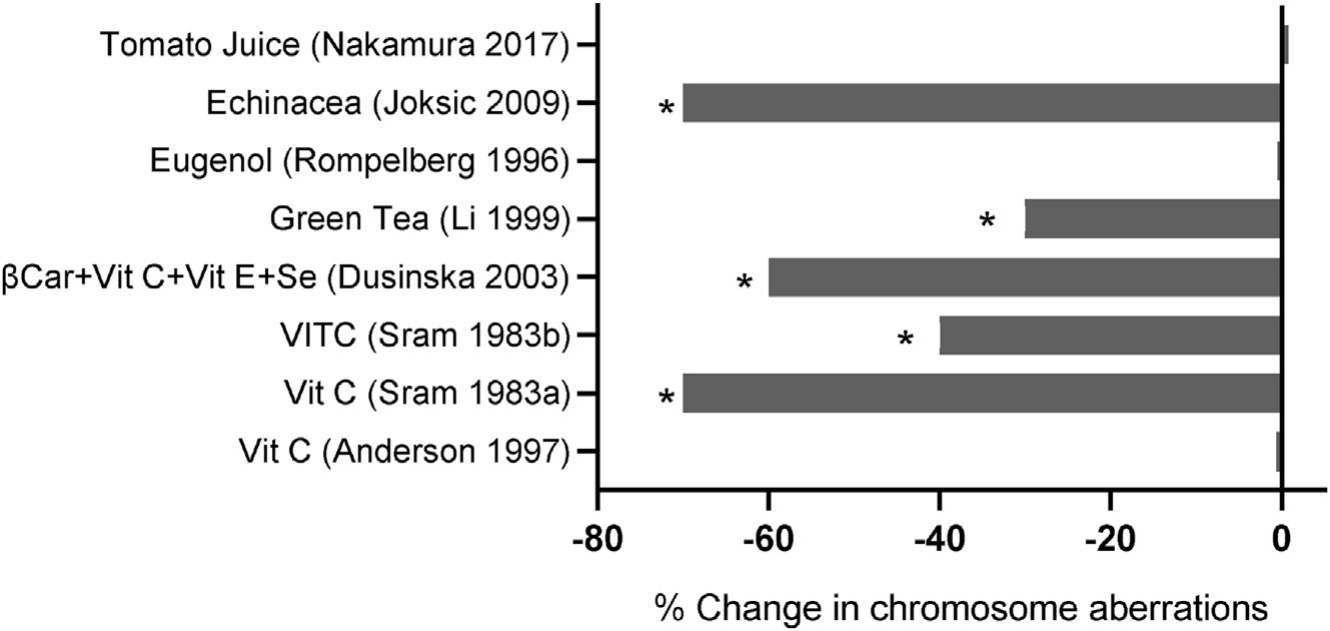
Percentage change in chromosome aberration (CA) frequency in subjects supplemented with one of the following: tomato juice, Echinacea, eugenol, green tea extract, vitamin C, a combination of β-carotene + vitamin C + vitamin E + selenium. Data shown are from Level 1 or Level 2 studies with quality scores of 5 to 7. (∗ *P* < 0.05)

**FIGURE 4:**
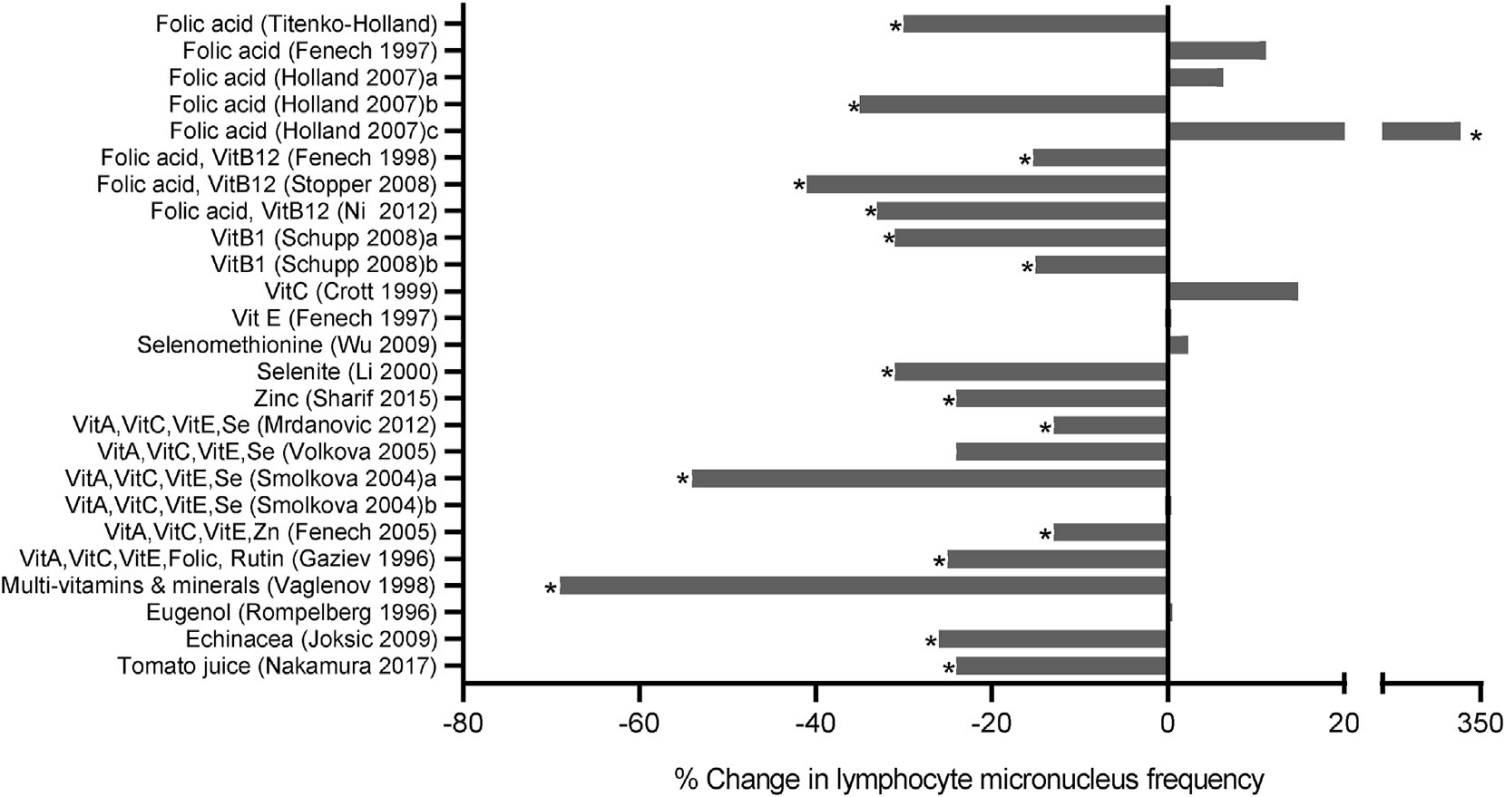
Percentage change in lymphocyte micronucleus (MN) frequency in subjects supplemented with individual micronutrients or phytochemicals, micronutrient combinations, or tomato juice. Studies with an “a,” “b,” or “c” indicates results for different groups within the same study. Data shown are from Level 1 or Level 2 studies with quality scores of 5 to 7. (∗ *P* < 0.05)

**FIGURE 5:**
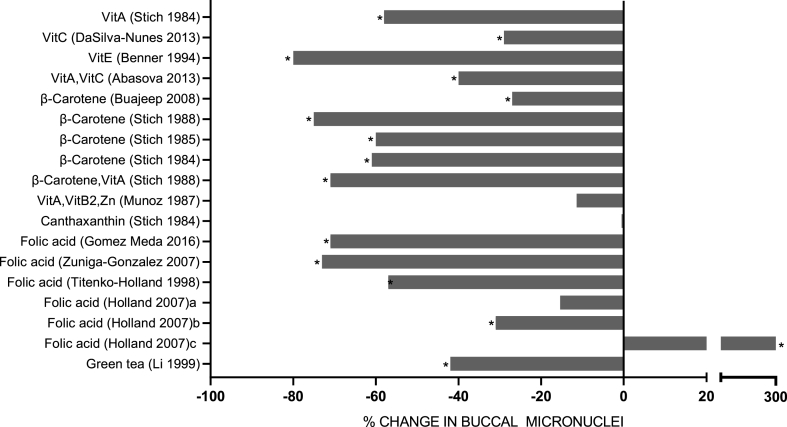
Percentage change in buccal micronucleus (MN) frequency in subjects supplemented with individual micronutrients, phytochemicals, or micronutrient combinations. Studies with an “a,” “b,” or “c” indicates results for different groups within the same study. Data shown are from Level 1 or Level 2 studies with quality scores of 5 to 7. (∗ *P* < 0.05)

**FIGURE 6:**
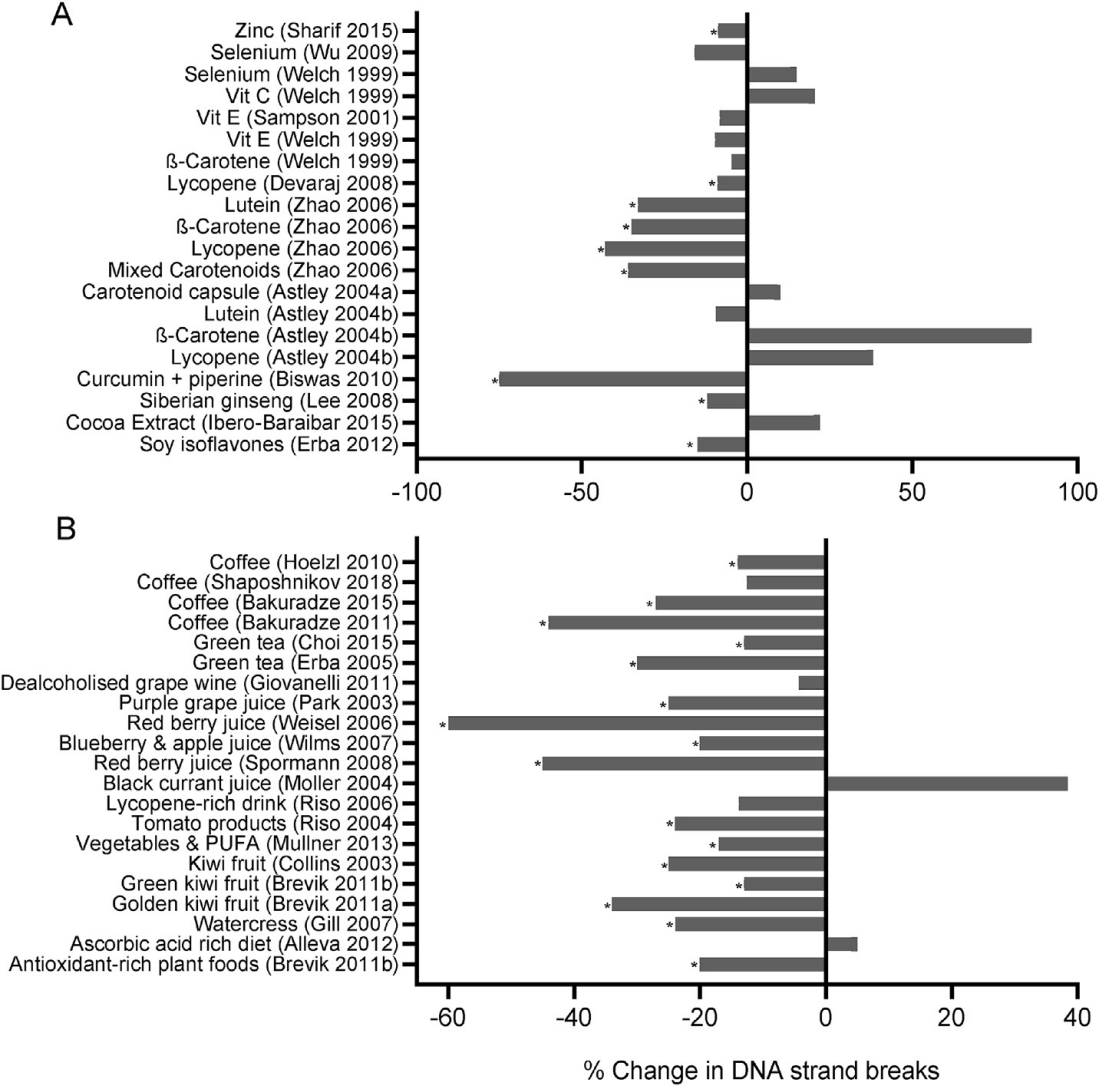
Percentage change in leukocyte DNA strand breaks, determined by Comet assay, in [A] subjects supplemented with individual micronutrients, phytochemicals or micronutrient combination supplements, and [B] plant-based beverages or whole foods. Data shown are from Level 1 or Level 2 studies with quality scores of 5 to 7. (∗ *P* < 0.05)

**FIGURE 7:**
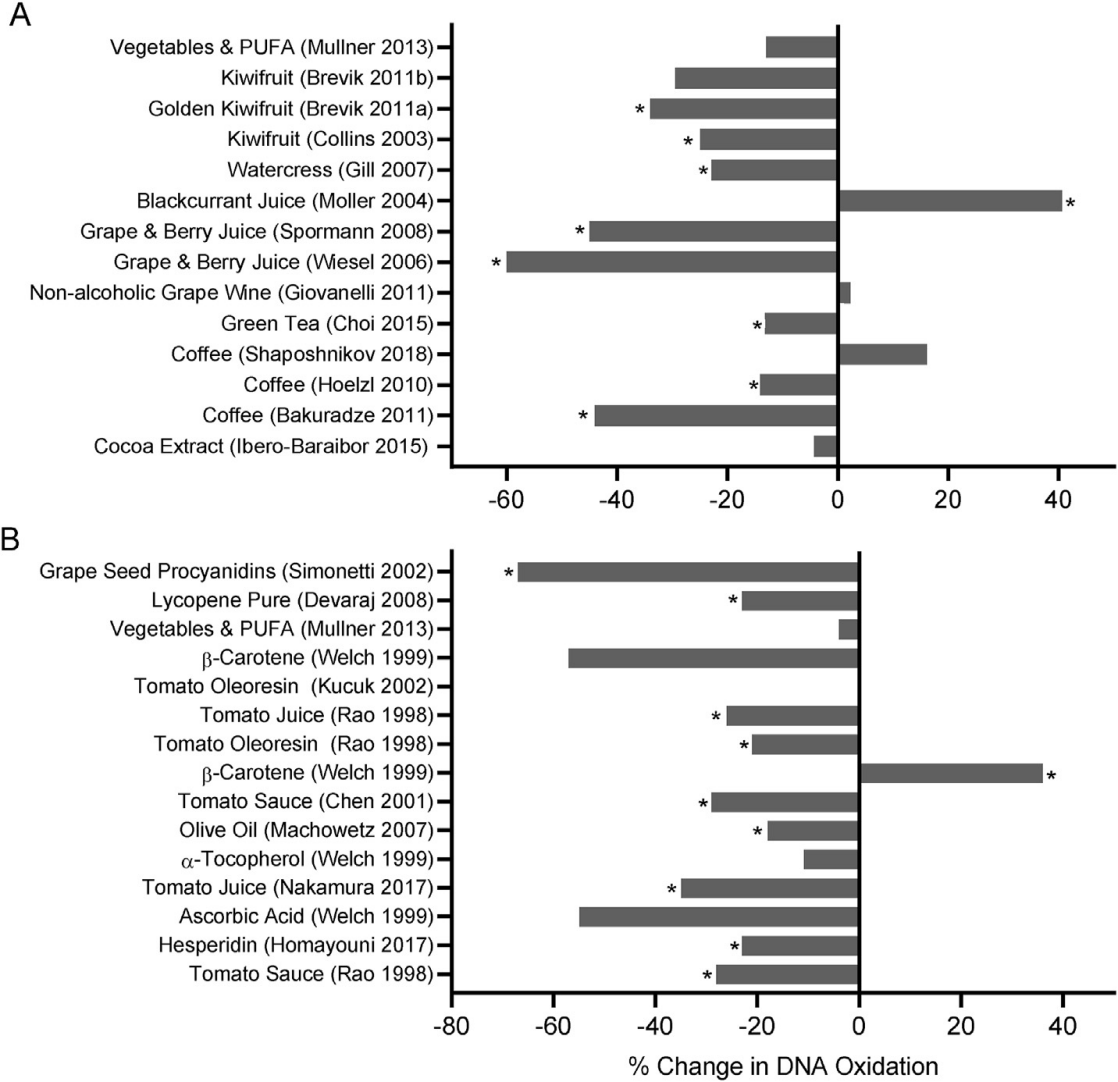
Percentage change in DNA oxidation following intervention with various plant-based extracts, beverages, and fruits, measured by [A] FPG or ENDOIII Comet assay; or [B] HPLC or MS. Data shown are from Level 1 or Level 2 studies with quality scores of 5 to 7. (∗ *P* < 0.05)

**FIGURE 8:**
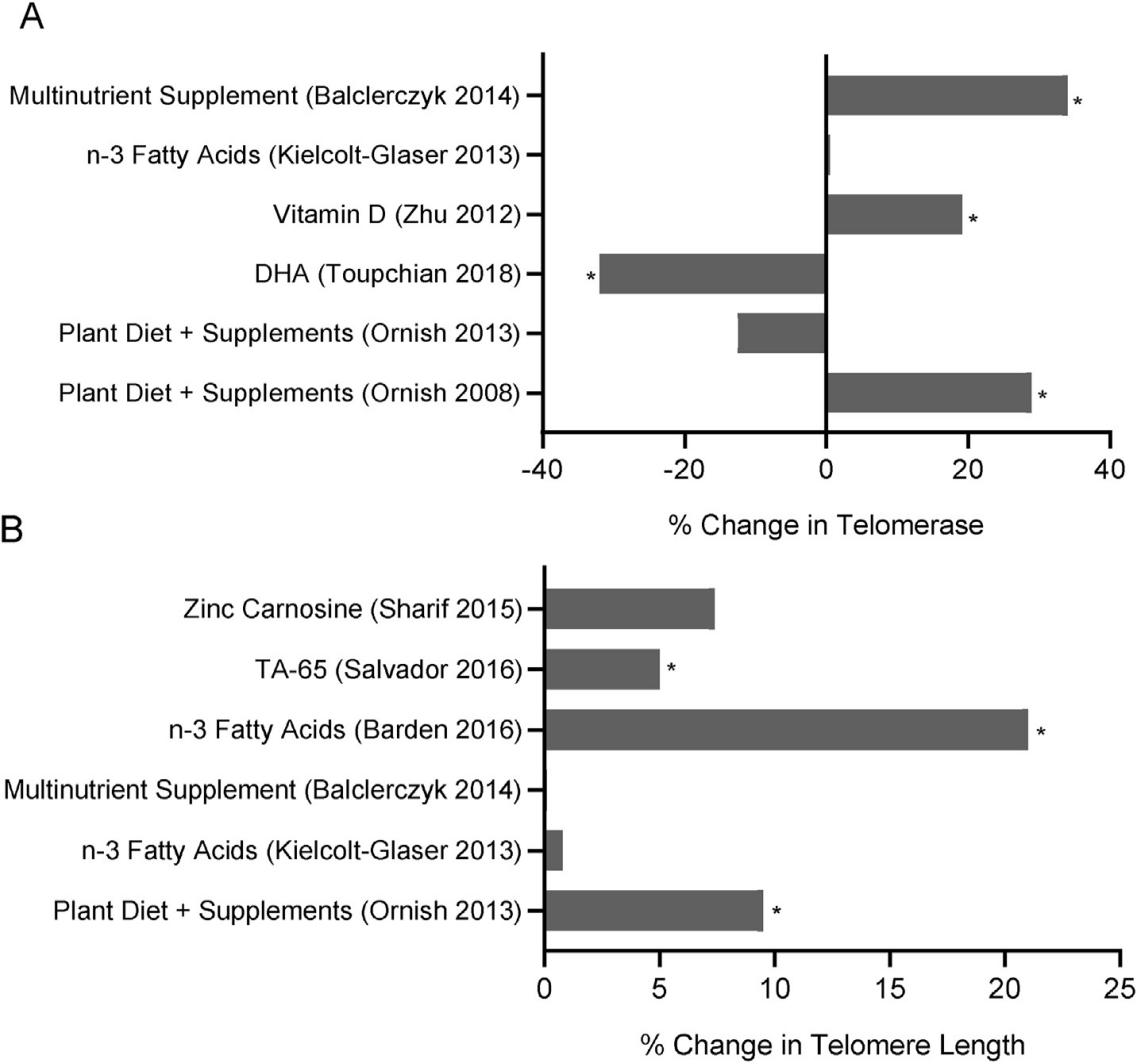
Percentage change in (A) telomerase activity and (B) telomere length after various nutrition interventions. The telomere length data for the Barden 2016 study were corrected for neutrophil count. Data shown are from Level 1 or Level 2 studies that have quality scores of 5 to 7. (∗ *P* < 0.05)

The data from the papers describing the selected 96 Level 1 and Level 2 human interventions with a QS ≥ 5 was further refined because (i) some of them performed the same intervention in different groups with different health conditions, (ii) some reported results on more than one DNA damage biomarker, and (iii) some studies had more than one intervention arm because they investigated more than one nutrient or food/beverage. Consequently, the 96 shortlisted papers yielded a total of 134 individual interventions (Table 1). The results of these 134 interventions are illustrated in FIGURE 3, FIGURE 4, FIGURE 5, FIGURE 6, FIGURE 7, FIGURE 8 and described briefly below. Further details of these interventions are provided in Supplemental Tables 4–9.

**TABLE 1:** Number of papers and interventions reporting results across the various DNA damage biomarkers used in the selected studies

DNA damage biomarkers	Number of papers (%)	Number of interventions (%)
Chromosome aberrations	8 (8.3)	8 (5.9)
Micronuclei in lymphocytes	22 (22.9)	25 (18.6)
Micronuclei in buccal cells	13 (13.5)	18 (13.4)
DNA strand breaks Comet assay	33 (34.4)	41 (30.6)
DNA oxidation	10 (10.4)	29 (21.6)
Telomere length or telomerase	9 (9.4)	12 (8.9)
Mitochondrial DNA mutation	1 (1.0)	1 (0.7)
	Total 96 (100)	Total 134 (100)

### Chromosome aberrations in lymphocytes

A total of 9 Level 1 and 2 studies on chromosome aberrations (CAs) were identified, of which 8 were of a sufficiently high quality (QS ≥ 5) to be considered for evaluation. Three studies investigated the effect of vitamin C, with 2 showing a strong reduction (40%, 70%) in CAs, whereas the other showed no effect [66, 69, 70]. An RCT using a combination of β-carotene, vitamin C, vitamin E, and selenium showed a robust (60%) reduction in CAs [71]. RCTs performed using either a green tea extract or eugenol resulted in a 30% reduction in CAs in the former and no effect in the latter [72, 73]. Supplementation with Echinacea polyphenols in radiology workers resulted in a 70% reduction of CAs [74]. Daily consumption of tomato juice significantly reduced dicentric chromosomes induced by ex vivo exposure to 0.5 Gy of ionizing radiation but had no impact on baseline levels [75]. Figure 3 and Supplemental Table 4 provide further detail on these studies.

### Micronuclei in lymphocytes

A total of 35 Level 1 and 2 studies on micronuclei (MN) in lymphocytes were identified, of which only 22 achieved a QS of 5 to 7. Supplementation with vitamin C or vitamin E had no impact on MN frequency [76, 77]. Supplementation with vitamin B1 (as prodrug benfotiamine) in hemodialysis patients reduced MN by 15% and 31% [78, 79]. Another intervention in these patients showed that both folic acid and the combination of folic acid with vitamin B12 attenuated MN frequency by 25% and 41%, respectively [80]. In contrast, supplementation of healthy elderly male subjects with folic acid had no impact on MN frequency, but the combination of folic acid with vitamin B12 reduced MN frequency by 15.3% in healthy young adults and by 33.5% in healthy older adults [[81], [82], [83]]. A depletion-repletion dose-response intervention with folic acid and tight control of background diet in postmenopausal women resulted in a 30% decrease in MN frequency after repletion [84]. Intervention in children with 1 mg/d of folic acid for 4 wk led to mixed results, with no effect observed in healthy children, a 35% decrease in MN frequency in children with Crohn’s disease, and a remarkable 340% increase in MN frequency in children with ulcerative colitis [85].

Selenium (Se) in the selenomethionine form did not alter MN frequency in a cohort that was not deficient in Se [86]. In contrast, supplementation with the sodium selenite form of Se in a Chinese high-risk cohort for liver cancer significantly reduced MN frequency by 31% [87]. An RCT using zinc carnosine, in healthy elderly subjects in Australia with subclinically low zinc status resulted in a significant 24.2% reduction in the incidence of MN in lymphocytes [88].

Three interventions investigated the effect of phytochemicals on MN in lymphocytes. One study explored the effect of tomato juice consumption and reported a reduction in baseline MN and radiation-induced (in vitro*)* MN frequency in the treatment group by a margin of -33% and -17%, respectively [75]. In another investigation, it was observed that daily consumption of *Echinacea purpurea* polyphenol supplements reduced MN frequency by 26% in a cohort of radiology technicians with elevated lymphocyte chromosome aberrations [74]. Eugenol supplementation, however, had no effect on DNA damage measured as MN in lymphocytes in a small cohort of healthy young males [73, 89].

Five interventions used a combination of vitamins A, C, and E together (2 studies), plus either zinc alone, with the addition of folate and rutin, or with the addition of selenium (3 studies). The outcomes of these studies on MN frequency were a 13% reduction, 25% reduction, 54% reduction, 13% reduction, and no effect, respectively [[90], [91], [92], [93], [94]].

The remaining intervention study investigated the effect of a more complex supplement containing vitamins A, B1, B2, B3, B4, B5, B6, B7, B9, B12, C, D, E, K, and 18 minerals, including zinc and selenium) which resulted in a 69% reduction in MN frequency [95]. Figure 4 and Supplemental Table 5A provide further detail on these studies.

### Micronuclei in Buccal Cells

All 13 studies identified using MN in buccal cells as the DNA damage biomarker achieved a QS of ≥5. Studies on the effect of micronutrients on MN in buccal cells were initiated by Stich et al. [96] showing that vitamin A and β-carotene reduced MN by 58% and 61%, respectively, in chewers of a carcinogenic mixture of betel nut and tobacco in the Philippines. This was followed up by another study on Canadian Inuits using smokeless tobacco, which showed a 60% reduction in MN in buccal cells after supplementation with β-carotene [97]. Another study on betel quid/tobacco chewers in India by the same group showed a reduction of 75% and 71% in buccal MN frequency after supplementation with either β-carotene or a combination of β-carotene with vitamin A [98]. In patients with (inflammatory skin condition) lichen planus, supplementation with β-carotene reduced MN in normal buccal cells by 27%, and in cells from lichen planus sites, MN were reduced by 78% [99]. An intervention in China aimed at reducing precancerous lesions in the upper digestive tract showed that supplementation with a combination of vitamin A, vitamin B2, and zinc had no effect on buccal MN but did show a 39% reduction in esophageal MN [100]. Supplementation with a combination of vitamin A and vitamin C resulted in a 39% reduction in buccal MN frequency in healthy young adults [101].

In a leukoplakia (oral mucosal lesion) chemoprevention trial in the USA, participants who were supplemented with vitamin E experienced an 80% reduction in buccal MN, and in leukoplakia cells, there was a 63% reduction in MN [102].

An intervention with vitamin C on obese women undergoing treatment with amphetamine anorectic drugs showed that the drugs increased MN in the buccal mucosa, but concomitant intake of vitamin C caused a 29% reduction in this DNA damage biomarker [103].

Four studies investigated the effect of folic acid on frequencies of buccal MN. A depletion-repletion intervention in healthy postmenopausal women showed that buccal MN decreased by 57% after folic acid supplementation [84]. In 2 separate studies, supplementation with folic acid reduced buccal MN frequency by 71% and 73% in patients with diabetes [104, 105]. A unique study in children with inflammatory bowel disease showed that intervention with folic acid supplementation caused a 31% reduction in buccal MN in Crohn’s disease cases and a remarkable 300% increase in buccal MN in those with ulcerative colitis [85].

In a chemoprevention trial of oral leukoplakia, it was shown that green tea supplementation reduced MN in leukoplakia by 49% and by 42% in normal buccal cells [72]. Figure 5 and Supplemental Table 5B provide further details on these buccal MN assay studies.

### DNA strand breaks in leukocytes measured by Comet assay

Forty-five papers were published describing the effect of interventions on DNA strand break (DSB) levels in blood leukocytes or purified lymphocytes. Of these, 33 interventions achieved a QS of 5 to 7, and these are described briefly below. The outcomes for micronutrient supplements are illustrated in Figure 6[A], and those for beverages and whole foods are in Figure 6[B].

Supplementation with 20 mg/d of zinc resulted in a 5.7 % increase in plasma zinc concentration and a reduction of approximately 7.5% to 8.8% in DSB levels in healthy elderly subjects [88]. An RCT in healthy older men who increased selenium (Se) intake by consuming 3 puffed wheat biscuits made using biofortified wheat was found to increase plasma Se concentration, but there was no change in Comet assay DSBs, or biomarkers of oxidative stress [106]. An RCT with selenium selenite also reported a null effect result [107].

A double-blind 8-wk RCT performed on type 2 diabetes cases and healthy controls showed that daily intake of 400 IU vitamin E had no effect on DSBs in either group [108]. Welch et al. used a sequential crossover RCT of 5 intervention treatments to investigate the independent effect of daily intake of vitamin C, vitamin E, a combination of vitamin C and vitamin E, Se, and β-carotene. However, none of the treatments exerted a significant change in the DSB level of leukocytes [107].

Astley et al. [109] first reported a parallel RCT comparing a mixed carotene daily supplement equivalent to 1 carrot per day, a cooked carrot, tinned mandarins, or a 60 mg vitamin C supplement; however, none of these treatments affected DSBs after 21 d of intervention. They also performed 3 separate RCTs to test the effect of daily supplementation with either 15 mg β-carotene, 15 mg lutein, or 15 mg lycopene and again found no effect of carotenoid supplementation on DNA strand breaks [110]. However, Zhao et al. [111] reported a significant reduction of DSBs in each of 4 carotenoid intervention arms: -33% for lutein, -35% for β-carotene, -43% for lutein, -36% for the combination, and no change in the placebo after 57 d of intervention. Devaraj et al. [112], who did a dose-response RCT of lycopene supplementation (0, 6.5, 15, or 30 mg/d), found a significant 8.9% reduction in DSBs only with the highest dose.

Four studies were identified that focused on the effects of supplements containing other types of phytochemicals with antioxidant properties. The study of Biswas et al. [113] showed that a 3-mo supplementation with curcumin, together with piperine, reduced DSBs by 75% in subjects exposed to arsenic. A 6-mo RCT in healthy postmenopausal women with Siberian Ginseng resulted in a significant 12% reduction in DSBs measured as ‘tail DNA’ in the Comet assay [114]. A 4-wk RCT in overweight/obese subjects in Spain showed that consumption of a 1.4 g/d cocoa supplement added to a 15% calorie-restricted diet did not produce significant changes in DSBs [115]. Erba et al. investigated the effect of daily supplementation with 80 mg soy isoflavone for 6 mo in healthy pre- and postmenopausal women and reported a significant 11% to 15% reduction in DSBs [116].

Fourteen RCTs were dedicated to measuring the effect of plant-based beverages on DSBs. Four explored the effect of drinking different kinds of coffee. A crossover RCT in healthy men who daily drank 750 ml of a freshly brewed Arabica coffee blend rich in both green and roasted constituents for 4 wk resulted in a 39% reduction in DSBs and a 44% reduction in oxidized DNA [117]. A second RCT using the same study design as above but this time using dark roast coffee, reported a 27% reduction in DNA damage in the coffee treatment group [118]. Hoelzl et al. [119] reported that daily consumption of 800 ml of instant coffee (containing 35% green and 65% roasted coffee extracts) resulted in a nonsignificant 14.1% decrease in oxidized DNA bases using the FPG Comet assay. Similarly, a daily intake of 3 or 5 cups of coffee (100% roasted, Arabica) for 8 wk did not induce significant changes either in endogenous DSBs or oxidized DNA bases measured by Comet assay [120].

The RCT of Erba et al. [121] showed that daily consumption of 2 cups of green tea for 42 d significantly reduced DSBs by 30% in healthy female young adults. Choi et al. [122] performed an RCT showing that daily green tea consumption for 12 wk in type 2 diabetic subjects resulted in a significant reduction in DSBs (-13.0%) and DNA oxidation (-13.2%).

A further 6 papers reported on the outcomes of interventions with nonalcoholic fruit-based drinks, which are briefly described below. Giovanelli et al. [123] reported that consumption of nonalcoholic grape wine, with low or high proanthocyanidin concentration, in postmenopausal women had no effect on DSBs or oxidized DNA bases measured using the Comet assay. In South Korea, an intervention in healthy adults showed that drinking 480 mL/d of pure, undiluted grape juice for 8 wk in a prospective study reduced DSBs by 25% in smokers and 18% in nonsmokers [124]. An RCT with adult males who daily consumed 700 ml of a mixed grape and berry juice beverage for 4 wk showed a 60% reduction in DNA damage mainly due to the prevention of DNA base oxidation [125]. Using a similar study design, Spormann et al. [126] reported a significant 45% decrease in total DNA damage (mainly oxidative DNA damage) in clinically stable hemodialysis patients who consumed 200 ml/d of mixed grape and berry juice. The effect of consuming a blueberry and apple juice mixture (1 L/d) for 4 wk was investigated in Holland in healthy adults, which showed a 20% reduction in DSBs in lymphocytes following ex vivo oxidative challenge [127]. Moller et al. [128] tested the DNA-protective effects of a 3-wk intervention with black currant juice or an anthocyanin drink relative to a control drink. They observed that FPG-sensitive sites (i.e., DNA oxidation) actually increased by 41% in the black currant juice treatment group, whereas DSBs remained unchanged, and no changes were observed in the other 2 groups.

Riso et al. performed a 3-wk intervention with commonly consumed tomato products (raw tomato, tomato sauce, and tomato paste) in healthy female adults who were kept on an otherwise low carotenoid basal diet during the trial. Measurements before and after the intervention showed a significant 24% reduction in DSBs in lymphocytes after ex vivo oxidative challenge [129]. The same group next investigated the bioefficacy of 1 drink per day of a tomato-based drink in a double-blind crossover RCT in young, healthy adults and found no effect on endogenous DSBs in lymphocytes [130].

A crossover RCT in healthy adults (50% smokers) showed that daily eating of 85 g raw watercress for 8 wk resulted in a 17% reduction in endogenous DSBs, 23% less oxidative DNA damage in vivo, and 9.4% fewer DSBs after ex vivo hydrogen peroxide challenge; furthermore, these effects were significantly stronger in smokers [131].

Three RCTs on the effects of kiwi fruit were reported. In the first RCT, healthy adult subjects consumed 1, 2, and 3 kiwifruits (for 21 d each time) which resulted in significant 20% to 30% reductions of endogenous oxidized purines or pyrimidines in DNA (measured by FPG and Endonuclease III (Endo III) Comet assays respectively), as well as improved resistance to DSB induction in ex vivo hydrogen peroxide challenge [132]. The second RCT performed with a new golden variety of kiwifruit, as a supplement to the western diet, showed significant reductions in vivo endogenous FPG-sensitive sites, in in vivo endogenous Endo III-sensitive sites, and improved ex vivo resistance to hydrogen peroxide-induced oxidation [133]. In the third study, 8 wk of intervention supplementing the habitual diet with either 3 (green) kiwi fruits per day or antioxidant-rich plant products in a cohort of older male smokers caused a reduction in DSBs by 13% in the kiwifruit group, and 20% in the antioxidant-rich plant group, but there was no significant effect on endogenous DNA oxidation measured by FPG or Endo III-sensitive sites in the Comet assay [134].

Mullner et al. [135] reported that supplementing the habitual diets of type 2 diabetics with 300 g of vegetables per day and 25 mL of plant oil-rich PUFA per day reduces DSBs by 17% and FPG-sensitive sites by 13% in the treated diabetics.

Another whole food RCT showed that consuming a diet high in ascorbic acid-rich foods (equivalent to an additional 600 mg/d ascorbic acid) did not affect DSBs measured by Comet assay in subjects exposed to increased oxidative stress induced by hyperbaric oxygen treatment [136].

Figures 6[A], 6[B], and Supplemental Table 7 provide further details on the studies reporting on the intervention effects on DSBs measured using the Comet assay.

### DNA Oxidation

Fourteen of the studies described in the previous section on DSBs measured by Comet assay also reported on DNA oxidation effects using the FPG and/or Endo III versions of the assay. In this assay, oxidized purine and oxidized pyrimidine bases in DNA are excised, and the DNA backbone is nicked by treatment with the FPG and/or the Endo III DNA damage repair enzymes, respectively. The resulting DNA breaks are then measured by the standard Comet assay method. The DNA oxidation data from these Comet assay studies is described in Supplemental Table 7 together with the DSB data and shown separately graphically in Figure 7 [A].

An additional set of 10 DNA oxidation studies (Level 1 and 2 studies with quality scores 5 to 7) were identified that used alternative DNA oxidation assays, including measuring oxidized guanine in body fluids such as urine, plasma, or serum, or alternatively, measuring oxidized guanine or oxidized uridine in DNA from cells in the body. These methods involved the use of different analytical techniques such as HPLC, mass spectrometry, and ELISA. Five of the investigations related to purified lycopene or the consumption of tomato products rich in lycopene. Four of the remaining 5 investigated plant products (olive oil, hesperidin, grape procyanidins, vitamins, minerals), whereas the remaining one explored the effects of a diet rich in vegetables and polyunsaturated fatty acids (PUFAs). These 10 DNA oxidation studies are described briefly below.

Devaraj S et al. [112] performed an RCT to test the effect of supplementation with purified lycopene and reported a significant 23% reduction in urinary 8-hydroxy deoxyguanosine (8-OHdG) in those consuming 30 mg lycopene per day.

Using an RCT with multiple crossover design, Rao and Agarwal [137] tested the efficacy of 5 tomato-based products, which included (i) tomato juice (daily lycopene intake (DLI) 50.4 mg), (ii) spaghetti sauce #1 (DLI 20.5 mg); (iii) spaghetti sauce #2 (DLI 39.2 mg); (iv) oleoresin capsule #1 (DLI 75 mg), and (iv) oleoresin capsule #2 (DLI 150 mg). DNA oxidation measured as 8-oxo deoxyguanosine (8-oxodG) in lymphocyte DNA was reduced by 28%, 26%, and 21% relative to control after consumption of spaghetti sauce #2, tomato juice, and oleoresin capsule #2, respectively; however, none achieved statistical significance.

Nakamura et al. [75] reported the results of a prospective intervention in which 10 young, healthy adults (5 men, 5 women) consumed 190 g tomato juice (containing 17 mg lycopene and 0.25 mg β-carotene) daily for 3 wk; DNA oxidation (measured as extracellular 8-oxodG in plasma) was reduced by 35% after tomato juice consumption and returned to baseline level after washout; however, these effects were not statistically significant.

Chen et al. [138] investigated the antioxidant effects of consuming 30 mg lycopene/day from tomato-sauce-based foods in 32 prostate cancer patients before prostatectomy surgery using a prospective intervention. There was a significant 22% reduction in DNA oxidation in leukocytes, and there was also a significant 29% lower DNA oxidation in prostate tissue in the intervention group.

Kucuk et al. [139] investigated the effect of lycopene supplementation on DNA oxidation in early stage localized prostate cancer cases. The treatment group consumed oleoresin extract containing 15 mg lycopene twice daily for 3 wk which raised plasma lycopene but did not cause significant changes in DNA oxidation measured as 5-hydroxy methyl deoxyuridine (5-OHmdU) in peripheral blood lymphocytes.

The effect of 500 mg/d hesperidin supplementation was investigated in a 6-wk RCT in 64 adults with type 2 diabetes who were randomized either to treatment or placebo. In the treatment group, serum 8-OHdG decreased by 23% from a baseline concentration of 14.32 ± 6.4 ng/ml versus 11.00 ± 7.0 ng/ml after the 6-wk intervention, but there was no change in the placebo group [140].

Olive oil is a key component of the Mediterranean diet. Machowetz et al. [141] conducted a double-blind RCT crossover design study involving 182 healthy adult men to test the hypothesis that daily consumption of 25 ml of olive oil with high phenolic content reduces DNA oxidation. The outcome of the interventions was that consumption of olive oil reduced urinary 8-oxodG by 18%, but this effect was not influenced by the phenolic content of the olive oil.

Procyanidins are oligomers of (epi)catechin and (epi)catechin gallate units found in several plant foods, and they are particularly rich in grape seeds. Simonetti et al. [142] tested the hypothesis that daily consumption of 110 mg grape seed procyanidins protects against DNA oxidation using a prospective study. There was a 67% reduction in lymphocyte DNA oxidation after the 30-d intervention relative to baseline, and this effect remained unchanged after the 7-d washout period.

The effects of micronutrients at doses that may exert antioxidant effects in vivo (ascorbic acid 350 mg; RRR-α-tocopherol 250 mg; β-carotene 60 mg; selenium 80 ug as sodium selenite, and combined ascorbic acid 350 mg + RRR-α-tocopherol 250 mg) were explored by Welch et al. [107] in a RCT with multiple cross-overs. Twenty-one men (12 smokers and 9 nonsmokers) participated in the intervention. Each treatment period lasted 4 wk with a 4-wk washout period between treatments, during which they received a placebo. DNA oxidation measured as the amount of 8-OHdG in leukocyte DNA was not modified by any of the supplements, except β-carotene, which had an opposite effect in smokers and nonsmokers, with 8-OHdG increasing by 36% in smokers and decreasing by 57% in nonsmokers during the intervention. This difference in the effect of β-carotene on DNA oxidation by smoking status was statistically significant.

Mullner et al. [135] tested the hypothesis that increased consumption of fruits and vegetables, together with more PUFA intake, reduces DNA oxidation. After 8 wk of this intervention there was no significant difference in urinary 8-oxodG between the treatment and control groups.

Further details of these additional 10 DNA oxidation studies are provided in Supplemental Table 7, and results are illustrated graphically in Figure 7 [B].

### Telomere Biomarkers

Telomeres are TTAGGG repeats at the ends of chromosomes that are essential to forming the T-loop structure that prevents telomere-end fusions, which would cause genomic catastrophe in a cell and its progeny. Telomeres shorten naturally after each round of DNA replication or accidentally when breaks are caused within the telomeric DNA by oxidative stress or other factors that cause DNA strand breaks. Maintenance of telomere length (TL) is dependent on the activity of the enzyme telomerase. Nine Level 1 or Level 2 studies with quality scores 5 to 7 have been published, which reported on the effects of nutritional intervention, on its own or in combination with other lifestyle factors, on either TL and/or telomerase activity and/or oxidative damage to telomeric DNA. These studies are described briefly below.

The first in the series was by Ornish et al. [143], who performed a longitudinal intervention to test whether intensive nutrition and holistic lifestyle changes (including increased physical activity, stress management, and social support) for 3 mo improves telomerase activity in cells of the immune system (peripheral blood mononuclear cells [PBMCs]). Participants were required to consume a plant-based diet high in fruits, vegetables, unrefined grains, and legumes and low in fat (approximately 10% of calories) and refined carbohydrates. The diet was also supplemented with soy products, fish oil, vitamin E, selenium, and vitamin C. The intervention was performed in 30 men with low-risk prostate cancer. The PBMCs from blood samples collected after the intervention showed a significant 29% increase in telomerase activity (measured by TRAP assay) relative to baseline.

Ornish et al. [144] then extended their original study (described above) to follow up their original cohort for 5 y, together with a concurrent control group of low-risk prostate cancer men who were recruited using the same selection criteria. The control group did not receive the 3-mo nutrition and lifestyle intervention, and the original intervention group was provided sustained but limited guidance to help maintain their improved lifestyle after the initial 3-mo intervention was completed (see above, Ornish et al., 2008 [143]). Ten participants from the intervention group and 25 from the control group completed the full 5-y intervention with sufficient blood samples to measure all biomarkers in the study, as well as telomerase activity and TL (using qPCR assay). TL in PBMCs after 5 y increased by 9.5% in the treatment group (relative to baseline), which was a significant improvement compared with the 4.2% TL decline in the control group (relative to baseline). Furthermore, the degree of adherence to the diet and lifestyle factors in the intervention regime correlated positively with TL. Telomerase activity in PBMCs, however, decreased in both groups and although the decline was less in the intervention group, it did not achieve statistical significance compared with control.

In their study, Toupchian et al. [145, 146] investigated whether supplementation of type 2 diabetes (T2D) subjects with 2.4 g/d of the omega-3 fatty acid docosahexaenoic acid (DHA) affects telomerase activity and modifies the rate of cellular senescence. They performed an 8-wk randomized double-blind placebo-controlled intervention involving 72 T2D patients. Telomerase activity was reduced by 32% in the DHA group, with no change in the placebo group. P16 expression increased 1.86-fold in the DHA treatment group but not in the placebo group. The authors suggested that their results support a hypothesis that DHA promotes senescence by inhibiting telomerase and increasing P16 expression.

The association of obesity and vitamin D deficiency with reduced TL in cross-sectional studies prompted the study reported by Zhu et al. (2012) [147]. Overweight African Americans were recruited and assigned randomly either to placebo (*n =* 19) or the vitamin D supplementation group [oral 60,000 IU/mo, equivalent to 2000 IU/d (*n =* 18)]. Telomerase activity (TRAP assay) increased significantly in the treatment group by 19.2%, but there was no change in the placebo group.

Kiecolt-Glaser et al. [148] performed a dose-response study of the effect of supplementation with n-3 fatty acids on TL. The 16-wk intervention was performed with 106 overweight healthy sedentary-style middle-aged subjects. The design was an RCT with participants assigned randomly to one of 3 groups: (i) placebo, (ii) 1.25 g/d n-3 fatty acids, and (iii) 2.5 g/d n-3 fatty acids. After 4 mo of intervention, plasma total n-3 fatty acids increased by 58% in the 1.25 g/d group and by 110% in the 2.5 g/d group. TL only changed marginally as a result of the intervention, with a 21 and 50-base pair increase in the low and high-dose treatment groups, respectively, and a decrease of 43 base pairs in the placebo group. These changes were not statistically significant. Balcerczyk et al. [149] performed a prospective intervention study with 66 healthy women aged 35 to 55 y undertaking a 4-wk washout followed by 12 wk of intervention with a multinutrient supplement consisting of ω-3 fatty acids; ubiquinone; astaxanthin; lycopene; lutein palmitate; zeaxanthin palmitate; L-selenomethionine; cholecalciferol; and α-tocopherol. The intervention resulted in a 34% increase in telomerase in PBMCs, but TL remained unchanged.

Barden et al. [150] tested the hypothesis that supplementation with n-3 fatty acids and/or co-enzyme Q10 (CoQ) prevents telomere attrition in patients with chronic kidney disease (CKD). Eighty-five men and women with CKD participated in an 8-wk double-blind RCT in which they were randomized into 4 groups to receive one of the following treatments: (i) n-3 fatty acids, (ii) CoQ, (iii) n-3 fatty acids + CoQ or (iv) olive oil control. No significant main or interactive effects of treatment on TL measured in PBMCs or neutrophils were evident, unless corrected for neutrophil count.

Salvador et al. [151] investigated the capacity of a commercially available TA-65 supplement containing a phytochemical that activates telomerase. The patented active ingredient was discovered in Astragalus during a phytochemical compound screen of plants used in traditional Chinese medicine. Salvador et al. performed an RCT to test whether supplementation with TA-65 improved the TL of apparently healthy adults infected with cytomegalovirus. A total of 117 participants were recruited, and 97 completed the 12-mo RCT. Participants were randomly assigned to one of 3 groups, consuming daily either (i) placebo (*N =* 52), (ii) 250 units of TA-65 (*N =* 22), or (iii) 1000 units of TA-65 (*N =* 23). After 12 mo of intervention, TL in the placebo group declined by 3%, TL in the TA-65 250 units group increased by 5%, and there was no significant change in the TA-65 1000 units group.

Zinc plays an important role in DNA metabolism as a cofactor of DNA polymerases and as an essential cofactor or structural component for antioxidant defense proteins and DNA repair enzymes [152]. Sharif et al. (88) performed a double-blind RCT to test whether zinc (as zinc carnosine chelate) supplementation for 12 wk improved genomic stability and TL in healthy older adults with low plasma zinc status. The 84 participants were randomly assigned to 2 groups; either placebo (*n =* 42) or zinc supplemented (*n =* 42). Both groups showed an increase in TL (measured by qPCR) after 12 wk of intervention, with an increment of 9.7% for the zinc supplemented group and 10.4% for the placebo group relative to baseline, but between-group comparisons were not significant, suggesting no effect of zinc supplementation on TL relative to control. Telomere base damage (measured by qPCR after FPG digestion) was found to be signiﬁcantly decreased by 51% in the zinc group and also decreased by 32% in the placebo group. The between-group comparison was not statistically significant, suggesting no substantial effect of zinc supplementation on telomere base damage relative to control.

Further details of these 9 telomere biomarker studies are provided in Supplemental Table 8, and the results are illustrated graphically in Figure 8.

### Mitochondrial DNA Biomarkers

The proper function of mitochondria is critical for normal cell function because of the numerous metabolic processes they perform, such as ATP generation and synthesis of specific metabolic forms of folate. There is emerging evidence that mutations in the mitochondrial genome are linked with neurodegenerative diseases and may be caused by the high-level of oxidative stress within the mitochondria and/or by defective replication of the mitochondrial genome, either because of accumulation of unrepaired base lesions or DNA strand breaks or because of deficiency of micronutrients required for DNA synthesis [6]. For these reasons, there has been considerable interest in determining the nutritional causes of mitochondrial DNA (mtDNA) mutations, including mtDNA copy number. Despite this interest and the criticality of having such knowledge, we could find only one intervention study of sufficient quality to provide a report on its outcome.

In 2004 Wang et al. reported that a traditional Chinese medication known as Wuzi Yanzong Granule (WYG) improved cognitive function in mild cognitive impairment (MCI) patients [153]. They followed up this study by performing a second double-blind placebo-controlled intervention of 13 wk duration to determine effects on biomarkers indicative of oxidative stress, amyloid status, and damage to mitochondrial DNA and test whether the changes in these biomarkers were associated with improvements in cognitive function [154]. Thirty-six MCI patients were recruited for the intervention and randomly assigned to consume either a placebo or WYG. The MCI group treated with WYG showed a significant 31% decrease in the mtDNA deletion rate. But the mtDNA deletion rate in the placebo group did not change during the intervention. MCI patients consuming WYG also showed improved memory function, decreased serum β-amyloid, and reduced malondialdehyde (a biomarker of lipid peroxidation).

## Discussion

This review is the first to systematically analyze current knowledge on the effect of vitamin, mineral, phytochemical, or plant food supplementation on DNA damage in humans using the best-validated biomarkers that are currently available. The majority of the 134 interventions we reviewed (illustrated in FIGURE 3, FIGURE 4, FIGURE 5, FIGURE 6, FIGURE 7, FIGURE 8) showed a significant reduction in DNA damage (*n =* 86), some showed no significant effect (*n =* 43), and a few (*n =* 5) indicated an adverse outcome. The high level of positive outcomes (64.2%) is not unexpected because the justification for performing a human intervention is often based on preclinical data showing genome-protective effects. Null effects could have been due to a lack of deficiency of the micronutrients being tested in the cohorts that were investigated. Adverse outcomes observed in adults consuming blackcurrant juice and in adult smokers supplementing with β-carotene may be explainable by an excess of vitamin C or a synergistic pro-oxidant effect of blackcurrant polyphenols that chelate iron ions in the presence of vitamin C in the former study [128] and enhancement of inflammation-induced oxidative DNA damage by beta-carotene metabolites in smokers in the latter study [155]. A plausible explanation for the increase in micronuclei in lymphocytes and buccal cells in children with ulcerative colitis receiving high doses of folic acid supplements remains lacking [85].

The outcomes of these interventions verify the plausibility of reducing DNA damage levels in humans nutritionally. Furthermore, they support the concept that DRV for DNA damage prevention of micronutrients could be further improved by using the DNA damage biomarkers used in the interventions we reviewed. However, it is evident, despite the relatively high-quality scores of the selected studies, that there is considerable heterogeneity in some important aspects such as (i) age, gender, and health status of the cohort studied, (ii) the number of nutrient doses investigated, (iii) the duration of the intervention, (iv) information about background diet, (v) selection of participants depending on whether they are deficient or replete for the micronutrient being investigated, and (vi) the DNA damage status of the selected participants.

Above-average DNA damage levels in members of a cohort could be the consequence of multiple factors that may interact with each other to cause an increase in the DNA damage biomarker being investigated. Such factors may include genetic defects in antioxidant and DNA repair enzymes and/or high exposure to lifestyle, occupational, endogenous, or environmental genotoxins, in combination with a deficiency in micronutrients required as cofactors for DNA replication, DNA repair, and function of cellular mitotic machinery. In a practical sense, the utility of nutrient supplementation for DNA damage prevention may be mainly relevant to those with above-average levels of DNA damage and/or deficiency in the supplementation nutrient/s.

**FIGURE 9:**
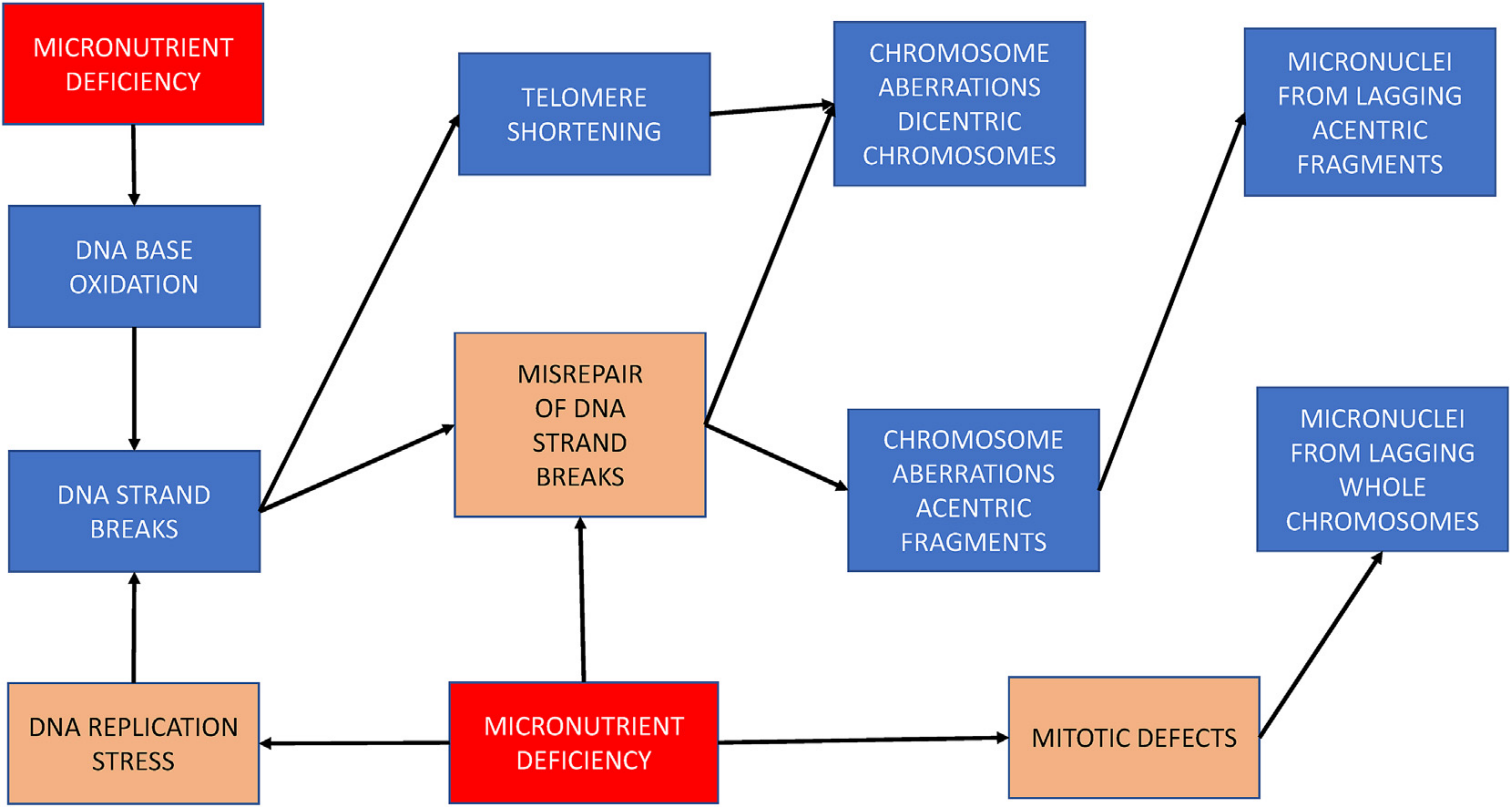
Mechanistic inter-relationship between effects of micronutrient deficiency and DNA damage biomarkers and also between the DNA damage biomarkers used in the reviewed studies. (Red indicates micronutrient deficiency; Blue indicates DNA damage biomarkers; Orange indicates DNA integrity pathway affected)

It is generally accepted that more than one biomarker is required to measure changes in genome integrity at both the molecular level (e.g., DSBs, DNA oxidation, TL) and cytogenetic level (chromosome aberrations, micronuclei); the mechanistic inter-relationship between these biomarkers and nutritional deficiency is summarized in Figure 9. These biomarkers are complementary to each other because DNA oxidation can lead to DNA breaks, which in turn results in terminal deletions, leading to telomere loss and formation of acentric chromosome fragments. The latter can then result in micronuclei formation because acentric chromosome fragments cannot engage with the mitotic spindle and, thus, are excluded from the main nuclei during nuclear division. Micronuclei also occur with failure of segregation of whole chromosomes due to damage to the centromeres, kinetochores, and mitotic proteins [156]. Ideally, the DNA damage biomarkers should be measurable in both hematopoietic cells (such as lymphocytes) and epithelial cells (such as buccal cells) that can be collected in a minimally invasive manner. A recent series of meta-analyses and prospective studies verified that increased micronucleus frequency in both lymphocytes and buccal cells is associated with an increased risk of a wide range of cancers and noncancer diseases [156]. In this regard, the strong and consistent effects of vitamin A/beta-carotene in reducing risk of MN in buccal cells of subjects in different countries exposed habitually to lifestyle carcinogens (betel nut, tobacco) is notable [[96], [97], [98], [99], [100]]. Evidence replicated in more than one laboratory using well-designed micronutrient interventions that can reproducibly improve genome integrity, measured by more than one biomarker, in both lymphocytes and buccal cells, would increase confidence in the DNA prevention effects and safety of a food or supplement. Consistency of nutrient supplementation effects across complementary DNA damage biomarkers also increases confidence in observed genome integrity effects. The relative differences between biomarkers may also inform the mechanisms by which a micronutrient may cause a change in genomic stability. The matrix of biomarker data relative to vitamin, mineral, phytonutrient, and plant food supplementation from the reviewed studies (Supplemental Tables 10 A and 10 B) suggests varying levels of consistency across biomarkers; however, this may be due to differences between studies with regards to the cohort studied, duration of intervention and the doses used. Most studies investigated only one biomarker or a combination of 2 biomarkers, such as MN in lymphocytes and in buccal cells, chromosome aberrations and micronuclei in lymphocytes only, DNA strand breaks, and DNA oxidation only.

To establish nutrient reference values for DNA damage prevention, it is essential to not only perform dose-response studies but also that such studies are performed in both those who have low intakes and those having high consumption levels of the micronutrient under investigation. This approach is optimal to test which group benefits from the supplementation and at which doses supplementation may either decrease or increase DNA damage, given that it is quite common to observe U-shaped relationships between dosages of a micronutrient and biomarkers of DNA damage [88, 157, 158]. By far, most studies we reviewed only investigated single doses and did not screen participants for their micronutrient status or control their background diet. In fact, we could only find one study that included both dietary control in a metabolic unit and different doses of the micronutrient (folic acid) using a depletion and repletion study design [84]. We suggest that future studies should incorporate these features in randomized placebo-controlled trials whenever possible so that both the deficiency state, replete, and beyond replete levels can be achieved and evaluated for their bioefficacy and safety.

Very few of the studies we reviewed compared the effect of single micronutrients relative to their combination within the same intervention. Comparing within or across studies, it was not uncommon to observe that single micronutrients, including phytochemicals, sometimes had protective effects that were of a similar magnitude to that of combinations suggesting that the additional nutrients in a combination did not appear to confer extra genome-protective effects; however, in other studies, the combination appeared to be more beneficial than a single micronutrient. Supplemental Figure 1 shows a comparison of results for studies relating to single vitamins or combinations of vitamins on either lymphocyte micronuclei or buccal cell micronuclei. The trends in these results suggest that depending on the cell type, DNA damage prevention effects may be more pronounced in buccal cells rather than lymphocytes and/or if a combination of micronutrients is used rather than only a single micronutrient. These hypotheses need to be tested in further studies that use both lymphocytes and buccal cells, and that also compare the effects of single micronutrients relative to their combination. Also, it may be beneficial to investigate the combination of selected micronutrients and phytochemicals based on their suggested mechanism, which may be complementary.

One of the important knowledge gaps in this field is the optimal duration of intervention. This may vary depending on the micronutrient/s metabolism, the mitotic status of the cells that are being sampled, and the extent to which the selected DNA damage biomarkers are dependent on mitosis for their expression. Ideally, the supplementation regime should be chronic (e.g., on a daily basis) and the intervention duration long enough to enable a steady state to be achieved between improvement of DNA integrity in progenitor cells (e.g., basal cells in the case of buccal cells, and stem cells in the bone marrow, spleen, and lymph nodes in the case of peripheral white blood cells) and the differentiated cells in which the DNA damage biomarkers are measured. We chose 21 d as the optimal minimum time as one of the parameters in the QS for study selection because previous studies showed that 14 to 21 d is an adequate time for buccal cells to optimally express micronuclei in genotoxin exposure studies [[159], [160], [161]]. In contrast, lymphocytes are generated from dividing stem cells in the bone marrow, a proportion of which leave the bone marrow and circulate in the bloodstream until they mature as T-lymphocytes in the thymus. They then relocate to the lymph nodes, where they become specialized for adaptive immune function to recognize foreign antigens and eliminate pathogenic viruses, infected cells, and aberrant cells such as cancer cells [10, 65, 162, 163]. Micronuclei in lymphocytes may be generated in vivo during mitosis in the bone marrow and other sites of lymphopoiesis or ex vivo in tissue culture after stimulation to divide using a mitogen. The most commonly used method to score micronuclei in lymphocytes is the cytokinesis-block method, in which once-divided cells are recognized by their binucleated appearance after blocking cytokinesis, with micronuclei specifically scored in these once-divided cells. The kinetics of micronucleus formation in dividing progenitor cells of lymphocytes in the bone marrow in humans and the rate at which nascent lymphocytes, with or without micronuclei, leave the bone marrow and enter the bloodstream is unknown. However, it is known that induction of DNA strand breaks by ionizing radiation is instantaneous, and if left unrepaired or misrepaired, results in acentric chromosome formation from which micronuclei are generated after mitogen stimulation ex vivo [164, 165]. Therefore, induction of DNA strand breaks in lymphocytes due to increased oxidative stress in vivo is a likely cause of MN in lymphocytes, expressed in vivo or ex vivo. The underlying oxidative stress may be a consequence of a deficiency in dietary antioxidants, and the extent of the resulting DNA damage may be aggravated by inadequate intake of cofactors required by DNA polymerases or DNA repair proteins or due to DNA replication stress. For example, folate and/or vitamin B-12 deficiency can result in failure to synthesize nucleotides (such as thymidine), resulting in DNA replication stress and DNA strand break formation [166, 167].

Another relevant question relates to what level of reduction in DNA damage is biologically meaningful with respect to disease prevention. In vitro, studies with lymphocytes showed that 12 nM folate concentration induces MN frequency to a level equivalent to 20 rad of X-rays, which increases cancer risk [168]. Furthermore, it was shown that increasing folic acid concentration (in the physiological range) from 20 nM to 120 nM reduces MN frequency by 74% [33, 168]. Using the rad-equivalent concept, it is possible to establish a biologically plausible standard by which to establish the health significance of specific quantitative reductions in DNA damage biomarkers. Another approach is to utilize recent data that was meta-analyzed on the fold-increase in micronucleus frequency in disease versus healthy controls across a wide range of cancers and noncancer diseases [156].

In this review, we chose to include intervention studies in children and in those with a medical condition because results from such studies may provide insights into whether the effects of micronutrient supplementation may vary depending on age or disease status. For example, it is notable that the study of high-dose folic acid supplementation in children by Holland et al. [85] showed no effect in healthy children, a reduction of MN in children with Crohn’s disease, and a strong increase in MN frequency in ulcerative colitis patients. These findings highlight the importance of intestinal inflammatory status as a determinant of DNA damage outcomes. Another example is benfotiamine, a prodrug of vitamin B1, which reduces advanced glycation end products (AGEs) and is shown to reduce MN in hemodialysis patients, a cohort often presenting with high levels of AGEs [78]. The possibility of reducing DNA damage and aneuploidy, both major factors in the pathologies of aging, by vitamin B1 supplementation presents a novel aspect of great relevance given the heavy disease burden caused by diabetes. Future studies should explore the burgeoning possibilities of more targeted approaches to optimize DNA damage prevention both in apparently healthy subjects and those with chronic conditions (including diabetes and cancer), given that responses to micronutrient supplementation in such cases may be different from that of healthy subjects.

Despite the great interest in telomeres and their association with diseases of aging, only a small number of micronutrient interventions have been reported relative to the other DNA damage biomarkers. The question of what degree of telomere shortening is required to have pathological (clinical) consequences and whether there is any benefit in the elongation of already sufficiently long telomeres remains controversial. Damage occurring within telomeric DNA sequences (oxidized guanine, DNA breaks) is not repaired by the cell and instead triggers the proinflammatory SASP, further aggravating the aging process [169]. Further complicating this aspect, whereas telomere lengthening in peripheral blood cells is clearly associated with reduced risk of cardiovascular disease, longer telomeres have been associated with increased risk of certain cancers such as lung cancer, particularly in men [25, 170]. Perhaps a less equivocal approach is to use methods that measure telomere loss due to terminal chromosome deletions, as this has clear pathogenic consequences. Terminal chromosome deletions result in the formation of chromosome end fusions, leading to dicentric chromosome formation and hypermutation due to breakage-fusion-bridge cycles. The latter generate aberrant karyotypes and abnormal cells in the body [171].

Several studies suggest that subclinical deficiency of micronutrients required for the maintenance of genome integrity could be prevalent because of episodic or chronic inadequate intake [58]. However, DNA damage is not fatal, and the elevated risk of cancer and other chronic diseases it causes with aging may be tolerated by natural selection because the effect on reproduction is only evident if the levels of DNA damage are excessive [41]. According to the Triage Theory proposed by Bruce Ames [172], a scarcity of micronutrients required for vital functions such as ATP production that are also required for DNA metabolism (e.g., zinc, B vitamins) may be prioritized to the former at the expense of the latter, further aggravating cellular capacity for reproduction and DNA damage prevention. For these reasons, it is relevant to explore more carefully and systematically the nutritional requirements for the maintenance of genome integrity.

This review on nutritional intervention studies to prevent DNA damage is novel in that it has established criteria for (i) identifying the best-validated DNA damage biomarkers in humans based on the level of evidence of their association with health and disease, (ii) determining which human dietary intervention studies used these biomarkers, and (iii) using a novel QS tool based on 7 criteria relating to study design to generate a shortlist of papers reporting the best-designed dietary intervention studies utilizing the best-validated DNA damage biomarkers. The review is also novel because it is the first to investigate all of the leading papers reporting on the effects of human nutritional interventions across all of the best-validated DNA damage biomarkers that have been published until 2017.

The main strength of this review is that it has brought together in a systematic manner the wealth of knowledge that has been accumulated over many decades on the effect of a wide range of nutritional supplementation on genome integrity in humans. Furthermore, the comprehensiveness of this milestone review and its outcomes provides a solid foundation for the eventual establishment of DRV for DNA damage prevention. However, the review also presents some weaknesses. For example, some of the criteria in the QS, such as the number of subjects and duration of the intervention (>10 subjects per group, > 21 d, respectively), were chosen based mainly on experience with study designs that are adequate to observe statistically significant changes in DNA damage biomarkers in human intervention studies. However, whether these particular parameters are optimal in selecting the most reliable studies is unknown. We also included the significance of the *P* value as one of the criteria for the QS because, in our view, it could be an indicator of whether the study was adequately powered to detect an increase or decrease in DNA damage given that results in either direction are relevant when establishing DRV. This unconventional criterion may be questioned because it might disfavor the inclusion of studies with null results from the shortlist of papers for review, but, in fact, we found that the *P* value criterion did not affect the distribution of papers (based on no change, increase or decrease in DNA damage) within the group that was shortlisted for review compared with the excluded group.

The QS we introduced in this study deserves some further discussion with regards to using the *P* value as one of the QS criteria. The reason for deducting a point in the QS of a study if the differences in results between groups were not statistically significant is that the study may not have been adequately powered to detect a small difference in DNA damage. Although the deduction of a point in the QS due to nonsignificant results may increase the probability of exclusion from the QS > 5 category, this may be mitigated by good scores for the other 6 criteria in the QS. In fact, the percentage of studies reporting no statistical difference in DNA damage between the control and treatment groups was 21% and 29% in the QS < 5 and the QS > 5 groups, respectively. Furthermore, the percentage of studies reporting decreases in DNA damage was 75% in the QS < 5 and 65% in the QS > 5 groups. Analysis of this data showed no significant difference in the distribution of the studies between the QS < 5 and QS > 5 groups when tested by the Chi Square method. This indicates that the inclusion of the *P* value criterion in QS did not discriminate against those studies reporting results that were not statistically different.

Another limitation of our review is that we did not include the most recent studies published from January 2018 to March 2023. We identified 15 human interventions that investigated the effect of nutrient supplements (9 studies) or dietary change (6 studies) in this period [[173], [174], [175], [176], [177], [178], [179], [180], [181], [182], [183], [184], [185], [186], [187]]. The proportion of studies on effects induced by dietary change using whole foods (40%) or nutrient supplements (60%) suggests the continued interest in investigating the genome-protective effects of whole foods or supplements, but the heterogeneity in study design with respect to the health status of subjects participating in the interventions, duration of interventions and biomarkers used remains evident. Three of the nutrient supplementation studies were focused on male infertility, which appears to be an area of continuing interest in this field.

## Conclusion

This review provides clear evidence that it is feasible to substantially reduce DNA damage levels in humans by intervention with a wide range of vitamins, minerals, phytochemicals, whole plant juices, and foods. Future studies should improve on the investigations done so far by using study designs that (a) carefully select subjects that are more likely to benefit from micronutrient interventions for DNA damage prevention based on their nutritional and genome integrity status, (b) minimize confounding effects of other variables affecting DNA damage, such as a more stringent control of background diet and lifestyle habits, (c) use intervention durations and sampling times that can best capture the maximum effect of the intervention until a plateau in DNA damage change is achieved, (d) investigate multiple doses of supplementation to identify the most efficacious dosage and those high doses that may confer no benefit, (e) replicate a study in another cohort. Ultimately, long-term studies are needed to test whether DNA damage reduction by means of nutritional supplementation does lead to improved health outcomes across the lifespan, both in those who are chronically undernourished and/or those with a genetic predisposition to elevated loss of genome integrity.

## Author Contributions

The authors’ responsibilities were as follows—MFF, BJWvK, and CFB designed the research; MFF and CFB conducted the research and analyzed the data: MFF, CFB, and BJWvK wrote the paper; MFF and CFB had primary responsibility for the final content. All authors read and approved the final manuscript.

## Conflict of interest

MFF and CFB declare no conflict of interest. BJWvK was an employee of GSK/Pfizer Consumer Healthcare, now known as Haleon.

## Funding

MFF and CFB were funded by Haleon (formerly Pfizer/GSK Consumer Healthcare) to undertake the literature review. CSIRO generously supported Maryam Hor’s contribution to assist in preparing the manuscript for publication. BJWvK was an employee of GSK/Pfizer Consumer Healthcare (now known as Haleon).

## Data Availability

Data described in the manuscript will be made available upon request.

## Appendix A Supplementary data

Multimedia component1 Multimedia component1

Multimedia component2 Multimedia component2
