# Supplementary material for: Protective Effects of Micronutrient Supplements, Phytochemicals and Phytochemical-Rich Beverages and Foods Against DNA Damage in Humans: A Systematic Review of Randomized Controlled Trials and Prospective Studies
Source: Adv Nutr. 2023 Aug 18;14(6):1337–58. doi: 10.1016/j.advnut.2023.08.004 (PMC10721466; doi:10.1016/j.advnut.2023.08.004)
Supplement: Multimedia component1 [file mmc1.pptx]

## Slide 1
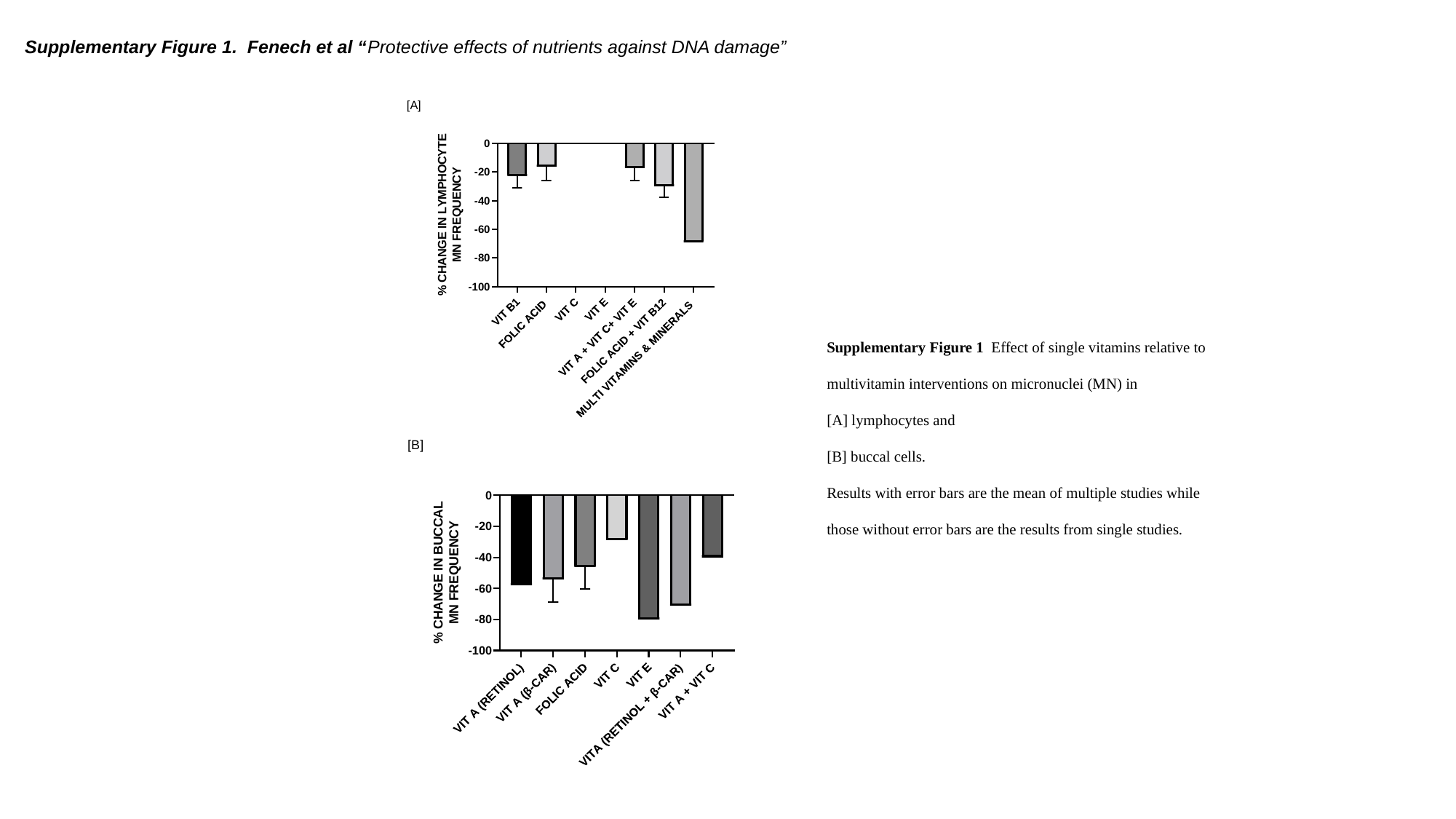

Supplementary Figure 1. Fenech et al “Protective effects of nutrients against DNA damage”
Supplementary Figure 1 Effect of single vitamins relative to multivitamin interventions on micronuclei (MN) in
[A] lymphocytes and
[B] buccal cells.
Results with error bars are the mean of multiple studies while those without error bars are the results from single studies.
