# Supplementary material for: Protective Effects of Micronutrient Supplements, Phytochemicals and Phytochemical-Rich Beverages and Foods Against DNA Damage in Humans: A Systematic Review of Randomized Controlled Trials and Prospective Studies
Source: Adv Nutr. 2023 Aug 18;14(6):1337–58. doi: 10.1016/j.advnut.2023.08.004 (PMC10721466; doi:10.1016/j.advnut.2023.08.004)
Supplement: Multimedia component2 [file mmc2.docx]

***Fenech et al “****Protective effects of nutrients against DNA damage”*

**List of Supplementary Tables**

| **Supp Table #** | **Title** | **Page** |
| --- | --- | --- |
| Supp Table 1 | Search strings used for systematic literature search | 2 |
| Supp Table 2 | Levels of evidence for studies examining the association between micronutrients and reduction in DNA damage | 4 |
| Supp Table 3 | Parameters used to determine quality scores for Level 1 and Level 2 human intervention studies | 5 |
| Supp Table 4 | Chromosome Aberrations | 6 |
| Supp Table 5A | Micronuclei (MN) measured in lymphocytes using the CBMNcyt assay | 11 |
| Supp Table 5B | Micronuclei (MN) in buccal cells | 30 |
| Supp Table 6 | DNA Strand breaks, determined with the Comet assay | 35 |
| Supp Table 7 | DNA Oxidation | 57 |
| Supp Table 8 | Telomere length / Telomerase | 63 |
| Supp Table 9 | Mitochondrial DNA mutations / copy number | 69 |
| Supp Table 10A | Matrix summary of % change in levels of DNA damage biomarkers  for interventions with vitamins, minerals and their combinations | 70 |
| Supp Table 10B | Matrix summary of % change in levels of DNA damage biomarkers  for interventions with phytonutrients, whole plant foods or juices, and  plant-based diets | 72 |

**Supplementary Table 1**. Search strings used for systematic literature search.

| **Search #** | **Topic** | **Search string** |
| --- | --- | --- |
| 1 | Chromosomal aberrations | Chromosom* (aberration* OR abnormalit*) AND (leukocyte OR lymphocyte OR buccal) AND nutrition term (nutrition/nutrient*/phytochem* OR phytonutrient*/mineral*/radioprotect* /antioxidant*/vitamin*/mineral*/amino acid*/fatty acid*/malnutrition/protein malnutrition) |
| 2 | Micronuclei | Micronuc* AND (leukocyte OR lymphocyte OR buccal) AND nutrition term (nutrition/nutrient*/phytochem* OR phytonutrient*/mineral*/radioprotect* /antioxidant*/vitamin*/mineral*/amino acid*/fatty acid*/malnutrition/protein malnutrition) |
| 3 | Telomeres | Telomer* AND (leukocyte OR lymphocyte OR buccal) AND nutrition term (nutrition/nutrient*/phytochem* OR phytonutrient*/mineral*/radioprotect* /antioxidant*/vitamin*/mineral*/amino acid*/fatty acid*/malnutrition/protein malnutrition) |
| 4 | Methylation | DNA methyl* AND (leukocyte OR lymphocyte OR buccal) AND nutrition term (nutrition/nutrient*/phytochem* OR phytonutrient*/mineral*/radioprotect* /antioxidant*/vitamin*/mineral*/amino acid*/fatty acid*/malnutrition/protein malnutrition) |
| 5 | DSB | (Double strand break* OR DSB OR Comet assay) AND (leukocyte OR lymphocyte OR buccal) AND nutrition term (nutrition/nutrient*/phytochem* OR phytonutrient*/mineral*/radioprotect* /antioxidant*/vitamin*/mineral*/amino acid*/fatty acid*/malnutrition/protein malnutrition) |
| 6 | DNA oxidation | DNA oxidat* AND (leukocyte OR lymphocyte OR buccal) AND nutrition term (nutrition/nutrient*/phytochem* OR phytonutrient*/mineral*/radioprotect* /antioxidant*/vitamin*/mineral*/amino acid*/fatty acid*/malnutrition/protein malnutrition) |
| 7 | Mitochondrial DNA | Mitochond* DNA AND (leukocyte OR lymphocyte OR buccal) AND nutrition term (nutrition/nutrient*/phytochem* OR phytonutrient*/mineral*/radioprotect* /antioxidant*/vitamin*/mineral*/amino acid*/fatty acid*/malnutrition/protein malnutrition) |

DSB, DNA strand break;
The asterisk (*) has been employed as a wildcard to capture all permutations of the base word.

**Supplementary Table 2.** Levels of evidence for studies examining the association between micronutrients and reduction in DNA damage

| Evidence level | Study design |
| --- | --- |
| Level 1 | Randomized, placebo-controlled intervention study (including parallel or crossover-design studies) |
| Level 2 | Longitudinal intervention study (without untreated matched control; including repletion/depletion studies) |
| Level 3 | Cross-sectional study with blood nutrient data |
| Level 4 | Cross-sectional study with dietary intake data |
| Level 5 | *In vitro* studies using human cells |
| Level 6 | *In vitro* and *in vivo* studies in mammalian models |

**Supplementary Table 3.** Parameters used to determine quality scores for Level 1 and Level 2 human intervention studies

| Parameter | 0 Points | 1 Point |
| --- | --- | --- |
| Evidence level | Level 2 study | Level 1 study |
| Number of subjects per group | <10 per group | >10 per group |
| Duration of intervention | <21 days | ≥21 days |
| Placebo | No | Yes |
| Age Balance | Not well matched for age  (significant difference between groups) | Well matched for age (no significant difference) |
| Gender Balance | Not well matched for gender  (significant difference between groups) | Well matched for gender  (no significant difference) |
| Statistical significance of main outcome measure | *P* value for main outcome measure not significant | *P* value for main outcome measure significant |
| Maximum Possible Quality Score: 7 | | |

**Supplementary Table 4: Chromosome Aberrations**

| Reference | Micro-nutrient  class | Nutrient/s tested | Dose/s tested | Type and duration of intervention | Health status, age, gender, and number of subjects | Cell type | Change in micronutrient concentration in blood  (% change) | Effect on Biomarker/s | QUALITY SCORE  (0-7) |
| --- | --- | --- | --- | --- | --- | --- | --- | --- | --- |
| Li N et al (1999) | Phytonutrient | Mixed tea product. Dried mixture of whole water extract of green tea, green tea polyphenols, and tea pigments in 4:1:1 mixture. | 3g mixed tea product powder (in capsules) per day. | Placebo-controlled intervention.  Duration:  24 weeks | 64 cases of oral leukoplakias,  Age: 23-28 years  40 males,  24 females | Lymphocyte | Tea polyphenols were not measured in blood | 30% decrease in chromosome aberrations in the tea-treated group (P<0.01) | 7 |
| Dusinska M et al (2003) | Vitamin, Mineral | vitamin C, vitamin E, β-carotene, selenium,  in combination | vitamin C (100mg/day), vitamin E (100mg/day), β-carotene (6mg/day), selenium (50μg/day), in combination | Placebo-controlled intervention;  Duration:  12 weeks | 28 myocardial infarction survivors and  57 rural healthy controls  Age: 35-66 years | Lymphocyte | 50% increase in vitamin C.  50% increase in vitamin E.  80% increase in β-carotene.  40% increase in Se | No effect in myocardial infarction survivors.  60% decrease in chromosome aberrations in rural healthy controls (P=0.03) | 6 |
| Anderson D. et al (1997) | Vitamin | Vitamin C | 60mg vitamin C per day (recommended daily allowance)  6000mg vitamin C per day | Placebo-controlled intervention with cross-over design.  Duration: 2 weeks | Healthy non-smoking males and females  Age: 20-68 years, 24 males,  24 females | Lymphocyte | 20% and 80% increase in plasma vitamin C after consumption of 60mg or 6000mg per day vitamin C | No effect on spontaneous chromosome aberrations. High dose vitamin C (6000mg/day) increased in vitro sensitivity to bleomycin-induced DNA damage | 5 |
| Joksic G et al (2009) | Phytonutrient | *Echinacea purpurea* | Two 275mg *Echinacea purpurea* tablets (Echinacea, Strong Nature, Serbia) twice a day. | Longitudinal intervention without placebo control.  Duration: 2 weeks  Blood samples collected before and after | Radiology technicians identified as carrying dicentric chromosomes  Average age : 38.7 years  10 subjects | Lymphocyte | *Echinacea purpurea* polyphenols were not measured in blood. | 70% decrease in chromosome aberrations per cell P = 0.004 | 5 |
| Nakamura et al. 2017 | Phyto-nutrient | Tomato juice | Daily consumption of 190g of tomato juice containing 17mg lycopene and 0.25mg β-carotene. | Longitudinal intervention without placebo group.  Duration:  3 weeks of daily intake of tomato juice followed by 3 weeks refraining from it. | Healthy subjects  Average age: 23 years  5 males,  5 females | Lymphocytes | Plasma β-carotene and lycopene increased by 26% and 50% respectively after tomato juice consumption phase. | Base-line dicentric chromosome frequency was not altered but dicentric chromosomes induced by 0.5Gy ionising radiation (X-rays) were reduced by 13% (P < 0.05) after tomato juice consumption and increased after the 3 week washout. | 5 |
| Rompelberg CJM et al (1996 | Phytonutrient | Eugenol (main component of oil of cloves) | 150mg Eugenol Per day | Placebo-controlled intervention with cross-over design. Duration: 1 week | Healthy non-smoking subjects  Age: 21-26 years, 10 males | Lymphocytes | 24 hour urines of all participants on 150mg Eugenol per day were positive for Eugenol. Results were negative in controls. | No effect on spontaneous or mutagen-induced chromosome aberrations | 5 |
| Sram RJ et al (1983a) | Vitamin | Vitamin C  (ascorbic acid) | 1.0g vitamin C per day, 5 days per week for 3 months | Longitudinal intervention without placebo control.  Duration:  12 weeks  Blood samples collected immediately before and after intervention | Coal-tar workers occupationally exposed to polycyclic aromatic hydrocarbons and benzene.  Average age: 41.9 years 35 males | Lymphocyte | Vitamin C was not measured in blood | 70% decrease in cells with chromosome aberrations (P<0.01) | 5 |
| Sram RJ et al (1983b) | Vitamin | Vitamin C (ascorbic acid) | 1.0g vitamin C per day,  5 days per week, for 5 months | Longitudinal intervention without placebo control.  Duration:  20 weeks  Blood samples collected immediately before and after intervention | Workers occupationally exposed to halogenated ethers.  Average age: 38.4 years  77 healthy workers; gender not specified | Lymphocyte | 300% increase in vitamin C (P<0.01) | 40% decrease in cells with chromosome aberrations (P<0.01) | 5 |
| Sidneva ES et al (2005) | Vitamin  Mineral  Phytonutrient | Vitamin mineral complex including ginkgo biloba, garlic extract, Q10 coenzyme, grape seed extract, ginseng root, guarana, echinacea, spirulina, royal jelly, Alfalfa powder, and chlorella. | The daily dose of vitamins and trace elements was as follows:  Β-carotene (3200IU), vitamin C (90mg), vitamin E (98.2IU), vitamin B1 (20mg), vitamin B5 (30mg), vitamin B6 (12mg), vitamin B12 (100μg), vitamin D (600IU), vitamin K (20μg),  Niacinamide (50μg), biotin (60μg), folic acid (800μg), chromium (13μg), calcium (300mg), copper (2mg), iodine (300μg), iron (8mg), magnesium (100mg), molybdenum (50μg), Nickel (2.5μg), Potassium (60mg), Selenium (40μg), Silicon (20μg), Vanadium (20μg), Manganese (3mg), Phosphorous (230mg), zinc (15mg). | Longitudinal intervention without placebo control.  Duration: 2 weeks,  4 weeks  Blood samples collected before and after 2 weeks, and after 4 weeks | Healthy young adults.  Average age:  32.6 years  8 females, 7 males | Lymphocyte | 60% increase in  vitamin B2.  20% increase in vitamin C.  90% increase in vitamin E.  10% increase in retinol.  520% increase in  β-carotene. | No effect on spontaneous chromosome aberrations.  20% and 10% decrease in cadium chloride-induced and dioxidine-induced chromosome aberrations in vitro, respectively (P<0.01). | 3 |

(Shading indicates study with Quality score <5)

**Supplementary Table 5A: Micronuclei (MN) measured in lymphocytes using the CBMNcyt assay**

| Reference | Micro-nutrient class | Nutrient/s tested | Dose/s tested | Type and duration of intervention | Health status, age, gender, and number of subjects | Cell type | Change in micronutrient concentration in blood  (% change) | Effect on Biomarker/s | QUALITY SCORE 0-7 |
| --- | --- | --- | --- | --- | --- | --- | --- | --- | --- |
| Fenech M et al. 1998 | Vitamin | Folic acid,  Vitamin B12 | 7μg vitamin B12 + 700μg folic acid each day,  And  20μg vitamin B12 + 2000μg folic acid each day | Randomised double-blind placebo-controlled trial.  Duration:  12 weeks on 7μg vitamin B12 + 700μg folic acid each day, or placebo  Followed by 12 weeks 20μg vitamin B12 + 2000μg folic acid each day, or placebo | Healthy young adults  Age: 18-32 years  32 females, 31 males | Lymphocyte | Serum B12 increased by 15%, red cell folate increased 307%, plasma homocysteine decreased by 32%  at the end of the intervention relative to base-line in the treatment group. There was no change in the placebo group. | There was a 15.3% reduction in MN frequency (P < 0.03) in the treated group and a 25.4% reduction (P < 0.01) for those in the treated group with above average MN frequency at base-line.  MN frequency was not altered in the placebo group. | 7 |
| Li W et al. 2000* | Mineral | Selenium | Sodium selenite. 0.5mg Se every day | Randomised placebo-controlled intervention.  Duration: 156 weeks | Subjects in Qidong, Jiangsu province in China with a high risk of liver cancer.  Age: 20-65 years  18,000 males | Lymph-ocyte | Blood selenium concentration and glutathione peroxidase activity increased significantly in the sodium selenite treatment group relative to control (P < 0.01). | Frequency of MN reduced by 31% in the sodium selenite treatment group relative to the control group (P < 0.01). | 7 |
| Schupp N et al 2008a | Vitamin | Vitamin B1 (Thiamine) | Vitamin B1 prodrug benfotiamine (milgamma-mono 150, Worwag Pharma, Germany), 600mg daily for 12 weeks. | Single-blind placebo-controlled intervention.  Duration: 12 weeks | 23 haemodialysis patients  Average age:  65.5 years  Placebo group:  8 males, 8 females  Treatment group:  9 males. 6 females | Lymphocytes | Blood thiamine concentration increased 1,800%  (18-fold) in the treatment group and declined by 31% in the placebo group  (P < 0.001) | MN frequency declined by 15% (P < 0.01) after the intervention relative to base-line in the treatment group.  There was no significant change in the placebo group. MN frequency was significantly negatively correlated (R = -0.33. P < 0.025) with transketolase activity, a metabolic biomarker of thiamine status. | 7 |
| Sharif R et al. 2015 | Mineral  Amino acid | Zinc  Carnosine | Zinc carnosine.  One zinc carnosine tablet per day. Each tablet contained 87mg of zinc carnosine of which 20mg was zinc. | Randomised placebo-controlled intervention in a free-living elderly healthy population with low zinc status.  Duration: 12 weeks | 84 healthy participants with low plasma zinc (< 11.7μM).  Age: 65-85 years | Lymphocyte | Plasma zinc concentration increased by 5.7% in the zinc-carnosine supplemented group  (P < 0.05) | A signiﬁcant 24.2 % decrease (p < 0.05) in the MN frequency was observed for the Zn supplemented cohort relative to baseline compared to the placebo group. | 7 |
| Smolkova B. et al 2004 | Vitamin  Mineral | Vitamin E (α-tocopherol (100mg/day),  β-carotene  (6 mg/day), vitamin C  (100 mg/day), selenium  (50 μg/day) | Daily intake of one antioxidant supplement containing:  α-tocopherol (100  mg/day),  β-carotene  (6 mg/day), vitamin C  (100 mg/day), selenium  (50 μg/day) | Randomised placebo-controlled trial.  Subjects were two groups of middle-aged men: survivors of myocardial infarction and healthy controls  randomised to receive the antioxidant supplement or placebo.  Duration: 12 weeks. | 106 males: 46 survivors of myocardial infarction and 60 healthy controls  Average age: 55 years | Lymphocyte | Plasma vitamin C, vitamin E, β-carotene, and selenium increased by 40%, 53%, 76%, 46% respectively  (P<0.05 for all) | Supplementation reduced MN frequency by 54% (P < 0.015) in myocardial infarction survivor subjects with normal plasma folate levels (>3.4ng/ml).  There was no effect in healthy controls. | 7 |
| Fenech M et al 2005. | Vitamin  Mineral | Vitamin A,  Vitamin C,  Vitamin E,  Zinc | Daily dose of 18 mg β-carotene, 900 mg ascorbic acid, 250 mg  d-α tocopheryl succinate and 12 mg Zn (Beta-ACE, Vitaglow Pty Ltd,  Australia) | Randomised controlled intervention.  Duration: 26 weeks | Healthy subjects.  Average age:  47.7 years  87 males  102 females | Lymphocytes | Plasma ascorbic acid, α-tocopherol and β-carotene increased by 27%, 55%, 500% respectively in the group randomised to receive the supplement. Plasma Zn was not altered. | The net effect of the supplement treatment was a 13% reduction in MN frequency (P < 0.038) relative to controls. | 6 |
| Fenech M et al. 1997a | Vitamin | Folic acid | Daily dose of 0.7 mg folic acid (as a supplement in cereal) or 2.0 mg folic acid (in a tablet) over a period of 4 months | Placebo-controlled, double-blind intervention study.  Duration: 8 weeks of  0.7 mg folic acid supplementation or placebo, followed by 8 weeks of supplementation with 2.0mg folic acid or placebo. | Healthy subjects  Age: 50-70 years  64 males | Lymphocyte | There was a 400% increase in plasma folate, 260% increase in red blood cell folate and a 11% reduction in plasma homocysteine at the end of the intervention | MN frequency was not altered by supplementation with 0.7mg or 2.0mg folic acid. | 6 |
| Fenech M et al. 1997b | Vitamin | Vitamin E | Daily dose of 50mg vitamin E (as a supplement in cereal) or 335mg (500IU) vitamin E (in soybean oil in a capsule) over a period of 16 weeks (8 weeks for each dose). | Placebo-controlled, double-blind intervention study.  Duration: 8 weeks of 50mg vitamin E supplementation or placebo, followed by 8 weeks of supplementation with 335mg Vitamin E or placebo. | Healthy subjects  Age: 50-70 years  60 males | Lymphocyte | Plasma vitamin E increased by 89% (P<0.0001) by the end of the intervention in the treatment group relative to baseline. There was no change in plasma vitamin E in the placebo group. | MN frequency was not altered by vitamin E supplementation in this intervention. | 6 |
| Stopper H et al 2008 | Vitamin | Folic acid,  Vitamin B12 | 15mg folic acid (thrice weekly);  1000μg vitamin B12 as hydroxyl-cobalamin injection once per week. | Controlled intervention with three arms:  (i): Control group  (ii): folic acid group  (iii): folic acid + vitamin B12 group  Duration: 17 weeks | Long-term haemodialysis patients  Average age: 64 years  8 females, 19 males | Lymphocyte | Homocysteine a metabolic indicator of folate and vitamin B12 status was reduced after 12 weeks in the folic acid group and the folic acid + vitamin B12 group by 33% and 50% respectively relative to the control group (P = 0.04) | MN frequency was reduced by 41% (P = 0.004) and 25% (P = 0.008) in the folic acid+vitamin B12 group and the folic acid group respectively following 17 weeks of intervention.  MN frequency in the control group did not change. | 6 |
| Volkovova et al. 2005 | Vitamin  Mineral | Vitamin E (α-tocopherol (100mg/day),  β-carotene  (6 mg/day), vitamin C  (100 mg/day), selenium  (50 μg/day) | Daily intake of one antioxidant supplement containing:  α-tocopherol (100  mg/day),  β-carotene  (6 mg/day), vitamin C  (100 mg/day), selenium  (50 μg/day) | Randomised placebo-controlled trial.  Subjects were two groups of middle-aged men differing in smoking status. randomised to receive the antioxidant supplement or placebo.  Duration: 12 weeks. | 124 middle-aged men differing in smoking status  Average age: 48 years.  64 men (22 smokers, 42 non-smokers) were on the antioxidant supplement.  60 men (25 smokers, 35 non-smokers) received the placebo. | Lymphocyte | Plasma vitamin C, vitamin E, β-carotene, and selenium increased by 51%, 22%, 59%, 28% respectively (P<0.05 for all) in the antioxidant treatment group.  Antioxidant supplementation significantly reduced malondialdehyde, a biomarker of lipid peroxidation, by 35% in non-smokers (P < 0.01) | Antioxidant supplementation did not alter MN frequency.  However, MN frequency was significantly positively correlated (R = 0.347, P < 0.001) with plasma malondialdehyde, a biomarker of lipid peroxidation. | 6 |
| Wu J. et al 2009 | Mineral,  Amino acid | Seleno-methionine | 102μg, 204μg, 306μg seleno-methionine per day provided in puffed wheat biscuits | Randomised double-blind placebo-controlled trial with three arms:  (i): placebo wheat biscuit  (ii): wheat biscuit biofortified with seleno-  methionine  (iii): wheat biscuit process fortified with seleno-methionine.  Duration: 24 weeks  (8 weeks per dose of seleno-methionine) | Healthy men with low plasma selenium concentration.  Age: 40-70 years  81 males randomised to the 3 treatment arms (27 per group) | Lymphocyte | Plasma selenium concentration increased 57% and 13% at the end of the intervention relative to base-line in the groups consuming wheat biscuits bio-fortified or process fortified with seleno-methionine respectively. There was no change in the placebo group. | MN frequency was not significantly altered by the ingestion of biscuits process-fortified or bio-fortified with seleno-methionine. | 6 |
| Crott JW et al. 1999 | Vitamin | Vitamin C | 2g vitamin C | Double-blind placebo- controlled intervention, with a cross-over.  Duration: A single dose of 2g vitamin C on one day.  Blood samples collected before and 2 or 4 hours after ingestion of vitamin C and challenged with 512μM hydrogen peroxide. | Healthy, non-smoking male volunteers,  Age: 21- 44 years  11 males | Lymph-ocytes | Plasma vitamin C concentrations increased by 115 and 125% after 2 and 4 h, respectively  (P < 0.05). | Vitamin C ingestion did not alter micronucleus expression or apoptosis in control or hydrogen peroxide-treated lymphocytes, but it moderately increased necrosis (P < 0.08). | 5 |
| Gaziev A et al 1996 | Vitamin  Phyto-nutrient | Vitamin A,  Vitamin C,  Vitamin E,  Folate,  Rutin | β-carotene (15 mg/day),  Retinol acetate (3 mg/day),  α-tocopherol  (30 mg/day) ascorbic acid (150 mg/day), folic acid (0.2 mg/day),  Rutin (75mg/day), | Longitudinal intervention without placebo control.  Duration: Blood was  sampled every 17 weeks for one year in all participants. After the first sampling of blood, the participants in groups (i) old adults and (ii) young adults had the supplement whilst a third group (iii) of old adults did not.  After the second blood sampling, intake of supplement was terminated. The third blood sample was taken  4 months after termination of supplement intake. | Three groups of healthy donors of different age were recruited:  (i): 56-83 years old (35 subjects),  (ii): 23-30 years old (13 subjects),  (iii): 63-82 years old (12 subjects).  Only groups (i) and (ii) had the supplement. | Lymphocytes | The level of total antioxidants in whole blood was determined from their reaction with the stable free radical l,l-diphenyl-2-picrylhydrazyl (DPPH).  In participants who consumed the supplement for 4 months (groups (i) and (ii)), the antioxidant content in the blood significantly increased 33% independently of age  (P < 0.01), | The older participants in group (i) showed a statistically significant 25% decrease (P < 0.01) in spontaneous MN in  lymphocytes after a 17 week period of consumption of the supplement. There was no change in young participants in group (ii).  Intake of the supplement by both aged and young donors  promoted a 44% decrease in MN induced in lymphocytes in vitro  by γ-radiation. | 5 |
| Holland N et al 2007 | Vitamin | Folate | 1mg folic acid per day | Longitudinal prospective study without placebo group.  Duration: 4 weeks.  Blood was collected before and after the intervention. | Three groups of children received the intervention with 1mg folic acid per day:  (i): Healthy children (HC) N=28, 54% male  (ii) Children with Crohn’s disease (CD)  N=24, 65% male  (iii) Children with Ulcerative colitis. N=17, 46% male  Average age: 12.1 years | Lymphocytes | Folate increased by 26%, 15%, 11% in HC, CD, UC respectively. | There was no change in MN frequency in HC. MN frequency in CD children declined by 35% (P=0.12). In contrast MN frequency in UC cases increased by 340%. | 5 |
| Joksic G et al (2009) | Phyto-nutrient | *Echinacea purpurea* | Two 275mg *Echinacea purpurea* tablets (Echinacea, Strong Nature, Serbia) twice a day. | Longitudinal intervention without placebo control.  Duration: 2 weeks  Blood samples collected before and after | Radiology technicians identified as carrying dicentric chromosomes  Average age : 38.7 years  10 subjects | Lymphocyte | *Echinacea purpurea* polyphenols were not measured in blood. | 26% decrease in MN frequency  P = 0.046 | 5 |
| Mrdanovic J. et al 2012 | Vitamin  Mineral | Vitamin A  and  Vitamin C  and  Vitamin E  and  Selenium | A supplement capsule (Oligogal Se) consumed once each day. The capsule contained:  100μg organic selenium in yeast, 90μg vitamin C, 516μg vitamin A, 45mg vitamin E. | Longitudinal intervention without placebo control.  Duration: 26 weeks | 15 oncology nurses exposed to cytotoxic drugs.  Age: 28-56 years | Lymphocyte | Plasma vitamin A, vitamin C, vitamin E and selenium was not measured. | There was a 13% reduction in MN frequency (P < 0.05) after 26 weeks of supplementation with the vitamins A, C, E and selenium supplement. | 5 |
| Nakamura et al. 2017 | Phyto-nutrient | Tomato juice | Daily consumption of 190g of tomato juice containing 17mg lycopene and 0.25mg  β-carotene. | Longitudinal intervention without placebo group.  Duration: 3 weeks of daily intake of tomato juice followed by 3 weeks refraining from it. | Healthy subjects  Average age:  23 years  5 males, 5 females | Lymphocytes | Plasma β-carotene and lycopene increased by 26% and 50% respectively after tomato juice consumption phase. | Base-line MN frequency declined by 24% and MN induced by 0.5Gy ionising radiation (X-rays) were reduced by 16% (P < 0.05).  MN frequency values returned to what they were prior to intervention after the 3 weeks of wash-out. | 5 |
| Ni J. et al 2012. | Vitamin | Folate  Vitamin B12 | 200-400μg folic acid per day with 3-25μg vitamin B12.  Dosage depended on age and MTHFR C677T genotype.  Dose was higher if older than 40 years and/or if carrier of T allele. | Longitudinal intervention without placebo group.  Duration: 16 weeks  Blood samples were collected before and after the intervention | Healthy volunteers whose MN frequency was higher than the median for their age and gender group.  Age: 23-61 years. 6 males, 6 females | Lymphocytes | Blood levels of folate and B12 were not measured. | MN frequencies in lymphocytes were reduced by 33.5%. | 5 |
| Rompelberg CJM et al (1996) | Phyto-nutrient | Eugenol  (main component of oil of cloves) | 150mg Eugenol  Per day | Placebo-controlled intervention with cross-over design.  Duration: 1 week | Healthy non-smoking subjects  Age: 21-26 years  10 males | Lymphocyte | 24 hour urines of all participants on 150mg Eugenol per day were positive for Eugenol. Results were negative in controls. | No effect on *in vivo* spontaneous MN frequency or *in vitro* mutagen-induced MN frequency was observed. | 5 |
| Schupp N et al 2008b | Vitamin | Vitamin B1 (Thiamine) | Vitamin B1 prodrug benfotiamine (Worwag Pharma, Germany),300mg daily for the first 6 weeks followed by 450mg daily for the next 6 weeks. | Longitudinal intervention without placebo control.  Duration: 12 weeks | 15 haemodialysis patients  Average Age: 63.6 years  9 male, 6 female | Lymphocytes | Blood thiamine concentration was not measured. | MN frequency declined by 31% (P < 0.001) after the intervention relative to base-line. | 5 |
| Titenko-Holland N et al. 1998. | Vitamin | Folate | Folic acid doses as follows:  Week 1:  195μg/day,  Weeks 2-6:  56μg/day,  Weeks 7-10:  111μg/day,  Weeks 11-12:  286μg/day,  Weeks 13-14:  516μg/day | Longitudinal folate depletion followed by folate repletion protocol, without placebo control in a metabolic unit.  Duration:  5 weeks depletion,  7 weeks repletion  Blood was collected weekly. | 9 healthy post-menopausal women.  Age: 49-63 years. | Lymphocytes | Plasma folate declined by 54% during the depletion phase and increased by 68% during the repletion phase. | MN frequency increased by 35% during the depletion phase (p=0.037) and decreased by 30% during the repletion phase (p=0.028). | 5 |
| Vaglenov A. et al. 1998. | Vitamin  Mineral | Centrum supplement  Vitamins:  Vitamin A 3500 IU, Vitamin C 60 mg, Vitamin D 400 IU, Vitamin E 30 IU, Vitamin K 25 mcg, Thiamin 1.5 mg, Riboflavin 1.7 mg, Niacin 20 mg, Vitamin B6 2 mg, folic acid, 400 mcg, Vitamin B12 6 mcg, biotin 30 mcg and pantothenic acid 10 mg.  Minerals: Calcium 200 mg, iron 18 mg, phosphorus 20 mg, iodine 150 mcg, Magnesium 50 mg, zinc 11 mg, selenium 55 mcg, copper 0.5 mg, manganese 2.3 mg, chromium 35 mcg, molybdenum 45 mcg, chloride 72 mg, potassium 80 mgc, boron 75 mcg, nickel 5 mcg, silicon 2 mcg, tin 10 mcg and vanadium 10 mcg. | Two tablets per day of Centrum (Lederle, 1998) | Longitudinal intervention without placebo group.  Duration: 16 weeks  Blood samples were collected before and after the intervention.  Base-line frequency of MN was measured as well as MN frequency induced by 1Gy of γ-rays | Apparently healthy lead-exposed male workers.  Average age: 37 years  22 males | Lymphocytes | Blood concentration of vitamins and minerals was not measured | Base-line MN frequency was reduced by 69% after 4 months of Centrum daily intake.  Radiation induced MN frequency was reduced by 27% after 4 months of Centrum daily intake | 5 |
| Bianchi L et al. 1993. | Vitamin  Phyto-nutrient | β-carotene (BC) with Canthaxanthin (CX) | Weeks 0-4:  40mg BC + 40mg CX per day  Weeks 5-16:  20mg BC + 20mg CX per day  Weeks 17-32:  10mg BC + 10mg CX per day  Weeks 33-52:  No supplementation | Longitudinal intervention without placebo control.  Duration: 52 weeks  Blood samples collected before the intervention and at end of wks 4, 8, 16, 32, 40, 48, and 52.  Base-line DNA damage and sensitivity to DNA damage induced by bleomycin challenge in vitro was measured. | 9 healthy volunteers  Age: 25-35 years  4 males, 5 females | Lymphocytes | Plasma carotenoid concentration increased in a dose-dependent manner with the highest increment in the first 4 weeks achieving a 2100% increment (P < 0.0001) | Base-line frequency of MN was not altered.  However BC + CX supplementation significantly reduced MN induced by bleomycin challenge. The decrease at weeks 4 and 8 was 30% (P < 0.05) and the greatest decrease was 50% at week 16. | 4 |
| Franzke B et al. 2015 | Vitamin  Mineral  Amino acids | Fortifit Nutricia:  19.7g whey protein,  3g leucine,  10g essential amino acids,  800IU Vitamin D, 250mg Calcium, Magnesium, vitamins C, E, B6, B12. | 1 portion Fortifit per day | Longitudinal intervention without placebo control with three treatment groups:  (i): cognitive training (CT)  (ii): resistance training (RT)  (iii) RT + Fortifit  Duration: 26 weeks  Blood samples collected before, after 13 weeks, after 26 weeks. | 97 elderly women and men mentally and physically capable to participate and free from severe disease.  Age: 65-98 years  13% male, 87% female | Lymphocytes | Red cell folate increased 43% (P = 0.018) and plasma B12 increased 130% (P = 0.006) in the RT + Fortifit group only. | There was a non-significant 19% decrease in MN frequency in the RT + Foritfit group in which there was also a significant negative correlation between of the B12 plasma level and MN frequency (R = -0.584,  P = 0.009). | 4 |
| Greenrod W et al 2005 | Phyto-nutrient | Red wine  De-alcoholised red wine  13% ethanol solution | 300ml Red wine  300ml De-alcoholised red wine  300ml 13% ethanol solution | A 3-arm crossover intervention study involving consumption of (i) complete red wine, (ii) dealcoholised red wine and (iii) 13% ethanol solution on separate  occasions 1 week apart.  Duration:  A single dose on one day.  Blood samples were collected before and 0.5, 1.0 and 2.0 hours after beverage ingestion. | Healthy controls  Six young adult males aged 21–26 years. | Lymphocytes | Plasma catechin concentration  increased in the de-alcoholised red wine intervention. The area under the plasma concentration  versus time curve values were 0.38, 1.82 and 0.59 for red wine, de-alcoholised red wine and 13% alcohol, respectively | Consumption of dealcoholised red wine significantly decreased gamma radiation-induced DNA damage at 1 h and 2 h post-consumption by 20% (P = 0.002).  The observed protective effects were weakly correlated with the concentration of total plasma catechin (R = −0.23, P = 0.05) | 4 |
| Migliore et al 2004 | Phyto-nutrient | Coenzyme-Q10 | 100mg Coenzyme-Q10 per day | Longitudinal intervention without placebo control.  Duration: 2 weeks | Patients with mitochondrial diseases  Age: 29-74 years  6 males, 4 females | Lymphocyte | Coenzyme-Q10 in blood was not measured. | There was a 38% reduction in MN frequency at the end of the intervention relative to the MN frequency value before the intervention (P < 0.05). | 4 |
| Schneider M. et al 2001 | Vitamin | Vitamin C  Vitamin C and  Vitamin E | Vitamin C (1000 mg/day),  Vitamin C (1000 mg/day) and Vitamin E (335 mg/day) | Longitudinal intervention without placebo control.  1000 mg ascorbic acid daily for 7 days and then  1000 mg ascorbic acid and 335.5 mg RRR-α-tocopherol  daily for the next 7 days. | 12 healthy smoking volunteers  and 12 healthy non-smoking volunteers.  Age: 19-33 years  50% of smokers and non-smokers were male and 50% were female. | Lymphocyte | Plasma concentrations of vitamin C as well  as the ascorbate free radical were increased by 32% (p < 0.001) and 20% (p < 0.05) respectively in the smokers. The corresponding values in non-smokers, however,  did not change. | After 7 days of vitamins C and E, MN was decreased in both smokers and non-smokers, but it was more decreased in smokers by 49% (P < 0.05). In non-smokers the decrease was 33% (P<0.05). | 4 |
| Umegaki K et al 1994 | Vitamin | β-carotene  Vitamin C | 30 mg β-carotene per day  300 mg per day vitamin C | Randomised placebo-controlled trial with three treatment groups:  (i): β-carotene  (ii) Vitamin C  (iii) Placebo  Duration: 6 days | Healthy female students  Age: 20-21 years.  17 females  N= 5 or 6 participants per group | Lymphocyte | Β-carotene concentration in plasma increased by 42% in the β-carotene group.  Vitamin C increased by 29% in the Vitamin C group.  There was no change in the placebo group. | There was no effect of the treatments on spontaneous MN frequency.  However, after supplementation the lymphocytes from the β-carotene group had 15% fewer MN induced by an ionising radiation challenge (0.6Gy soft x-rays).  A significant (P < 0.01) negative correlation  between the MN frequencies of the irradiated samples  and β-carotene in plasma was found both before and after supplementation. | 4 |
| Fenech M et al 1997 | Phyto-nutrient | White wine  Red wine | 300ml dose of white wine  300ml dose of red wine | Cross-over intervention without placebo control.  Duration: A single dose on one day.  Blood samples were collected before and 1, 3, 8 and 24 hours after ingestion of wine.  Capacity of blood plasma to prevent DNA damage in lymphocytes induced by H_2_O_2_ in vitro was assessed using the CBMNcyt assay. | Four healthy male volunteers  Age: 20-45 years | Lymphocyte | The concentration of wine polyphenols was not measured in blood. | There was a strong 78% reduction of hydrogen peroxide-induced MN (P = 0.007) by the plasma samples from the blood collected 1 h after consumption of wine as compared to plasma samples from blood immediately before the consumption of wine. | 3 |
| Davari H et al 2012 | Phyto-nutrient | Green tea | 4g of green tea (Ahmad tea) infusion in 280 ml boiling water | Longitudinal intervention without placebo control.  Duration: A single dose on one day.  Blood samples were collected before and 1, 3, and 5 hours after ingestion of tea and challenged with 200cGy ionising radiation (IR) | Healthy controls  Age: 20-25 years  5 males | Lymphocyte | Concentration of tea polyphenols in blood was not measured | IR-induced MN were reduced by 49% (P < 0.05) in lymphocytes from blood samples collected 3 hours after ingestion.  There was no effect on spontaneous MN frequency and protective effect against IR-induced MN was weaker in samples at 1 hour of 5 hours after tea ingestion. | 2 |
| Hosseini-mehr SJ et al. 2008 | Phyto-nutrient | Hawthorn fruit peel methanol extract powder. | 500mg Hawthorn fruit peel methanol extract powder per day. | Longitudinal intervention without placebo control.  Duration: A single dose on one day.  Blood samples were collected before and 1, 2, and 3 hours after ingestion of hawthorn extract and challenged with 150cGy ionising radiation (IR) | Healthy non-smoking participants  Age: 25-35 years  5 males | Lymphocyte | Concentration of Hawthorn extract compounds in blood was not measured. | IR-induced MN were reduced by on average by 44% (P < 0.05) in lymphocytes from blood samples collected 1, 2 or 3 hours after ingestion of hawthorn extract.  There was no effect on spontaneous MN frequency and protective effect against IR-induced MN was strongest in samples at 1 hour after ingestion. | 2 |
| Hosseini-mehr SJ et al. 2009 | Phyto-nutrient | Hesperidin | 250mg hesperidin  Single dose | Longitudinal intervention without placebo control.  Duration: A single dose on one day.  Blood samples were collected before and 1, 2, and 3 hours after ingestion of hawthorn extract and challenged with 150cGy ionising radiation (IR) | Healthy non-smoking subjects  Age: 25-35 years  5 males | Lymphocytes | Hesperidin was not measured in the blood. | The maximum protection against IR-induced MN frequency was observed 1 hour after ingestion of hesperidin.  IR-induced MN frequency was reduced 33% but there was no effect on spontaneous MN. | 2 |
| Hosseini-mehr SJ et al. 2011 | Phyto-nutrient | Hawthorn fruit peel methanol extract powder. | 1000mg Hawthorn fruit peel methanol extract. Single dose.  HPLC analysis indicated that the extract contained chlorogenic acid and hyperoside | Longitudinal intervention without placebo control.  Duration: A single dose on one day.  Blood samples were collected before and 1, 2, and 3 hours after ingestion of hawthorn extract and challenged with 200μM methylmethane sulphonate (MMS) | Healthy non-smoking participants  Age: 25-35 years  5 males | Lymphocyte | Concentration of Hawthorn extract compounds in blood was not measured. | MMS-induced MN were reduced by 36% (P < 0.05) in lymphocytes from blood samples collected 1 hour after ingestion of hawthorn extract.  There was no effect on spontaneous MN frequency and protective effect against IR-induced MN was strongest in samples at 1 hour after ingestion. | 2 |
| Rostami A et al 2016 | Vitamin | Vitamin C  Melatonin | 300mg melatonin (Natrol)  or  300mg vitamin C (Baker, England)  or  300mg melatonin + 300mg vitamin C | Longitudinal intervention without placebo control. 3 groups:  (i): Melatonin  (ii): Vitamin C  (iii): Melatonin + Vitamin C  Duration:  A single dose on one day  Blood samples were collected before and 1,2 or 3 hours after ingestion of melatonin and/or vitamin C and lymphocytes challenged with 200cGy ionising radiation (IR) | Healthy controls  Average age:  26 years  15 males | Lymphocyte | The concentration of melatonin and vitamin C in blood was not measured. | IR-induced MN were reduced by 36%, 50% and 59% by melatonin alone, vitamin C alone and by melatonin + vitamin C respectively in lymphocytes from blood samples collected one hour after ingestion.  There was no effect on spontaneous MN frequency and protective effect against IR damage was weaker at 2h or 3h after ingestion. | 2 |
| Rostami A. et al 2016 | Vitamin,  Mineral | Vitamin E  Selenium | 800IU Vitamin E,  or  100mg Selenium,  or  400IU Vitamin E +  50mg Selenium, | Longitudinal intervention without placebo control. 3 groups:  (i): Selenium  (ii): Vitamin E  (iii): Vitamin E + Selenium  Duration:  A single dose on one day  Blood samples were collected before and 1,2 or 3 hours after ingestion of Selenium and/or vitamin E and challenged with 200cGy ionising radiation (IR) | Healthy controls  Average age:  26 years  15 males | Lymphocyte | The concentration of Selenium and vitamin E in blood was not measured. | IR-induced MN were reduced by 45%, 41% and 50% by Selenium alone, vitamin E alone and by Selenium + vitamin E respectively in lymphocytes from blood samples collected one hour after ingestion.  There was no effect on spontaneous MN frequency and protective effect against IR damage was weaker at 2h or 3h after ingestion. | 2 |

(Shading indicates study with Quality score <5)

**Supplementary Table 5B: Micronuclei (MN) in buccal cells**

| Reference | Micro-nutrient class | Nutrient/s tested | Dose/s tested | Type and duration of intervention | Health status, age, gender, and number of subjects | Cell type | Change in micronutrient concentration in blood (% change) | Effect on  Biomarker/s | TOTAL SCORE  0-7 |
| --- | --- | --- | --- | --- | --- | --- | --- | --- | --- |
| Li N et al 1999 | Phyto-nutrient | Mixed Tea | Water extract powder of green tea in capsules each containing 0.38g of green tea extract.  Daily dose: 8 capsules per day | Double-blind randomised, placebo-controlled intervention  Duration: 25 weeks | 59 Subjects with oral leukoplakia.  N = 29 treatment group  N = 30 untreated control group  62% male  Age range 23-28 years | Buccal cells | Green tea polyphenols in blood or buccal cells were not reported. | MN frequency in the leukoplakia region and in normal oral mucosa decreased by 49% and 42% respectively (P<0.01).  There was no change in the placebo group. | 7 |
| Munoz N et al 1987 | Vitamin  Mineral | Vitamin A (retinol),  Vitamin B2  (riboflavin),  Zinc | Retinol (15 mg),  riboflavin (200 mg), zinc (50 mg)/week | Randomised placebo-controlled intervention.  Subjects were randomised to either receive the placebo or the supplement containing retinol, riboflavin and zinc.  Duration: 52 weeks | Healthy subjects at risk of deficiency of retinol, and/or riboflavin and/or zinc.  N=102 (51 per group)  55% male  Age: 35-70y | Buccal cells  Oesophag-eal cells | Retinol, riboflavin or zinc level in blood or buccal cells was not reported. | No statistically significant difference in MN frequency in buccal mucosa cells.  However, a statistically  significant 39% reduction (P = .04) in MN frequency of oesophageal cells was observed in the treatment group as compared to the placebo group. | 7 |
| Stich HF et al 1984. | Vitamins | Vitamin A and other carotenoids | 150,000 international units Vitamin A per week,  or 180mg/week β-carotene  Or  180mg/week canthaxanthin. | Parallel design placebo-controlled intervention with 4 arms.  Duration: 9 weeks | 132 Betel nut/tobacco chewers participated.  Between 18-26 individuals per group completed the intervention from a start with 33 subjects in each group. | Buccal cells | Vitamin A and carotenoids concentration in the blood or buccal cells  was not reported. | Significant 58% reduction in MN frequency for Vitamin A intervention.  Significant 61% reduction in MN frequency for β-carotene intervention (P<0.001). | 7 |
| Gomez-Meda BC et al 2016 | Vitamin | Folic acid | 5mg folic acid three times daily | Longitudinal intervention  (no placebo control)  Duration: 4 weeks | Patients with diabetes.  N = 35 supplemented  with FA N = 55 control group (healthy, untreated reference group)  39% male  Average age: 37 years | Buccal cells | Folic acid concentration in blood or buccal cells was not reported. | MN frequency was reduced by 71% in the diabetes patients receiving folic acid.  Before the intervention MN frequency in the diabetes patients was 2.8-fold greater than the healthy controls | 6 |
| Holland N et al 2007 | Vitamin | Folate | 1mg folic acid per day | Longitudinal prospective intervention study without placebo group.  Duration: 4 weeks. | Three groups of children received the intervention with 1mg folic acid per day:  (i): Healthy children (HC)  N=28, 54% male  (ii) Children with Crohn’s disease (CD)  N=24, 65% male  (iii) Children with Ulcerative colitis. N=17, 46% male  Average age: 12.1 years | Buccal cells | Blood folate increased by 26%, 15%, 11% in HC, CD, UC respectively. | There was no change in MN frequency in HC. MN frequency in CD children declined by 31% (P=0.07). In contrast MN frequency in UC cases increased by 300%. | 6 |
| Stich HF et al. 1985 | Phyto-nutrients | β-carotene | β-carotene (180 mg/week, given twice weekly in 6 capsules of 30 mg each). | Placebo-controlled intervention.  Duration: 10 weeks | Healthy Inuits in Gjoa Haven, Canada.  N=23 | Buccal cells from the lower mucosa | β-carotene in blood or buccal cells was not reported. | MN frequency declined by 60% (P<0.001) in the treatment group.  There was no change in MN frequency in the placebo group. | 6 |
| Stich HF et al. 1988 | Vitamin | β-carotene,  Vitamin A | Beta-carotene (I80 mg/week) (Group I), beta-carotene (180 mg/week) plus vitamin A (100,000 IU/week) (Group Il), and placebo (Group 111) capsules were given twice weekly for 6 months under strict supervision | Randomised placebo-controlled intervention.  Duration: 25 weeks | Fishermen who chewed tobacco-containing betel quids daily and had well-developed oral leukoplakias with elevated frequencies of micronucleated cells.  N=130  Average age: 48.8 years | Buccal cells | β-carotene or retinol in blood or buccal cells was not reported. | Significant 75% reduction in MN for β-carotene group (P < 0.001), significant 71% reduction in MN for β-carotene plus vitamin A group (P < 0.001) in normal buccal mucosa cells. Almost identical MN results were obtained for buccal cells in leukoplakia. | 6 |
| Zuniga-Gonzalez GM et al.  2007. | Vitamin | Folic acid | 5mg folic acid,  3 times daily | RCT intervention – no placebo control.  Duration:  4 weeks + 2 days  (total 30 days) | N = 45 diabetic mellitus (DM) patients without folic acid intake (control group)  N=30 DM patients with folic acid intake (treatment).  Average age 35.2 years.  55% female | Buccal cells | Blood or buccal level of folate was not measured. | MN frequency in buccal cells of treatment group declined by 73% (P < 0.001). | 6 |
| Abasova OY et al 2013 | Vitamin | Vitamin A,  Vitamin C  combination | 100mg vitamin C  + 5000 IU vitamin A  Each day | Longitudinal intervention without a placebo control.  Duration:  4 weeks + 2 days  Buccal cells were collected before and after the intervention. | Healthy young adults.  N = 29  41% males.  Age: 18-21 years | Buccal cells | Vitamin C and retinol in blood or buccal cells were not reported. | MN frequency was reduced by 40%. | 5 |
| Benner SE et al 1994 | Vitamin | Vitamin E,  α-tocopherol | 400 international units of α-tocopherol twice daily | Longitudinal intervention without placebo control.  Duration: 24 weeks | 22 oral leukoplakia patients.  Average age: 58 years  56% female | Buccal cells | α-tocopherol concentration in the blood or buccal cells was not reported. | MN frequency in buccal cells declined by 80% (P<0.01) in normal mucosa and declined by 64% (P<0.01) in the leukoplakia tissue. | 5 |
| Buajeep W et al 2008. | Phyto-nutrients | β-carotene | β-carotene  (15 mg/4 times daily) | Longitudinal intervention without placebo control.  Duration: 13 weeks | Oral lichen planus patients  N = 20 (19 females,1 male), Age: 31–61 years  Average age: 48 years | Buccal cells | Retinol and β-carotene increased significantly in blood serum by 26% and 1,300% respectively. | MN frequency was reduced by 79% in cells from the oral lichen planus lesions (P<0.001) MN frequency in normal buccal mucosa was reduced by 27%. | 5 |
| Da Silva Nunes MF 2013 | Vitamin | Vitamin C | 500mg vitamin C twice daily | Longitudinal intervention (no placebo control).  Duration: 4 weeks | Females using the anti-obesity drug amfepramone.  N = 56 taking amfepramone + Vit C  N = 52 control (Vit C only)  Average age: 38.2y | Buccal cells | Vitamin C was not measured in blood or buccal cells. | MN frequency was reduced by 29%. | 5 |
| Titenko-Holland N et al. 1998. | Vitamin | Folic acid | FA doses as follows:  Week 1:  195μg/day,  Weeks 2-6:  56μg/day,  Weeks 7-10:  111μg/day,  Weeks 11-12:  286μg/day,  Weeks 13-14:  516μg/day | Longitudinal folate depletion followed by folate repletion protocol, without placebo control in a metabolic unit.  Duration:  5 weeks depletion  7 weeks repletion  Blood was collected weekly. | 9 healthy post-menopausal women.  Age: 49-63 years. | Buccal cells | Plasma folate declined by 54% during the depletion phase and increased by 68% during the repletion phase. | MN frequency was not increased during the depletion phase and decreased by 57% during the repletion phase (P = 0.02). The observed changes were also influenced by vitamin B12 status. | 5 |

**Supplementary Table 6: DNA Strand breaks, determined with the Comet assay**

| Reference | Micro-nutrient class | Nutrient/s tested | Dose/s tested | Type and duration of intervention | Health status, age, gender, and number of subjects | Cell type | Change in micronutrient concentration in blood (% change) | Effect on Biomarker/s | QUALITY SCORE  0-7 |
| --- | --- | --- | --- | --- | --- | --- | --- | --- | --- |
| Bakuradze, T, et al, 2015 | Phyto-nutrient | Coffee | dark roast coffee containing  10.18 mg/g caffeoylquinic acids (CQAs)  & 1.1 mg/g  N-methyl-pyridinium (NMA) | RCT, not blinded. N=42 per group  4 wk washout.  4 weeks of 3 x 250ml black coffee over day,  or  Placebo 750ml water. | 84 Healthy males | Leukocytes | Urinary NMP was below limit of quantification for all at baseline, and for the “water” group throughout.  For the “coffee” cohort NMP measures 2.0-28.7mg NMP/g creatinine. | 27% reduction in DNA damage in the coffee group.  DNA damage in the ‘water’ group increased over the 4 week period. | 7 |
| Choi SW, et al, 2015 | Phyto-nutrient | Green tea | 2 x 150mL of 1% w/v tea/day  (pre-rain Loong-Cheng green tea)  Daily dose estimated:  Total catechins 445;  EGCG 46;  EGC 114;  ECG 46;  Epicatechin 44;  Catechin 27. | RCT, crossover design. 2 groups.  36 weeks total  Not blinded  Tmt: Green tea  Control: hot water  12 wk 1^st^ treatment  12 wk washout  12 wk 2^nd^ treatment | 43 Type 2 diabetics | Lymphocytes | Significant increases  (p < 0.05) in catechins fasting plasma after 12 weeks tea. Median increase was 227 nmol/L. | DNA damage sig reduced (13%) by GT treatment  (p < 0.0001). | 7 |
| Deveraj S, et al, 2008 | Phyto-nutrient | Lycopene- purified capsule form | Purified lycopene  0, 6.5, 15 or 30mg/day  DSM Nutritional products, NJ  Synthetic crystalline lycopene in capsules.  1 capsule /day consumed with low fat milk. | Double blind, placebo controlled RCT.  4 groups.  Lycopene restricted diet for 2 weeks prior and during 8 weeks supplementation.  10 weeks total | 77 healthy adults | Lymphocytes | Plasma lycopene significantly increased with all doses, relative to control (p < 0.05) | 8.9% decrease in DNA damage (comet assay) in 30mg lycopene group compared to baseline (p = 0.01). | 7 |
| Lee YJ, et al, 2008 | Phyto-nutrient | *Acanthopanax senticosus* (Siberian ginseng) | *A.senticosus* leaves sourced from Yang-Gu in Kangwondo, Korea. | RCT  6 months.  2 groups.  Tmt group:  2 capsules of 500mg  *A. senticosus +*  Calcium 500mg  *3 x per day*  Control: Calcium supplement only (500mg) | 40 Healthy postmenopausal women | Lymphocytes | Not stated | Tmt group had sig (12%) reduction in DNA damage (‘tail DNA’), 14% reduction in ‘tail moment’ and 7% in ‘tail length’. | 7 |
| Alleva R, et al, 2012 | Phyto-nutrient | Ascorbic acid rich diet (whole foods) | AA-rich foods added on top of a veg rich diet.  ~600mg/day (orange juice, red fruits, kiwi, green veg).  Subjects with poor veg intake were the control group. | Controlled intervention. 2 groups, 1 treated, 1 control.  Not blinded.  1 week on AA-enriched diet (or control), followed by 3 consecutive 25 min sessions in a chamber w 100% O2, with 3 min break breathing (normal) air between each. | 46 healthy males and females | Lymphocytes | 35% increase in plasma AA. | Lymphocytes of both diet-supplemented and non-supplemented subjects showed similar basal DNA damage. AA-rich diet reduced H202-induced damage by 55% at T3  (ie. after 1 wk intervention & HBO tmt).  H202-induced damage remained 42% lower 7 days after HBO tmt. | 6 |
| Astley S, et al, 2004a | Phyto-nutrient | Carotenoid – whole foods and capsule form | 5 treatments:   1. Mixed carotene capsule (mimic 1 carrot/day, 3.7mg alpha-carotene, 8.2mg Beta-carotene, 1.75mg alpha-tocopherol in carrier oil) 2. Cooked minced carrot (200g) 3. Placebo capsule 4. Tinned mandarins (1 tin / day) 5. 60mg vit C (taken every other day). | RCT, parallel design.  3 week intervention.  Capsule-consuming groups single blinded.  Not possible for groups consuming whole foods. | 64 healthy males | Lymphocytes | Plasma lycopene increased in  Tmt 1 (sig) (numbers not provided)  No sig differences in  B-carotene.  alpha-carotene was sig elevated in Tmt 1 (approx. doubled compared w day 0) and  Tmt 2 (approx. 30% increase). | No differences between treatments (Comet assay). | 6 |
| Astley et al, 2004b | Phyto-nutrient | Lycopene  Lutein  B-carotene | 15ug/day  (3 separate RCTs) | 3 consecutive placebo-controlled crossover studies, each phase 27 days | N=28 Healthy males | PBL | Not stated | No effect | 6 |
| Bakuradze T, et al, 2011 | Phyto-nutrient | Coffee | 750ml fresh Arabica coffee per day.  580mg/L of thermally labile green coffee bean constituents (CQAs);  72mg/L NMP;  720mg/L caffeine. | RCT. Not blinded.  4 weeks washout,  4 weeks coffee,  4 weeks washout | 33 healthy males | Lymphocytes | NMP was not detected in plasma or urine after washout phases, but was quantifiable following coffee treatment, indicating bioavailability. | Coffee intake resulted in 39% reduction in DNA strand breaks (comet assay) and 44% reduction in oxidised DNA (comet assay with FPG treatment). | 6 |
| Brevik A, et al, 2011a | Phyto-nutrient (whole food) | Golden Kiwifruit | 1 or 2 kiwis/day | Randomised Crossover design.  2 x 4 weeks per treatment of 1 or 2 kiwis per day.  4 week washout in between. | 24 healthy males and females (regularly consuming ‘modest’ amount of fruit/veg) | Lymphocytes | 2 kiwi treatment resulted in plasma ascorbic acid increase of 14%, lutein 5%, B-carotene 2%.  Curiously zeaxanthin reduced 20% in the 1 kiwi tmt, and had zero effect in the 2 kiwi tmt. | Significant reduction in FPG-sensitive sites (34% after 1 kiwi, 21% after 2).  Sig reduction in endonuclease III-sensitive sites (25% after 1 kiwi, 33% after 2).  Resistance to H202 oxidation (reduced 33% 1 kiwi, 31% 2 kiwi). | 6 |
| Collins AR, et al, 2003 | Phyto-nutrient | Kiwifruit  (Whole food) | 1, 2 or 3 kiwifruit consumed per day | Crossover RCT design. No placebo.  3 x 3 weeks for each phase (1, 2 or 3 fruit per day)  1 week washout between each phase. | 14 healthy males & females | Lymphocytes | Values not provided. | Combined results showed significant reduction in DNA damage (comet assay) (p<0.001). All treatments reduced DNA damage, with the largest effect being ~25% in the 1 kiwi/day tmt.  Levels of endogenous oxidation of pyrimidines and purines in DNA were markedly decreased by 27%. | 6 |
| Gill CIR, et al, 2007 | Phyto-nutrient (whole food) | Water-cress | 85g raw w/cress consumed daily for 8 weeks.  Commercially available product: Vitacress Ltd, Southampton, UK. | RCT, crossover 8 weeks each for treatment and control phases. 7 week washout in between.  Researchers were blinded to treatment during lab analyses. | 60 adults.  (30 males,  30 females),  50/50 smokers | Lymphocytes | Plasma lutein increased by 100% and  B-carotene by 33%. | Intervention resulted in sig reduction in DNA damage (17%), oxidative damage by 23.9% and in H202 challenge by 9.4%.  Beneficial effects were stronger in smokers. | 6 |
| Giovanelli L, et al, 2011 | Phyto-nutrient | Wine poly-phenols | Dealcoholized wine.   1. High proantho-cyanidin (PA) red wine (500 mL/d, 7mg/kg b.w. PA) 2. Low-PA rose wine (500mL/d, 0.45 mg/kg PA) | RCT, crossover design.  1 month per treatment.  3 week washout in between prior to crossover. | 21 healthy postmenopausal women | Lymphocytes | No difference observed with treatment. | No significant effects on DNA strand breaks or DNA oxidation observed between treatments. | 6 |
| Ibero-Baraibar, I, et al, 2015 | Phyto-nutrient | Cocoa extract | Ready to eat diet meals supplemented with 1.4g/day cocoa extract.  645.3 mg of total polyphenol, comprising theobromine, catechins, procyanidins | Double blind RCT.  4 weeks.  2 groups, placebo & supplemented. | 50 Overweight & obese | Lymphocytes | Not stated - a correlation is reported between cocoa-derived metabolites in plasma with oxidised DNA bases (FPG-sensitive sites): methyl epicatechin-O-sulphate  (r = -0.76, p = 0.007); epicatechin sulphate  (r = -0.61, p = 0.05) | No difference in DNA damage between Tmt groups.  A marginal decrease in oxidised bases in the total group was attributed to weight loss.  Subjects with higher baseline oxidative DNA damage showed greater reduction. | 6 |
| Moller P, et al, 2004 | Phyto-nutrient (whole juice product) | Black-currant antho-cyanins | Black-currant juice (397 g/day anthocyanin, based on body weight) (produced with novel processing in the BioCentrum of the Danish Technical Uni), diluted w H20 and sweetened w aspartame.  OR anthocyanin drink (365 g/day) (prepared from blackcurrant anthocyanin concentrate (Polyphenols, Norway) + aspartame and citric acid.  OR control drink contained aspartame, citric acid, water. | RCT, placebo controlled,  parallel design,  not blinded.  All consumed a low-flavonoid diet.  Food for all 3 meals/day was provided.  Drinks consumed 3x/day with meals.  3 weeks of supplementation. | 57 healthy adults (20 males, 37 females) | Leukocytes | Anthocyanin in plasma not measured due to low bioavailability and fast excretion. | Baseline levels of DNA damage were very low.  There we no significant differences between groups following the intervention.  The blackcurrant juice group showed significant increase in FPG-sensitive sites (40%), possibly suggesting an adverse effect. | 6 |
| Mullner E, et al, 2013 | Phyto-nutrient | Vegetable & PUFA | 300g Veg + 25mL of plant oil rich PUFA/day  Oil composition:  C16:0 7%  C18:0 2%  C18:1n9c 16%  C18:2n6c 62%  C18:3n3 12% | Parallel, RCT,  8 weeks  2 groups  Control group received dietary advice only. | 76 type  2 diabetics &  21 controls | PBMCs | Sig increases in treatment group at  8 weeks:  Lutein,  a-carotene,  B-carotene,  y-tocopherol, Vit K,  linoleic acid and a-linoleic acid | Tmt reduced DNA strand breaks by 17%;  Reduction in FPG-sensitive sites by 13% at 4wks but no change at 8 wks.  Only significant in the NIDDM group. | 6 |
| Riso P, et al, 2006 | Phyto-nutrient | Lycopene  (carot-enoid) | Tomato-based drink  "Lyc-o-Mato" (250mL containing 5.7mg lycopene, 3.7mg phytoene, 2.7mg phytofluene,  1mg B-carotene). Placebo drink contained fruit syrup, colour and flavour base only. | Double blind Crossover  1 drink per day for 26 days.  Tmt, placebo and washout all 26 days per phase. Plasma carotenoids analysed throughout study to verify consistent fruit/veg intake. | 26 healthy young males and females | Lymphocytes | No change | Endogenous DNA damage was low in this cohort. No significant change following placebo or Lyc-o-Mato drinks. | 6 |
| Sampson M, et al, 2001 | Phyto-nutrient | alpha-tocopherol | 400 iu oral a-tocopherol daily for 8 weeks | Double blind, placebo-controlled RCT.  Tmt 1: a-tocopherol capsule  Tmt 2: placebo capsule  8 week intervention + 4 weeks washout | 40 type 2 diabetics +  30 controls | Lymphocytes | Plasma a-tocopherol increased 1.48-fold in T2D group and 1.54-fold in the non-T2D group. In both cases sig diff to matched controls. | Treatment had no effect on single strand breaks (SSBs) or LDL oxidative susceptibility in Type II diabetics or controls. | 6 |
| Sharif, et al, 2015 | Mineral | Zinc | Zn carnosine (chelate) 86.9mg + maltodextrin, equivalent to 20mg/d Zn.  Placebo 100mg maltodextrin  (source *Metagenics, Australia)* | Double blind RCT.  12 weeks.  2 groups, placebo & Zn supplemented. | 84 Healthy older adults.  Volunteers with low plasma Zn were randomised. | PBMC | Plasma Zn sig increased by 5.7% in Tmt group. | % tail moment and  % tail intensity both reduced significantly following Zn treatment; 7.53%, and 8.76%, respectively. | 6 |
| Weisel T, et al, 2006 | Phyto-nutrient (whole juice product) | Red mixed berry juice  (Antho-cyanins) | Red grape juice (57%)  Blackberry (18%)  Sour cherry (9%)  Black currant (9%)  Chokeberry (7%)  (total phenols 1753 mg/L catechin equiv;  Total anthocyanins 197.9 mg/L; ascorbic acid  111 mg/L)  Control juice had phenolics removed.  (total phenols 297 mg/L catechin equiv; Total anthocyanins 9.8 mg/L; ascorbic acid 23 mg/L)  (Research Institute Geisenheim) | RCT. Parallel design. 2 separate pilot studies (1 tmt and 1 control). Both followed the same 9 week format.  2 week run-in  4 weeks consuming 700ml/day of either: (Study 1, n=21)  red mixed berry juice with TEAC of 19.1mmol/L,  OR  (Study 2, n=9) control juice depleted of polyphenols (TEAC 2.4mmol/L)  3 week washout | 18 males consumed the test juice.  9 males (a subset of the original 18) then repeated the same protocol but consuming the control juice. | Leukocytes | No direct plasma measure, but oxidative markers were significantly reduced. | Significant 60% reduction in total DNA damage (basic plus oxidised DNA damage) with juice consumption. (Estimate only - based on graph). | 6 |
| Welch RW, et al, 1999 | Vitamin  Mineral  Caroten-oids | Vit C a-tocopherol  B-carotene  Selenium | 1. Ascorbic acid (AA) 350mg 2. RRR-a-tocopherol 250mg 3. B-carotene 60mg 4. Selenium 80ug as Na selenite, and 5. Ascorbic acid + RRR-a-tocopherol | RCT, double blind, placebo controlled, multiple crossover.  40 weeks total.  4 wks placebo washout between each tmt  5 treatments  x 4 weeks each | 21 males;  12 smokers  9 non smokers | Leukocytes | Plasma AA sig increase (approx. 100%).  a-tocopherol sig increase (approx. 35%)  B-carotene and Se marginal, non-sig increases in plasma. | Treatments had no effect on DNA damage.  B-carotene supplementation resulted in sig diffs between groups;  8-OHdG decreased in non smokers, but increased in smokers. | 6 |
| Wu J, et al, 2009 | Mineral | Selenium | Selenium enriched biscuits.  Each biscuit to deliver 75ug/day Se.  Both Tmt groups to receive 75ug/d, then 150ug/d, then 300ug/day | Double blind RCT.  24 weeks total.  3 groups.  All consumed  1 bisc/day for 8 wk, then 2/d for 8 wk, then 3/d for 8 wk.  Tmt 1: Biofortified biscuits  Tmt 2: Process fortified biscuits (Positive control w Se added to mix)  Tmt 3: Placebo control biscuits | 62 Healthy older men (selected for lower plasma selenium concentration) | Lymphocytes | Plasma Se increased in a time and dose-significant manner in the 2 tmt groups (but not control).  Increase was greater in the Biofort group, a 57% increase from baseline at Wk 24. | No effect of tmt on DNA damage. | 6 |
| Zhao X, et al, 2006 | Phyto-nutrients | 3 caroten-oids single or as mixture:  Lutein (natural origin)  B-carotene (synthetic)  Lycopene (synthetic)  Supplier: BASF AG (Ludwigs-hafen, Germany)  Hard gelatin capsules containing carotenoids as dry powders embedded in a matrix of hydro-colloid & sucrose. | 5 treatment groups:   1. Placebo 2. 4mg/day each of lutein,  β-carotene & lycopene combined 3. 12mg lutein/d 4. 12mg  β-carotene/d 5. 12mg lycopene/d   Supplement consumed with a breakfast containing >10g fat to maximise carotenoid absorption. | Double blind, placebo controlled RCT.  Daily dose for 56 days.  Low fruit/veg diet followed, excluding carotenoid-rich foods, for 2 weeks prior to study and for duration. | 37 post-menopausal women | Lymphocytes | Effect observed in Tmt groups from day 15, maintained through to day 56.  No effect in placebo group.  Plasma lutein increased 228% (mixture group) and 514% (12mg group).  B-carotene increased 146% (mix) and 387% (12mg).  Lycopene increased significantly (detail not provided). | Significant reduction in DNA damage in treated groups compared w baseline and placebo.  Reduction in damage was 36%, 33%, 35% and 43% for the mixed carotenoids, lutein, β-carotene and lycopene groups. | 6 |
| Biswas J, et al, 2010 | Phyto-nutrient | Curcumin with piperine | 500mg, twice per day | Placebo controlled RCT.  6 months of testing, with tmt only provided for final 3 months.  2 capsules taken twice daily for 3 months.  2 groups.   1. Tmt: curcumin + piperine (500mg) 2. Placebo capsules | 286 male and female west Bengalis recruited from 5 villages with high arsenic in aquifers.  100 vols from regions without arsenic were used for baseline DNA damage. | Lymphocytes | >500% increase in curcumin measured in plasma after 1 month of tmt, maintained for 3 months of tmt. | Curcumin with piperinetreatment had a significant protective effect on DNA damage  (p < 0.001).  Data not specified, but from the graph the reduction in DNA damage is 30%-50%/month, with a total reduction of approximately 80% over 3 months. | 5 |
| Brevik A, et al, 2011b | Phytonutrient (whole food) | Kiwifruit (assume green as not otherwise stated) | Whole kiwifruit (3/day)  OR variety of 27 antiox rich fruit & plant products (eg green tea, various juices, berries, grapes, brassica veg, dark chocolate, nuts, seeds, olive oil, herbs). | RCT – parallel design.  8 weeks  Not blinded.  A moderately restricted diet followed throughout by all 3 groups.   1. 3 kiwi per day 2. Antioxidant rich plant products 3. Control group | 102 ‘healthy’ males smoking >5 cigarettes/day and taking part in CVD screening program. | Lymphocytes | Plasma measures not conducted. | DNA strand breaks reduced by 13% (not sig) in kiwi group, and 20% (sig) in Phyto group.  No effect of FPG or Endo III sensitive sites.  Significant results recorded for repair mechanisms in both kiwi and phyto groups. | 5 |
| Erba D et al., 2005 | Phyto-nutrient | Green tea | 2 cups/day equiv to 250mg of total catechins | RCT. 2 groups of 12 (tmt & control).  42 days.  No placebo.  All consumed low antioxidant diet. | 24 healthy females | Lymphocytes | Plasma polyphenols measured as units of gallic acid (ug). GT tmt resulted in a non-sig increase.  Sig increase in plasma total antioxidant activity  (p < 0.001). | Approx 30% reduction in DNA damage (tail moment) (p < 0.05) | 5 |
| Erba D, et al, 2012 | Phyto-nutrient | Soy isoflavone (capsules) | 80mg/day  2 capsules, one morning, one evening.  Each contained 40mg aglycone IF equivalents;  21mg total genistein;  15mg daidzein;  4mg total glyceitein.  Commercial product “Glycinemax” (Longlife, Milan, Italy) | Prospective, 6 months, No control group. | N=22 women  (9 postmeno-pausal,  13 premenopausal) | PBMCs | Plasma concn’n of genistein and daidzein increased significantly for duration of intervention (~15-fold increase) | Sig reduction in damage (11-15%). Sig inverse correlations between damage and plasma genistein & daidzein. | 5 |
| Hoelzl C, et al, 2010 | Phyto-nutrient | Coffee | 4 x 200mL coffees/day  (Nestle packaged coffee containing 3.4g, 35% green and 65% roasted coffee extracts) | RCT – crossover design. Not blinded.  Low polyphenol diet followed prior and during study.  1 wk baseline diet  5 d intervention (1)  6 wk washout  5 d intervention (2) | 36 adults (males and females) | Lymphocytes | Not directly measured. | FPG and Endo III sensitive sites reduced by 14.1 and 12.5 % respectively following coffee consumption (not significant). | 5 |
| Park YK, et al, 2003 | Phyto-nutrient (Whole food) | Anthocyanins | Purple grape juice | Prospective, No placebo, control, not blinded.  480mL juice consumed twice daily for 8 weeks | 67 healthy adults (51M, 16F)  29M were smokers | Lymphocytes | 19% reduction in total free radical counts (plasma) | Grape juice significantly reduced DNA damage by 25% in smokers and 18% in non-smokers. | 5 |
| Riso P, et al, 2004 | Phyto-nutrient & vitamin (Whole foods) | Lycopene Vitamin C | Tomato products providing a mean of 8mg lycopene,  0.5mg B-carotene and 11mg vit C/day. | Intervention (not placebo controlled or blinded).  1 week basal diet (low in carotenoids), then 3 weeks basal diet + one of a range of tomato products daily (raw tomato, sauce, or paste). Each consumed with 10g olive oil. | 25 healthy women | Lymphocytes | After 3 weeks of treatment, lycopene and vit C increased significantly in plasma (53%, 73%) and in lymphocytes (35%, 230%). | Tomato diet significantly reduced DNA damage by 24%. | 5 |
| Shaposhnikov S, et al, 2018 | Phyto-nutrient | Coffee | Tmt 1: 5 cups (0.15L) coffee/day  Tmt 2: 3 cups/d + 2 cups water  Tmt 3: 5 cups water  (Full breakdown of each sachet is provided) | Placebo controlled RCT.  8 weeks, 3 groups.  5 week washout for coffee and antioxidant-rich foods prior.  Roasted, ground 100% Arabica. Source, *Kraft Foods* | 160 Healthy adults | Leukocytes | No data provided. | No significant differences between treatment groups with regards to DNA strand breaks or oxidised DNA. | 5 |
| Spormann TM, 2008 | Phyto-nutrient (whole juice product) | Red fruit juice rich in antho-cyanin | Mixed juice: red grape (40%), blackberry (20%), sour cherry (15%), blackcurrant (15%), elderberry (10%)  Antiox capacity (TEAC value) was very high (31.3 mmol/L);  High total phenols (3,478 mg/L catechin equivalents);  Anthocyanins  (301 mg/L cyanidin-3-glucoside equivalents) | RCT, prospective (no control or placebo), not blinded.  Low ‘red’ food diet followed throughout.  3 week run-in  4 week juice consumption  3 week washout | 21 clinically stable haemodialysis patients | Leukocytes | Antioxidant capacity of plasma (TEAC) was not sig modulated by the juice treatment. | The 4-week juice treatment resulted in highly sig decrease of total DNA damage (DNA strand breaks plus oxidised DNA damage TI%); reduced from 5.22 to 2.85 (45% reduction), maximally expressed in the 2^nd^ week of consumption. These effects were mainly due to reduced oxidised DNA damage. | 5 |
| Sporrmann et al, 2008 | Phytonutrient | Red berry juice (combination of 5 berries) | 2 x 100mL juice serves per day (morning and evening) | 4 week intervention (3 week wash out before and after) | Haemodialysis patients  N=21  14 males | Lymphocytes, Comet assay | Not measured | 45% reduction in DNA damage (tail intensity %) after 2 weeks (p < 0.0001) | 5 |
| Wilms LC, et al, 2007 | Phyto-nutrient (whole juice product) | Blueberry & apple juice (quercetin) | Juice containing 97mg quercetin & 16mg ascorbic acid (AA).  Manufactured specifically by Riedel Drinks, Ede, The Netherlands. | Intervention, prospective, not blinded, no placebo control.  All followed a low-flavonoid/quercetin diet throughout, & minimise use of herbs/spices.  5 days washout. + 4 week intervention. | 168 healthy adult non-smokers (54 males, 114 females) | Lymphocytes | Plasma concentrations sig increased from baseline after 4 wks; quercetin from 28.8 nM to 79.2nM;  AA from 58uM to 61uM; and antioxidant capacity (TEAC) from 781 uM to 800uM. | A significant (20%) reduction in H202-induced DNA damage was observed following intervention.  A larger protective effect was observed in men, than women.  Analyses were also conducted on SNPs relevant to these metabolic pathways. | 5 |
| Bakuradze, T, et al, 2016 | Phyto-nutrient | Coffee | Arabica coffee | 8 hour acute study  200mL coffee consumed every 2 hours up to 800mL  Blood sampled at baseline and 2 hourly. | N=13  Males aged 20-50 | Leukocytes, Comet Assay | Not measured | Tail intensity decreased from 0.33% to 0.22% after coffee intake (p < 0.001) | 4 |
| Ferk F, et al, 2011 | Phyto-nutrient | Phenolic: Gallic acid | 12.8 mg/person / day added to water, consumed at 9am over 2 hours. | RCT, placebo controlled.  Drinking water supplemented (or control) consumed for 3 days.  A restricted diet was consumed for 7 days prior, and 7 days after. | 16 healthy adults (8 males,  8 females)  N=8 per group (tmt and control) | Lymphocytes | GA was detectable in plasma for up to 2 hours post ingestion (average of 0.12 ± 2ug/mL). Levels then declined below detection limit. | GA treatment had no effect in standard comet assay.  GA was protective against H202 and FPG-induced damage, with a sig reduction  (both p = 0.001). | 4 |
| Lim et al 2013 | Phyto-nutrient | Asiatic plantain beverage (herbs purchased at Korean market) | 80 mL drink containing 5, 10 or 15mg of processed extract | 6 hour acute  RCT, double blind. | 40 overweight, hyperlipidemic subjects  n= 10 per group  1 control group &  3 increasing doses treatment groups | Lymphocytes, Comet assay | Not measured | At 360 mins post consumption DNA damage was reduced compared with placebo (p < 0.0225) | 4 |
| Moser B, et al, 2011 | Phyto-nutrient (whole food) | Spinach | 225g/day | RCT, not blinded.  5 day run-in  16 days of homogenised spinach + 8g corn oil to aid carotenoid absorption.  Control group consumed corn oil only. (Not clear how many in the control group – assume separate to 8 in treatment group?)  40 days washout. | 8 healthy adults  (4 male, 4 female) | Lymphocytes | Spinach consumption resulted in 27.2% increase Folic acid concentration (sig).  Homocysteine reduced (sig) by 16.6%.  Vitamins C and B12 increased but not sig.  CRP reduced 5.4%, not sig. | DNA damage no change.  H2O2-induced damage reduced by 24.2% in spinach group.  Effects seen immediately upon spinach consumption and maintained throughout. | 4 |
| Pool-Zobel et al, 1997 | Phyto-nutrient (whole foods) | Caroten-oids  (a- and B-carotene) Lycopene Lutein | Tomato juice, carrot juice, spinach powder dissolved in water or milk. 1 drink/day | Crossover intervention.  No apparent washout between treatments.  Not blinded  8 weeks total:  2 wks low-carotenoid diet  2 wks tomato juice  2 wks carrot juice  2 wks spinach powder dissolved in water | 23 healthy males | Lymphocytes | 2-300% increase in plasma lycopene after tomato juice;  Approx. 400% increase in carotenoids after carrot juice;  Approx. 200% increase in lutein after spinach juice. | DNA damage is reduced from baseline with the tomato treatment by approximately 37%. Replacing tomato with carrot or spinach did not alter the initial 37% reduction substantially. The study lacked a concurrent control or a depletion phase to verify the observed effect properly. | 4 |
| Porrini M, et al, 2002 | Phyto-nutrient (whole foods) | Carotenoids; Lycopene, lutein, B-carotene,  zea-xanthin | 150g Spinach (providing 9mg lutein, 0.6mg zeaxanthin, 4mg  B-carotene) (eaten cooked w 10g olive oil)  Tomato puree (consumed cold) (25g, providing 7mg lycopene, 0.3mg B-carotene) | Crossover design:  3 weeks spinach diet,  2 week washout,  3 weeks spinach + tomato diet.  Total of 9 weeks on low carotenoid basal diet (incl 1 wk prior). | 9 healthy women | Lymphocytes | Carotenoids in lympho-cytes after the combined (spinach + tomato) diet: lycopene increased 7-fold, lutein increased 3-fold, B-carotene increased 2-fold. | Both diets resulted in significant reductions in DNA damage following oxidative insult (H202, comet assay).  Specific values not provided, but graphs suggest approx. 50% reduction w Spinach, and 25% reduction with the combined diet. | 4 |
| Heger A, et al, 2012 | Phytonutrient | Resveratrol containing dietary supplement | A commercially available tablet (not stated) containing:  2mg resveratrol (RES) from grapes,  100mg dried grape extract,  50mg dried extract from olive oil,  3mg lycopene,  100mg Vit C and  30mg bioflavonoids from citrus fruits. | Prospective, no placebo, not blinded.  5 days washout  5 days of 3 tablets/day (morning, noon, evening)  10 days washout | 12 healthy  (5 males, 7 females) | Lymphocytes | No impact on biomarkers of redox status. | No effect on DNA damage measured under standard conditions, H202 challenge or FPG. | 3 |
| Herro-Barbudo C, et al, 2013 | Phyto-nutrient | Lutein  (carot-enoid) | Lutein enriched fermented milk product (Lutein esters mix, Cognis GmbH)  4mg free lutein/100mL. Daily dosage of 4-8mg/day equiv to 150-200g cooked spinach and below suggested safety intake of 20mg/d. | Prospective study, no placebo control.  14 days  2 groups.  Tmt 1. 4mg/100mL /day  Tmt 2.  8mg/100mL /day | 24 Healthy men and women  Note comet analysis only done on the 10 subjects whose serum lutein concentration reached epidemiological and biological significance (0.4-0.45 umol/L and 0.6-1.05 umol/L) | Lymphocytes | 212% increase in serum lutein after 14 days. | No significant effect on DNA damage between treatments.  Endogenous DNA damage reduced by 12%, and H202-induced damage reduced by 13%, but these were not significant. | 3 |
| Silvestri, S, et al, 2015 | Phyto-nutrient | Coenzyme Q10 | 200 mg/day alone  or with 200 mg/d  a-lipoic acid (LA) | Prospective RCT.  No placebo.  15 days. 2 doses, lunch and dinner.   1. 200 mg/day CoQ_10_ alone 2. 200mg/day CoQ_10_ + LA | 16 healthy adults  (8 male, 8 female) | Lymphocytes | Across both groups a 241% increase in CoQ10 in plasma (sig). | Treatment had no effect on DNA damage induced by H202 incubation. | 3 |
| Torbergsen AC, et al, 2000 | Phyto-nutrient | Lycopene and Lutein | **Lycopene 15mg/day:**  Capsules: Lycopene w 10% B-carotene. (Makhtesheim Chemical works Ltd, Beer-Sheva, Israel. Capsules prepared by R. P. Scherer International Corp, Israel)  **Lutein 15mg/day:**  Capsules: 80% trans-lutein, 20% 13-/15-cis-lutein (Quest International, Cork, Ireland. Capsules prepared by R. P. Scherer Intn’l Corp.) | Crossover with 3-week washout between each treatment | 8 Healthy adults,  5 Male 3 Female | Lymphocytes | 17% increase in plasma lycopene. Not sig.  Wide inter-subject variability in plasma lutein post-tmt (10%-700% increase) | Effect of supplement varied considerably between subjects. Data not provided. | 3 |
| Tulipani S, et al, 2014 | Phyto-nutrient | Strawberries, whole, Sveva variety | 500g/day strawberries picked within 3 days. | Prospective, 14 days, no control group. | 18 healthy males and females | PBMCs | Non-significant increases in plasma antioxidant status. Measurement of polyphenols (anthyocyanins) in blood or urine was not conducted. | Untreated cells - no change in DNA damage.  Cell death in H202-treated cells significantly reduced after 14d strawberries. | 3 |
| Wilms LC, et al, 2005 | Phyto-nutrient | Quercetin | Combination of commercially available quercetin-rich juices:  Equal volumes of  Blueberry juice (34mg quercetin/L) (Natufood, Harderwijk, The Netherlands)  &  Apple juice (1.5mg quercetin /L)  (Riedel, Ede, The Netherlands) | Prospective pilot study  5 day washout (low quercetin-rich foods, herbs and spices)  4 weeks consuming 1L juice mixture/day  1Litre consumed throughout day = 18mg quercetin | 8 healthy females | Lymphocytes | 4 week treatment resulted in plasma TEAC increase from 733 to 855uM (sig) and Plasma quercetin content from 5.0 to 10.6nM (sig). | DNA damage following H202 challenge decreased by 41% from baseline (not significant). | 3 |

(Shading indicates study with Quality score <5)

**Supplementary Table 7: DNA Oxidation
*(excluding studies measuring DNA oxidation by Comet assay, which are shown in Table 6)***

| Reference | Micro-nutrient class | Nutrient/s tested | Dose/s tested | Type and duration of intervention | Health status, age, gender, and number of subjects | Cell or body fluid type | Change in micronutrient concentration in blood (% change) | Effect on Biomarker/s | QUALITY SCORE  0-7 |
| --- | --- | --- | --- | --- | --- | --- | --- | --- | --- |
| Devaraj S, et al, 2008 | Phyto-nutrient | Lycopene- purified capsule form | Purified lycopene  0, 6.5, 15 or 30mg/day  DSM Nutritional products, NJ  Synthetic crystalline lycopene in capsules.  1 capsule /day consumed with low fat milk. | Double blind, placebo controlled RCT.  4 groups.  Lycopene restricted diet for 2 weeks prior and during  8 weeks supplementation.  10 weeks total | 77 healthy adults | Lymphocytes  Urine | Plasma lycopene significantly increased with all doses, relative to control (p < 0.05) | Sig 23% reduction in urinary 8-OHdG in 30mg group. | 7 |
| Homayouni et al, 2017 | Phyto | Hesperidin | 500mg per day | RCT.  Placebo controlled  6 week intervention | N = 64 adults with type 2 diabetes.  Split into tmt and placebo | Serum  8-OHdG | Herperidin was not measured in blood | In tmt group, serum 8-OHdG decreased from baseline to 6wks (14.32 ± 6.4 vs. 11.00 ± 7.0; p = 0.000). No change in placebo group. | 7 |
| Chen L, et al, 2001 | Phyto-nutrient | Lycopene from tomato foods | 30mg lycopene/day for 3 weeks via tomato-based foods, prior to prostatectomy surgery | Prospective. Tomato-sauce based pasta dishes for 3 weeks.  No placebo. | 32 prostate cancer patients | Leukocytes | Serum lycopene sig increased 97% from  638 nM to 1258 nM. | Sig (22%) reduction in DNA oxidative damage in leukocytes (from 0.61 to 0.48 8-OHdG/10^5^ dG).  Also sig lower (29%) DNA oxidation of prostate tissue in intervention group compared with randomly selected group (0.76 to 1.06 8-OHdG/10^5^ dG). | 6 |
| Kucuk, O, et al, 2002 | Phyto-nutrient | Lycopene from tomato extract | Tomato oleoresin extract 30mg lycopene/day for 3 weeks prior to surgery. | RCT 3 weeks.  N = 15 treatment  N = 11 control group  Treatment group consumed oleoresin extract containing 15mg lycopene twice daily.  Control group given NCI advice to increase daily fruit and veg. | 26 males newly diagnosed with T1 and T2 prostate cancer | Lymphocytes | Plasma lycopene was 34% higher in treatment group (not sig). | No difference between groups for 5-hydroxy-methyl-deoxyuridine  (5-OHmdU). | 6 |
| Machowetz A, et al, 2007 | Phyto-nutrient / Fatty acids | Phenolics / Fatty acids from olive oil | Olive oil with low (2.7 mg/kg caffeic acid), medium (164 mg/kg) or high (366 mg/kg) phenolic concentrations. 25ml daily for 3 weeks. Oils were balanced for Vitamin E. Contained phenolics: Oleuropein (65%) Ligstroside aglycones (18%)  Hydroxytyrosol (11%) Tyrosol (4%) Flavonoids (1%) | Double blind RCT, crossover design.  3 weeks per treatment phase.  2 wk run-in + 2 wks washout between each of the three treatment phases. | 182 healthy males from northern (n=58), central (n=70) or southern (n=54) Europe.  Age: 20-60 yrs. | Urinary oxidised guanine | A dose-dependent increase in urinary excretion of phenolics was observed. | Urinary 8-oxo-deoxyguanosine decreased by 18% (P=0.008)) after olive oil consumption.  There was no effect of phenolic content on DNA oxidation. | 6 |
| Mullner E, et al, 2013 | Phyto-nutrient | Vegetable & PUFA rich whole diet | 300g Veg + 25mL of plant oil rich PUFA/day  Oil composition:  C16:0 7%  C18:0 2%  C18:1n9c 16%  C18:2n6c 62%  C18:3n3 12% | Parallel, RCT,  8 weeks, 2 groups  Control group received dietary advice only. | 76 type  2 diabetics &  21 controls | PBMCs &  Urinary  8-oxodG &  8-oxoGuo (dihydro-guanosine) | Sig increases in treatment group at  8 weeks:  Lutein,  α-carotene,  β-carotene,  Υ-tocopherol,  Vit K,  linoleic acid and  α-linoleic acid | Treatment had no effect on 8oxodG or 8oxoGuo. | 6 |
| Welch RW, et al, 1999 | Vitamin  Mineral  Caroten-oids | Vit C α-tocopherol  B-carotene  Selenium | 1. Ascorbic acid (AA) 350mg 2. RRR-a-tocopherol 250mg 3. B-carotene 60mg 4. Selenium 80ug as Na selenite, and 5. Ascorbic acid + RRR-a-tocopherol | RCT, double blind, placebo controlled, multiple crossover.  40 weeks total.  4 wks placebo washout between each tmt  5 treatments  x 4 weeks each | 21 males;  12 smokers  9 non smokers | Leukocytes | Plasma AA sig increase (approx. 100%).  a-tocopherol sig increase (approx. 35%)  B-carotene and Se marginal, non-sig increases in plasma. | Treatments had no effect on DNA oxidation measured as 8-OHdG.  However, β-carotene had an opposite effect in smokers and non-smokers with 8-OHdG increasing by 36% in smokers and decreasing by 57% in non-smokers during the intervention; this difference in the effect of β-carotene on DNA oxidation by smoking status was statistically significant (P<0.05). | 6 |
| Nakamura A, et al, 2017 | Phyto-nutrient | Lycopene from tomato juice | 190g commercial tomato juice /day  containing  17mg lycopene and  0.25 mg  β-carotene | Prospective RCT.  No placebo.  3 weeks treatment +  3 weeks washout  Blood samples taken at baseline, after 3 weeks treatment, and after 3 weeks washout. | 10 healthy adults  5 males,  5 females | Serum 8oxodG | Plasma lycopene increased (sig) by 33%, and β-carotene by 20%. | A 35% (non-significant) reduction in extracellular 8-oxo-dG following treatment. A non-sig negative correlation was observed between plasma lycopene and B-carotene with serum 8-oxo-dG. | 5 |
| Rao AV, et al, 1998 | Phyto-nutrient | Lycopene from whole foods and extract capsules | 5 treatments + placebo;   1. tomato juice (daily lycopene intake (DLI) 50.4 mg), 2. spaghetti sauce #1 (DLI 20.5 mg) 3. spaghetti sauce #2 (39.2 mg) 4. oleoresin capsule #1 (DLI 75 mg), 5. oleoresin capsule #2 (150 mg) 6. placebo control (DLI 0 mg) | RCT, multiple crossover design, placebo controlled.  Each treatment consumed for 1 week each. | 19 healthy adults  10 males,  9 females | Lymphocytes | Sig increase in serum lycopene with all treatments.  All were approximately double that of the placebo treatment. | DNA oxidation measured as 8-oxodG in lymphocyte DNA was reduced by 28%, 26% and 21% relative to control following consumption of spaghetti sauce #2, tomato juice and oleoresin capsule #2 respectively, however, none achieved statistical significance. 8-oxodG was not measured in the interventions with spaghetti sauce #1 and oleoresin capsule #1. | 5 |
| Simonetti, P, et al, 2002 | Phyto-nutrient | Pro-cyanidins from Vitis Vinifera (grape seed extract) | Product “Leucoselect” sourced from Indena (Milano, Italy)  2 capsules/day  (280mg) providing ~110mg of procyanidins from GSE.  GSE composition:  15% catechin, epicatechin, gallic acid  80% epicatechin gallate, dimers, trimers, tetramers, and their gallates  5% pentamers, hexamers, heptamers and their gallates  Total flavonols in each capsule ~39.6% | Prospective. 30 days intervention. | 10 healthy adults | Lymphocytes | Plasma  α-tocopherol not affected.  55% (sig) increased in  α-tocopherol in red blood cell membranes. | 67% reduction in lymphocyte oxidative DNA damage measured by ratio of 8-oxo-7,8-dihydro-2’-deoxyguanosine and 2-deoxyguanosine (P=0.049). | 5 |

**Supplementary Table 8: Telomere Length / Telomerase**

| Reference | Micro-nutrient class | Nutrient/s tested | Dose/s tested | Type and duration of intervention | Health status, age, gender, and number of subjects | Cell type | Change in micronutrient concentration in blood (% change) | Effect on  Biomarker/s | QUALITY SCORE  0-7 |
| --- | --- | --- | --- | --- | --- | --- | --- | --- | --- |
| Toupchian et al, 2018 | Fatty acid | Fatty acid | Patients took four 600 mg soft gels orally per day. Each DHA-enriched fish oil soft gel contained 600 mg omega-3 fatty acids i.e. 362.5 mg DHA plus 100 mg EPA according to the manufacturer's information (DHA Ultimate, Pure Encapsulations, Boston, USA).  Other ingredients were gelatin capsule, fish oil (tilapia), 1 IU natural mixed tocopherols, and rosemary extract (leaf). The control group received soft gels that were identical in appearance to the DHA-enriched fish oil soft gels but contained 600 mg paraffin (Zahravi, Tabriz, Iran). | Double blinded RCT,  2 groups. 8 weeks. | 72 type 2 diabetics,  30-70yrs,  positive for PPARy Pro12Ala polymorphism | PBMC | Not stated | Telomerase activity reduced in DHA group (p=0.0001).  Inversely correlated with P16 mRNA (tumour suppressor gene).  DHA possibly protective by enhancing senescence pathway.. | 7 |
| Zhu H, et al, 2012 | Vitamin | Vit D | Oral 60,000 IU/month, equiv to 2000 IU/day. | Double blind RCT.  4 months (baseline & 16wk measures)  2 groups, placebo (n=19) & Vit D supplemented (n=18). | Overweight African Americans,  n = 37 | PBMC | Serum 25-hydroxy vit D increased by 18% in controls, and by 192% in Tmt group. | Telomerase activity (TRAP assay) increased sig in Tmt group, 19.2% (p<0.0001). | 7 |
| Kiecolt-Glaser JK, et al, 2013 | Fatty acids | Omega-3 (mix of 19 F/acids, 93.3% omega 3 using a 7:1 EPA:DHA balance) | 1. 1.25g/d n-3 2. 2.5g/d n-3 3. Placebo (4.8% n-3)   All provided by *OmegaBrite (Waltham, MA, USA).* Mild fish flavour added to placebo oil. Opaque capsules. | Double blinded RCT, 3 groups.  Duration:  4 months. | Overweight sedentary mid-aged,  n = 106 | Lymphocytes | After 4m tmt, plasma total n-3 increased by 58% in 1.25g group, and 110% in 2.5g group. Placebo showed no change.  There was a significant reduction in n-6:n-3 ratio with supplementation. | Low and high dose treatment groups had a 21 base pair and 50 bp increase, respectively and placebo group decreased by 43 bp, but changes were not significant.  Lower n6:n3 ratios associated with significantly longer TL.  Tmt had no effect on telomerase activity. | 6 |
| Ornish et al 2013 | Multiple foods and micronutrient supplements | Plant-based dietary pattern plus supplements. | Doses varied depending on the nutrient content of foods consumed in dietary pattern.  The diet was supplemented with soy (one daily serving of tofu plus 58 g of a fortified soy protein powder beverage), fish oil (3 g daily), vitamin E (100 IU daily), selenium (200 μg daily), and vitamin C (2 g daily) | Longitudinal  Controlled intervention.  Duration of intervention was 5 years. | 35 men with biopsy-diagnosed low-risk prostate cancer | Peripheral blood mononuclear cells | Not reported | Telomere length in PBMCs after 5 years increased by 9.5% in the treatment group relative to baseline which was a significant improvement when compared to the 4.2% decline in the control group relative to baseline (P=0.03)  Furthermore, the degree of adherence to the diet and lifestyle factors in the intervention regime correlated positively with telomere length (P=0.005). However, telomerase activity in PBMCs decreased in both groups and although the decline was less in the intervention group it did not achieve statistical significance when compared to control. | 6 |
| Balcerczyk A, et al, 2014 | Micronutrients & w-3 fatty acids | ‘Nuclevital Q10’ Mixture supplement, | 6 capsules/day.  1 capsule contains:  w-3 (225mg being 75mg EPA & 75mg DHA)  Ubiquinone (50mg)  Astaxanthin (2.5mg)  Lycopene (7.5mg)  Lutein palmitate (5mg)  Zeaxanthin palmitate (1mg)  L-selenomethionine (55mg)  Cholecalciferol (5ug)  a-tocopherol (7.5mg) | Prospective, no control group.  4 week washout, followed by 12 weeks intervention. | Healthy women 35-55y, n = 66 | PBMCs | 25% increase in antioxidant capacity of plasma & 16% in superoxide dismutase activity.  Sig increase in BDNF (26%), Sirt 1 (26.5%), Sirt 2 (25%). | Telomerase sig increased by 34%. (Elisa kit, not Trap assay)  TL unchanged. | 5 |
| Barden A, et al, 2016 | Fatty acid & Phyto-nutrient | n-3 Fatty acids & Coenzyme Q10 | 1. n-3 FAs (4g/d, *Omacor, Solvay Pharma,* Australia) 2. CoQ (200mg/d, *Blackmores, Aust*) 3. n-3 + CoQ 4. Control (4g olive oil/d, Cardinal Health, Aust) | Double blinded RCT.  4 groups.  8 weeks. | Men and women with chronic kidney disease  n = 85 | PBMC & neutrophils | At 8 weeks platelet n-3 FAs were 6.4% higher in the n-3 group, and 5.4% higher in the n-3 +CoQ group, compared w Control. | No sig main or interactive effects of tmt on TL  Correction for neutrophil cell count showed longer neutrophil TL after n3 tmt. | 5 |
| Ornish et al 2008 | Multiple foods and micro-nutrient supplements | Plant-based dietary pattern plus supplements. | Doses varied depending on the nutrient content of foods consumed in dietary pattern.  The diet was supplemented with soy (one daily serving of tofu plus 58 g of a fortified soy protein powder beverage), fish oil (3 g daily), vitamin E (100 IU daily), selenium (200 μg daily), and vitamin C (2 g daily) | Longitudinal prospective study with participants acting as their own controls.  Duration of intervention was 30 days | 30 men with biopsy-diagnosed low-risk prostate cancer | Peripheral blood mononuclear cells | Not reported | PBMCs from blood samples collected after the intervention showed a significant 29% increase in telomerase activity relative to baseline (P = 0.031). Furthermore, telomerase activity correlated inversely with LDL cholesterol concentration in serum (r = –0·36, P = 0·041). | 5 |
| Salvador L, et al, 2016 | Phytonutrient | TA-65 (extract from plant *Astragalus)* TA-65MD is designated as GRAS (generally recognised as safe) for use in medicinal foods and is sold as a supplement by *TA Sciences*. | Tmt 1. 1 capsule (250 U) + 3 placebo caps (n = 23)  Tmt 2. 4 caps (1000 U) (n = 22)  Placebo. 4 capsules (content not stated) (n=52) | Double blind RCT, parallel groups.  1 year.  3 groups: 2 Tmt groups & placebo.  90 days of tmt, followed by 14 days of no tmt, maintained for 1 year. (Rationale not stated) | Healthy CMV-antibody positive adults, 53-87yrs  n = 97 | PBMC | Not tested/ reported. | TL decreased in placebo group by 290±100 bp/year, (p = 0.01).  Tmt 1 (low dose): TL increased by 530±180 bp/year, (p = 0.005)  Tmt 2 (high dose): TL – no change | 5 |
| Sharif, et al, 2015 | Mineral | Zinc | Zn carnosine (chelate) 86.9mg + maltodextrin, equivalent to 20mg/d Zn.  Placebo 100mg maltodextrin (source *Metagenics, Australia)* | Double blind RCT.  12 weeks.  2 groups, placebo (n = 42 )& Zn supplemented  (n = 42). | Healthy older adults. Volunteers w low plasma Zn were randomised.  n = 84 | PBMC | Plasma Zn sig increased by 5.7% in Tmt group. | Both groups showed an increase in telomere length (measured by qPCR) following 12 weeks of intervention with an increment of 7.4% (p=0.035) for the zinc supplemented group and 10.7% (p=0.054) for the placebo group relative to baseline, but between group comparisons were not significant suggesting no effect of zinc supplementation on telomere length relative to control. Telomere base damage (measured by qPCR after FPG digestion) was found to be signiﬁcantly decreased by 51% (p=0.009) in the zinc group and also decreased by 32% (p=0.096) in the placebo group but between group comparison was not statistically significant suggesting no substantial effect of zinc supplementation on telomere base damage relative to control. | 5 |
| O'Callaghan N, et al, 2014 | Fatty acids | Omega-3 PUFAs | 3 groups.  1. EPA-rich fish oil (1.67g EPA + 0.16 g DHA/d) 2. DNA-rich fish oil (1.55g DHA + 0.4 g EPA/d) Control. Safflower oil (2.2g/d Linoleic acid) | RCT. Blinded. Placebo controlled.  6 months.  Small pilot study (underpowered, but interesting) | Older adults (>65y) with mild cognitive impairment (MCI)  N = 33 | whole blood | Data not presented. States ‘compliance excellent’ and that ‘changes in erythrocyte DHA or EPA corresponded to the treatments. | TL shortened in all 3 groups, but the least amount of shortening from baseline was observed in the EPA Tmt (d = 0.06), followed by DHA Tmt (d = 0.12), and control (d = 0.21).  Change in TL was negatively associated with erythrocyte DHA (in Tmt 2). r = -0.67, p = 0.02) | 4 |

(Shading indicates study with Quality score <5)

**Supplementary Table 9:** Mitochondrial DNA mutations / copy number

| Reference | Micro-nutrient class | Nutrient/s tested | Dose/s tested | Type and duration of intervention | Health status, age, gender, and number of subjects | Cell type | Change in micronutrient concentration in blood (% change) | Effect on Biomarker/s | QUALITY SCORE  0-7 |
| --- | --- | --- | --- | --- | --- | --- | --- | --- | --- |
| Wang XM et al., 2007 | Phyto-nutrient | Chinese herbal medicine  Wuzi Yanzong Granules (WYG) | Patients in the treatment group received modified WYG (Herba Epimedii was added to Wuzi Yanzong Pill described in Pharmacopeia: Fructus Lycii 400 g, parched Semen Cuscutae 400 g, Fructus Rubi 200 g, steamed Fructus Schisandrae 50 g, parched Semen Plantaginis with salt 100 g, fried Herba Epimedii with sheep fat 400 g), one little bag at a time (4.5 g per package, containing 12 g of crude drugs) twice daily.  The placebo was completely identical to the form, package, color of active WYG. | Double-blind placebo controlled intervention  Duration:  13 weeks | Patients with mild cognitive impairment (MCI).  36 MCI patients were selected for the intervention.  The treatment group consisted of 18 MCI patients (8 males, 10 females) with average age of 61.50 ± 7.31 years and average educational history of 11.92 years.  The placebo group consisted of 18 MCI patients (6 males, 12 females) with average age of 61.67 ± 10.12 years and average educational history of 12.84 years. | Leukocytes | Concentration  of WYG phytonutrients in blood was not measured. | The MCI treatment group showed a significant 31% decrease (p < 0.05) in mtDNA deletion rate.  The frequency of mtDNA deletions was 8.58 % before treatment and 5.88% after treatment.  The mtDNA deletion rate in the placebo group did not change during the intervention.  WYG also improved MCI patients' memory function and the activity of serum SOD, and decreased the serum level of β-amyloid and malondialdehyde (biomarker of lipid peroxidation). | 7 |

**Supplementary Table 10 A:** Matrix summary of % change in levels of DNA damage biomarkers for interventions with single vitamins or single minerals and their combinations

| % Change in DNA Damage Biomarkers | | | | | | | |
| --- | --- | --- | --- | --- | --- | --- | --- |
|  | Chromosome Aberrations   [LY] | Micronuclei    [LY] | Micronuclei    [BC] | DNA Strand Breaks / Comet assay  [LY or PBMC] | DNA Oxidation*   [LY / PBMC] | Telomerase    [LY / PBMC] | Telomere length   [LY / PBMC] |
| *VitC (ascorbic acid)* | -40%  -70%  +0.2% | +14.8% | -29% | +20% | -54.9% |  |  |
| *VitA (retinol)* |  |  | -58% |  |  |  |  |
| *VitA (β-carotene)* |  |  | -61%  -60%  -75%  -27% | -4.8%  -35%  +86% | +36%  -57% |  |  |
| *VitB1 (thiamine)* |  | -31%  -15% |  |  |  |  |  |
| *VitB9 (folic acid)* |  | +11%  +6.3%  -35%  +340%  -30% | -71%  -15.4%  -31%  +300%  -57%  -73% |  |  |  |  |
| *VitB9 (folic acid) + VitB12 (cyanocobalamin)* |  | -15.3  -41%  -33% |  |  |  |  |  |
| *VitD* |  |  |  |  |  | +19.2% |  |
| *VitE (α-tocopherol)* |  | -0.30% | -80% | -9.7%  -8.3% | -10.9% |  |  |
| *Se (Selenite)* |  | -31% |  | +14.9% |  |  |  |
| *Se (Selenomethionine)* |  | +2.3% |  | -15.9% |  |  |  |
| *Zinc (zinc carnosine)* |  | -24% |  | -8.8% |  |  | +7.4% |
| *βCar+VitA* |  |  | -71% |  |  |  |  |
| *VitA+VitC* |  |  | -40% |  |  |  |  |
| *VitA+VitB2+Zinc* |  |  | -11.4% |  |  |  |  |
| *VitA+VitC+VitE+Se* |  | -54%  +0.4%  -24%  -13% |  |  |  |  |  |
| *βCar+VitC+VitE+Se* | -60% |  |  |  |  |  |  |
| *VitA+VitC+VitE+Zn* |  | -13% |  |  |  |  |  |
| *VitA+VitC+VitE+Folic acid+Rutin* |  | -25% |  |  |  |  |  |
| *Multi vitamin and mineral supplement* |  | -69% |  |  |  |  |  |

LY, lymphocytes; BC, buccal cells; PBMC, peripheral blood mononuclear cells.

* FPG/ENDO Comet or HPLC/MS assays

βCar, beta-carotene; Se, selenium; VitA, vitamin A; VitB1, vitamin B1; VitB2, vitamin B2; VitB9, vitamin B9; VitB12, vitamin B12; VitC, vitamin C; VitD, vitamin D; VitE, vitamin E; Zn, Zinc.

**Supplementary Table 10B:** Matrix summary of % change in levels of DNA damage biomarkers for interventions with phytonutrients, plant beverages or foods and their combinations

| \|  \| % Change in DNA Damage Biomarkers \| \| --- \| --- \| | | | | | | | |
| --- | --- | --- | --- | --- | --- | --- | --- | --- | --- |
|  | Chromosome Aberrations   [LY] | Micronuclei (Lympocyte)   [LY] | Micronuclei (Buccal)   [BC] | DNA Strand Breaks / Comet assay  [LY or PBMC] | DNA Oxidation*   [LY / PBMC] | Telomerase    [LY / PBMC] | Telomere length   [LY / PBMC] |
| Tomato juice | -0.1% | -24% |  |  | -26%  -35% |  |  |
| Lycopene rich drink |  |  |  | -13.8% |  |  |  |
| Lycopene |  |  |  | -43%  -8.9%  +38% | -23% |  |  |
| Tomato products |  |  |  | -24% |  |  |  |
| Tomato sauce |  |  |  |  | -28%  -29% |  |  |
| Tomato oleoresin |  |  |  |  | -21%  0% |  |  |
| Echinacea | -70% | -26% |  |  |  |  |  |
| Eugenol | -0.5% | +0.5% |  |  |  |  |  |
| Hesperidin |  |  |  |  | -23% |  |  |
| Soy isoflavones |  |  |  | -15% |  |  |  |
| Green Tea | -30% |  | -42% | -13%  -30% | -13.2% |  |  |
| Canthaxanthin |  |  | -0.5% |  |  |  |  |
| Lutein |  |  |  | -33%  -9.4% |  |  |  |
| Mixed carotenoids |  |  |  | -36% |  |  |  |
| Carotenoid capsule |  |  |  | +10% |  |  |  |
| Coffee |  |  |  | -44%  -27%  -12.5%  -14% | -44%  -14.1%  +16.1% |  |  |
| Grape seed procyanidins |  |  |  |  | -67% |  |  |
| Dealcoholised grape wine |  |  |  | -4.3% | +2.3% |  |  |
| Cocoa extract |  |  |  | +22% | -4.3% |  |  |
| Black currant juice |  |  |  | +38.4% | +40.6% |  |  |
| Red berry juice |  |  |  | -45%  -60% |  |  |  |
| Blue berry & apple juice |  |  |  | -20% |  |  |  |
| Purple grape juice |  |  |  | -25% |  |  |  |
| Grape & berry juice |  |  |  |  | -60%  -45% |  |  |
| Siberian Ginseng |  |  |  | -12% |  |  |  |
| Curcumin + piperine |  |  |  | -75% |  |  |  |
| Watercress |  |  |  | -23.9% | -23% |  |  |
| Kiwifruit golden |  |  |  | -34% | -34% |  |  |
| Kiwifruit |  |  |  | -13%  -25% | -25%  -29.5% |  |  |
| Olive oil |  |  |  |  | -18% |  |  |
| Docosa-hexaenoic acid (DHA) |  |  |  |  |  | -32% |  |
| N3 fatty acids |  |  |  |  |  | +0.5% | +0.8%  +21% |
| TA-65 phytonutrient |  |  |  |  |  |  | +5% |
| Vegetables & PUFA |  |  |  | -17% | -13%  -4% |  |  |
| Ascorbic acid rich diet |  |  |  | +5% |  |  |  |
| Antioxidant rich plant foods |  |  |  | -20% |  |  |  |
| Plant diet and phytonutrient and vitamin, mineral, supplements |  |  |  |  |  | +29%  -12.5% | +9.5% |
| Multi phytonutrient and vitamin supplement |  |  |  |  |  | +34% | +0.1% |

LY, lymphocytes; BC, buccal cells; PBMC, peripheral blood mononuclear cells
* (FPG/ENDO Comet or HPLC/MS assays)
